# Corallomycetellains A–J, Cytotoxic Epipolythiodioxopiperazine Alkaloids Isolated from the Fungi *Corallomycetella repens* HDN23-0007

**DOI:** 10.3390/md24020062

**Published:** 2026-02-01

**Authors:** Chenqi Zhang, Luning Zhou, Shuo Zhao, Wenxue Wang, Xiaomin Zhang, Qian Che, Tianjiao Zhu, Mei Han, Dehai Li

**Affiliations:** 1 School of Medicine and Pharmacy, Ocean University of China, Qingdao 266003, China; 18976072248@163.com (C.Z.); 18895692529@163.com (L.Z.); 13104496033@163.com (S.Z.); bx_wwx@163.com (W.W.); xiaominzhang91@163.com (X.Z.); cheqian064@ouc.edu.cn (Q.C.); zhutj@ouc.edu.cn (T.Z.); 2 Laboratory for Marine Drugs and Bioproducts, Qingdao Marine Science and Technology Center, Qingdao 266237, China; 3 Department of Pharmacology, School of Pharmacy, Qingdao University, Qingdao 266021, China

**Keywords:** Epipolythiodioxopiperazines, marine fungi, anticancer activity, GNPS

## Abstract

Ten new epipolythiodioxopiperazine (ETP) alkaloids, named corallomycetellains A–J (**1**–**10**), along with one known analogue, haematocin (**11**), were isolated from the fungi *Corallomycetella repens* HDN23-0007. Their structures, including absolute configurations, were established by comprehensive spectroscopic data and electronic circular dichroism (ECD) calculations. Compounds **1**–**2** represent the first two examples of aranotin-type ETPs possessing an aromatic indole moiety. Compounds **2**–**4** all featured a unique *C*2-methyl disulfide substituent, whereas compound **4** additionally possessed a *C*2′-oxomethyl group. In in vitro cytotoxicity assays, compounds **7**–**10**, which contained *α*–*α*′ polysulfide bridges, exhibited strong anticancer activity, with IC_50_ values ranging from 1.1 to 9.3 μM.

## 1. Introduction

Epipolythiodioxopiperazines (ETPs) represent a class of fungal secondary metabolites characterized by the presence of sulfur atoms within their diketopiperazine scaffold. The structural diversity of epipolythiodiketopiperazine (ETP) derivatives originates from the variability of diketopiperazine scaffolds, distinct patterns of sulfur atom linkage to the 2,5-diketopiperazine core, and extensive post-biosynthetic modifications. These compounds exhibit a wide range of biological activities, including antiviral (e.g., acetylaranotin) [1], antitumor (e.g., gliotoxin and chaetocin) [2,3,4,5], immunomodulatory (e.g., chetoseminudin A) [6], and antibacterial activities (e.g., sirodesmin PL) [7]. Critically, the biological activities of ETPs are primarily determined by the presence and spatial configuration of their characteristic disulfide bridges [8].

**Figure 1:**
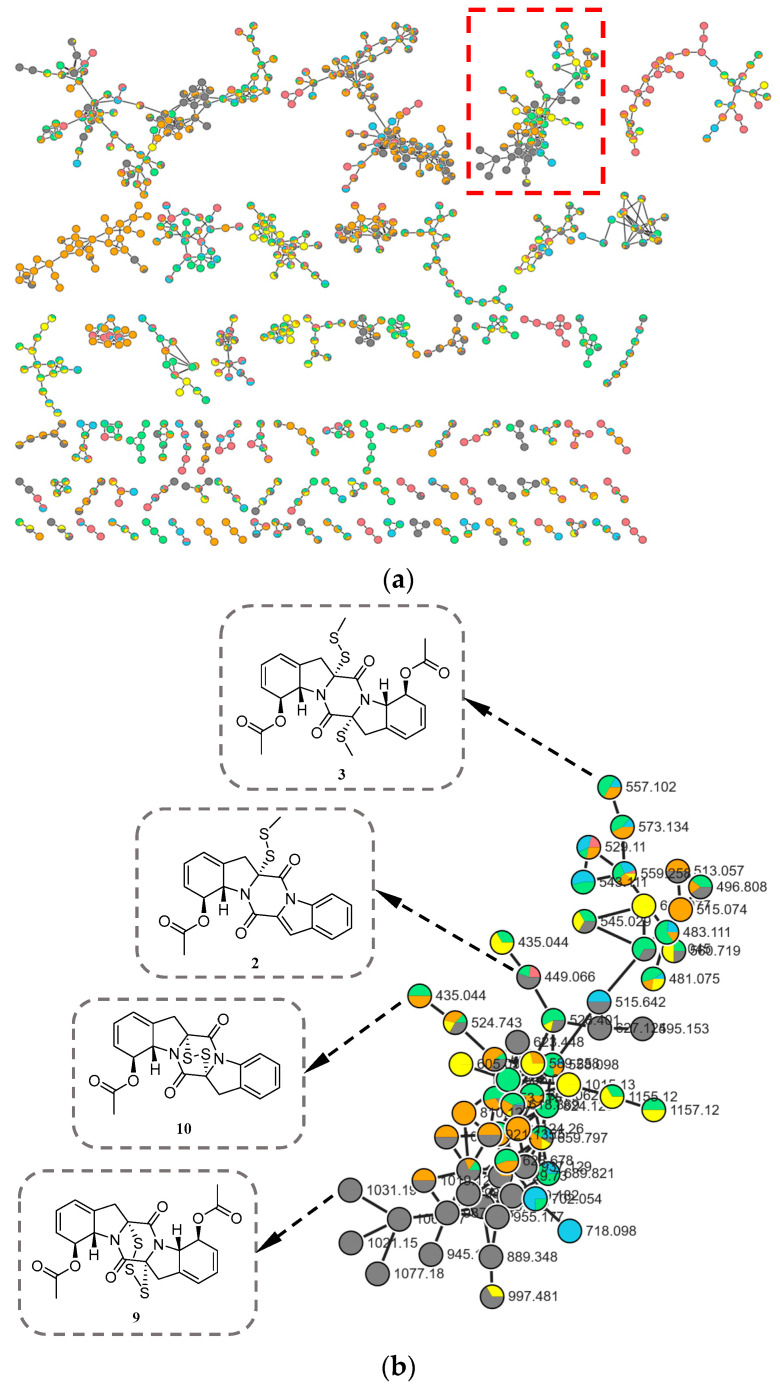
(**a**) Molecular network of six subfractions (colors correspond to different subfractions) from *Corallomycetella repens* HDN23-0007; (**b**) Clusters corresponding to compounds of the ETP derivatives observed in the molecular network (red box in (**a**)).

**Figure 2:**
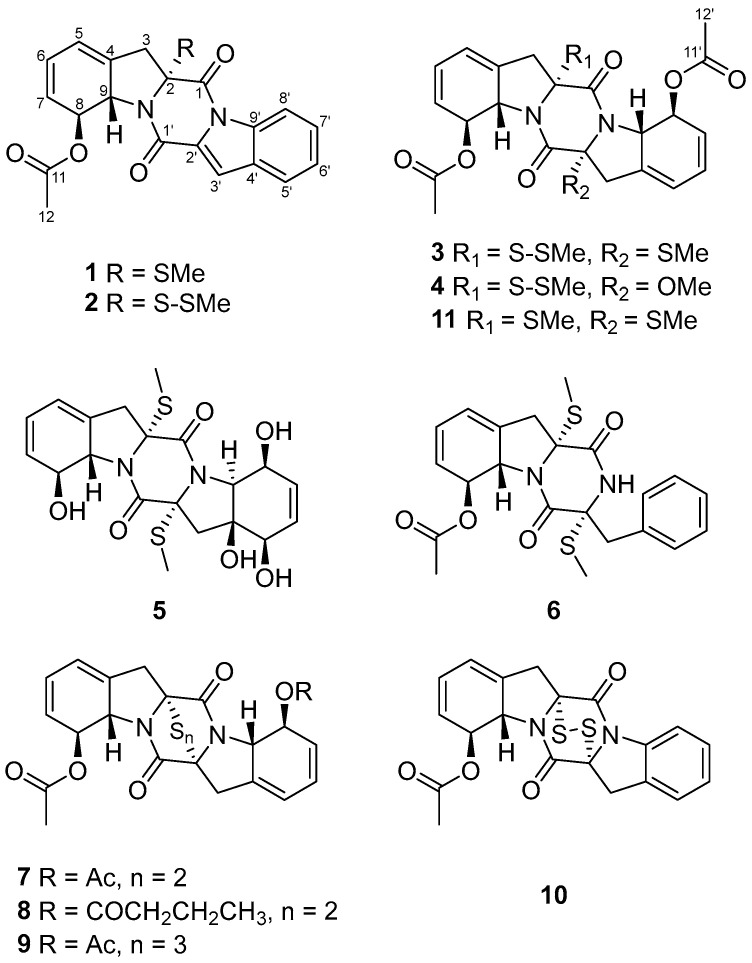
Structures of compounds **1**–**11**.

During our ongoing search for novel bioactive metabolites from marine-derived fungi [9,10], microbial fermentation extracts were initially analyzed by high-performance liquid chromatography–ultraviolet (HPLC–UV) profile. Among these, *Corallomycetella repens* HDN23-0007 was prioritized for further investigation due to its notably complex HPLC–UV profile, which displayed a high density of well-resolved, UV-absorbing peaks across a broad retention time range. A comprehensive analysis combining HPLC–UV profiling, liquid chromatography–mass spectrometry (LC–MS), and the Global Natural Products Social Molecular Networking (GNPS) platform (Figure 1) revealed the presence of potential epidithiodioxopiperazine (ETP) derivatives in its metabolome [11,12]. Subsequently, ten new epipolythiodioxopiperazine (ETP) alkaloids, named corallomycetellains A–J (**1**–**10**), along with one known analogue, haematocin (**11**), were isolated (Figure 2). Compounds **7**–**10** exhibited strong anticancer activity, with IC_50_ values ranging from 1.1 to 9.3 μM. In this paper, we describe their isolation, structural elucidation, and biological evaluation.

## 2. Results and Discussion

### 2.1. Structural Determination

Corallomycetellain A (**1**) was obtained as a light-yellow amorphous powder. Its molecular formula was determined to be C_21_H_18_N_2_O_4_S, based on the molecular ion at *m/z* 417.0880 [M + Na]^+^ observed in HRESIMS, indicating 14 degrees of unsaturation. The 1D NMR (Table 1) and HSQC spectra of **1** revealed the presence of one methylthio group (*δ*_C/H_ 13.1/2.09), one acetyl methyl group (*δ*_C/H_ 21.6/2.23), one methylene (*δ*_C/H_ 40.6/3.10), ten methines (one oxygenated (*δ*_C/H_ 74.9/6.31), one nitrogenated (*δ*_C/H_ 64.7/5.27), and eight olefinic ones (*δ*_C/H_ 115.0/7.45, *δ*_C/H_ 116.4/8.44, *δ*_C/H_ 120.6/6.03, *δ*_C/H_ 122.8/7.70, *δ*_C/H_ 125.3/6.03, *δ*_C/H_ 125.6/7.41, *δ*_C/H_ 128.2/7.54, *δ*_C/H_ 128.5/5.65)), one quaternary sp^3^ carbon (*δ*_C_ 76.4), four olefinic nonprotonated carbons (*δ*_C_ 128.9, 129.3, 133.9, 134.7), one ketone carbonyl (*δ*_C_ 171.4) and two amide carbonyls (*δ*_C_ 163.7, 155.7). Nine of the compound’s fourteen degrees of unsaturation were accounted for by the structural features identified so far, suggesting that compound **1** possessed a penta-cyclic core.

**Figure 3:**
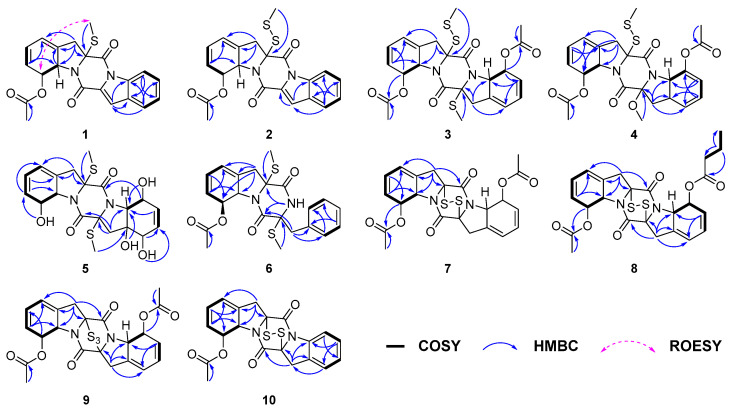
Key COSY, HMBC and ROESY correlations of compounds **1**–**10**.

A series of the COSY correlations from H-6 (*δ*_H_ 6.03)/H-7 (*δ*_H_ 5.65) and H-8 (*δ*_H_ 6.31)/H-9 (*δ*_H_ 5.27) as well as the HMBC correlations from H-7 to C-5 (*δ*_C_ 120.6)/C-9 (*δ*_C_ 64.7), from H-9 to C-4 (*δ*_C_ 133.9) and from H_2_-3 to C-2 (*δ*_C_ 76.4)/C-4/C-5/C-9 indicated the presence of six membered rings combined with five membered rings in **1**. (Figure 3). While the right series of the COSY correlations from H-5′ (*δ*_H_ 7.70)/H-6′ (*δ*_H_ 7.41) and H-7′ (*δ*_H_ 7.54)/H-8′ (*δ*_H_ 8.44) as well as the HMBC correlations from H-5′ to C-3′ (*δ*_C_ 115.0)/C-9′ (*δ*_C_ 134.7)/C-7′ (*δ*_C_ 128.2), from H-8′ to C-4′ (*δ*_C_ 129.3)/C-6′ (125.6) and from H-3′ (*δ*_H_ 7.45) to C-2′ (*δ*_C_ 128.9)/C-9′ revealed an extra six membered rings combined with five membered rings (Figure 3). Moreover, the compound was deduced to possess a 6-5-6-5-6 fused diketopiperazine (DKP) skeleton, based on the characteristic chemical shifts of the amide carbonyls (*δ*_C_ 163.7, C-1; *δ*_C_ 155.7, C-1′) and the molecular weight. Analysis of the NMR data (Table 1) for the left C-11 (*δ*_C_ 171.4) and C-12 (*δ*_C_ 21.6) of **1** and HMBC correlation from H-12 (*δ*_H_ 2.23) to C-11 indicated the presence of an acetyl group. Based on all relevant signals and chemical shifts, we hypothesized that the acetyl group can only form an ester bond with the oxygen at the C-8 (*δ*_C_ 74.9) position. Therefore, the planer structure of **1** with the presence of a 6/5/6/5/6 diketopiperazine skeleton was suggested, as shown in Figure 2.

**Figure 4:**
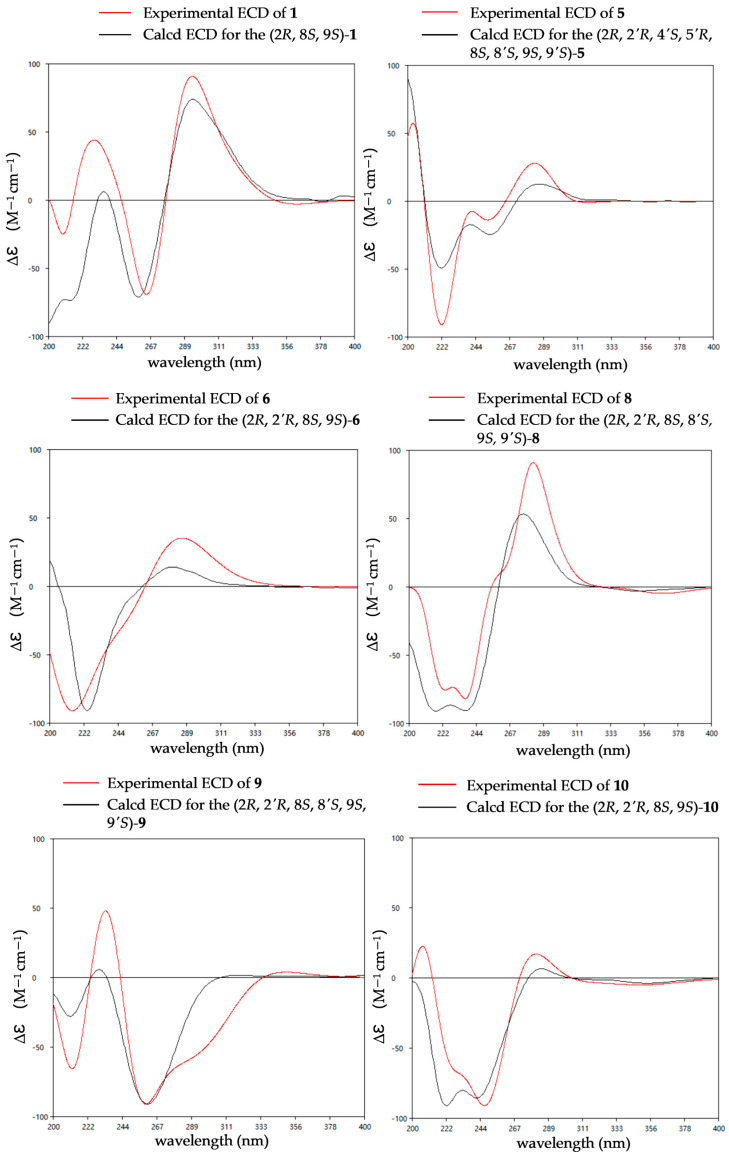
Experimental and calculated ECD spectra of compounds **1**, **5**–**6** and **8**–**10**.

The relative configurations of **1** were established as 2R*,8S*,9S* by the coupling constants analysis of H-8/H-9 (^3^*J*_H-8, H-9_ = 14.0 Hz) and the ROESY correlation between SMe-2 (*δ*_H_ 2.09) and H-8 (*δ*_H_ 6.31). To determine the absolute configurations of **1**, ECD calculations were performed at the B3LYP/6-31+G(d) level. The tendencies of the experimental ECD spectrum were in reasonable agreement with the calculated one of (2R*,8S*,9S*)-**1** (Figure 4), establishing the absolute configuration as 2*R*, 8*S*, 9*S* in **1**.

The molecular formula of corallomycetellain B (**2**), obtained as a light-yellow amorphous powder, was C_21_H_18_N_2_O_4_S_2_, deduced by HRESIMS. Comparison of ^1^H and ^13^C NMR spectra (Table 1) of **1** and **2** revealed that they shared the same 6/5/6/5/6 diketopiperazine skeleton with an aromatic indole moiety. The only difference was an existence of a disulfide bond in C-2 (*δ*_C_ 78.9) in **2** supported by the HRESIMS results and chemical shift differences of Me-R-2 in **1** and **2** (*δ*_C_ 13.1 in **1** while *δ*_C_ was 23.5 in **2**).

**Figure 5:**
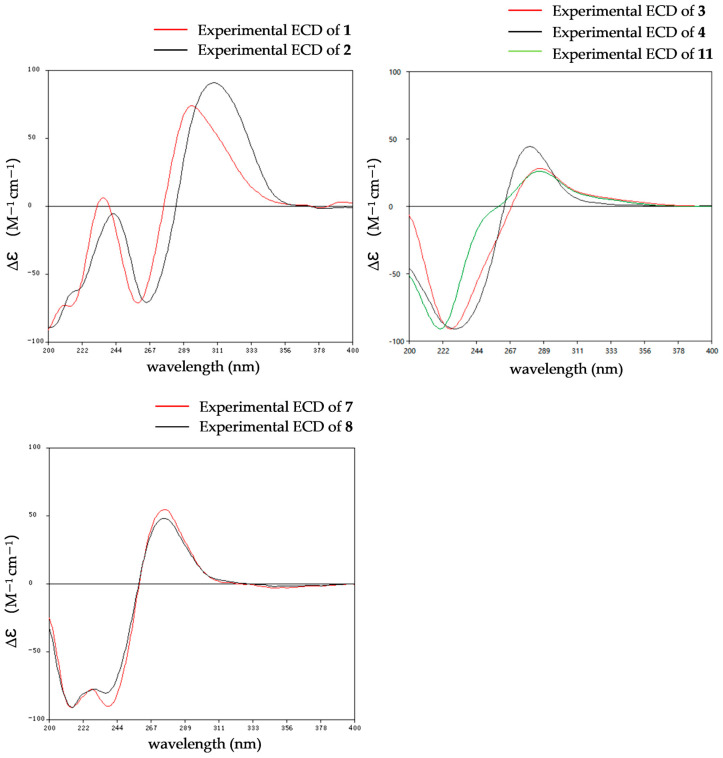
Experimental ECD spectra of **1**–**4**, **7**–**8** and **11**.

The relative configurations of **2** were established in the same way as **1** by the coupling constants analysis of H-8/9 (^3^*J*_H-8, H-9_ = 14.0 Hz) and the comparison of 1D NMR data with **1** (Table 1). Consequently, the absolute configuration of **2** was determined as 2*R*, 8*S*, 9*S*, since the experimental ECD curves of **1** and **2** agreed perfectly (Figure 5). Reported ETPs typically feature a sulfur-bridged diketopiperazine core, frequently integrated with fused oxazine rings. In comparison, compounds **1**–**2** represent the first two examples of aranotin-type ETPs possessing a rare aromatic indole unit (Figure S2).

Corallomycetellain C (**3**) and corallomycetellain D (**4**) were obtained as a light-yellow amorphous powders with the molecular formulae of C_24_H_26_N_2_O_6_S_3_ and C_24_H_26_N_2_O_7_S_2_, deduced by HRESIMS, respectively. Analysis of the ^1^H and ^13^C NMR data of **3** with those of haematocin (**11**) [13] indicated that **3** has the same skeleton as **11** (Table 2). The difference in mass of 32 Da between **3** and **11** indicated an addition of a sulfur atom in **3**, which is supported by the HRESIMS analysis. The HMBC correlation from S-SMe-2 (*δ*_H_ 23.8) to C-2 (*δ*_C_ 77.4), along with the differences in chemical shifts of C-2 and S-SMe-2 (*δ*_C_ 74.2 and *δ*_C_ 14.5 in **11** [13], compared to *δ*_C_ 77.4 and *δ*_C_ 23.8 in **3**), revealed the presence of a disulfide bond at C-2 in **3** (Figure 3). Compound **4** had a methoxy group instead of a methylthio group in C-2′, which was supported by chemical shifts of OMe-2′ (*δ*_C_ 53.3 and *δ*_H_ 3.50) and C-2′ (*δ*_C_ 95.4) as well as an HMBC correlation from OMe-2′ to C-2′ (Figure 3).

**Figure 6:**
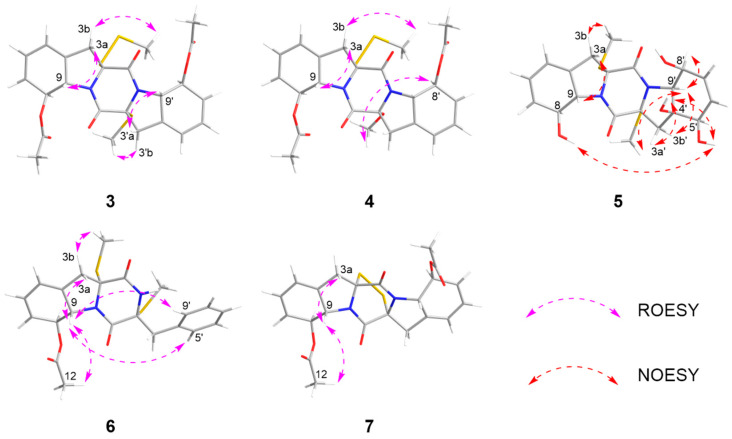
NOESY and ROESY correlations of **3**–**7**.

The relative configurations of **3**–**4** were established by ^1^H-^1^H coupling constants and ROESY data, and the comparison of 1D NMR data with haematocin (**11**). The coupling constants between H-8/8′ and H-9/9′ were more than 14.0 Hz, which revealed *trans* relationships of H-8/H-9 and H-8′/H-9′. The ROESY correlations from H-3a/3′a (*δ*_H_ 2.89) to H-9/9′ (*δ*_H_ 5.19), from H-3b (*δ*_H_ 3.69) to S-SMe-2 (*δ*_H_ 2.47) and from H-3′b (*δ*_H_ 3.00) to SMe-2′ (*δ*_H_ 2.23) indicated that S-SMe-2/SMe-2′ and H-9/9′ were located on the opposite side of the pyrrole ring (Figure 6). Therefore, the relative configuration of **3** was suggested. According to the ROESY correlations from H-3a (*δ*_H_ 2.89) to H-9 (*δ*_H_ 5.16), from H-3b (*δ*_H_ 3.67) to S-SMe-2 (*δ*_H_ 2.51), from H-8 (*δ*_H_ 5.86) to OMe-2′ (*δ*_H_ 3.50) (Figure 6) and the coupling constants analysis of H-8′/9′ (^3^*J*_H-8′, H-9′_ = 14.0 Hz), the relative configurations of **4** were established to be the same as **3**. The experimental ECD curves for **3**–**4** and haematocin (**11**) agreed perfectly (Figure 5), which demonstrated that their absolute configurations were 2*R*,2′*R*,8*S*,8′*S*,9*S*,9′*S*.

Corallomycetellain E (**5**), obtained as yellow oil, had a molecular formula of C_20_H_24_N_2_O_6_S_2_ deduced by an HRESIMS peak at *m/z* 453.1139 [M + H]^+^. Comparison of ^1^H and ^13^C NMR data of **5** (Table 3) to those of rostratazine B [14] revealed that **5** shared the same skeleton with rostratazine B. The difference was the replacement of a hydroxyl group at C-4′ (*δ_C_* 69.8) in **5**, supported by the HMBC correlation from OH-4′ (*δ*_H_ 5.32) to C-9′ (*δ_C_* 68.2) (Figure 3). Therefore, the planer structure of **5** was proposed.

The relative configuration of compound **5** was assigned based on NOESY data and coupling constant analysis. For the left moiety, the relative configuration of 2R*,8S*,9S* was determined from the following key observations: NOE correlations between H-3a (*δ*_H_ 2.88) and H-9 (*δ*_H_ 4.78), and between H-3b (*δ*_H_ 3.08) and SMe-2 (*δ*_H_ 2.16), together with a large coupling constant between H-8 and H-9 (^3^*J*_H-8, H-9_ = 13.0 Hz) (Figure 6). For the right moiety, a *cis* relationship between H-8′ and H-9′ was indicated by their smaller coupling constant (^3^*J*_H-8′, H-9′_ = 9.6 Hz). Furthermore, the relative configuration was confirmed by a network of NOE correlations: H-3′a (*δ*_H_ 2.92)/OH-4′ (*δ*_H_ 5.32), OH-4′ (*δ*_H_ 5.32)/OH-5′ (*δ*_H_ 4.88), OH-5′ (*δ*_H_ 4.88)/OH-8 (*δ*_H_ 5.27), H-3′b (*δ*_H_ 2.50)/H-9′ (*δ*_H_ 3.96), H-9′ (*δ*_H_ 3.96)/SMe-2′ (*δ*_H_ 2.13) (Figure 6). Therefore, the relative configurations of **5** were proposed as 2R*,2′R*,4′S*,5′R*,8S*,8′S*,9S*,9′S*. Consequently, the absolute configurations of **5** were determined as 2*R*,2′*R*,4′*S*,5′*R*,8*S*,8′*S*,9*S*,9′*S* by comparing the experimental electronic circular dichroism (ECD) curve with the calculated one (Figure 4).

Corallomycetellain F (**6**) was isolated as colorless oil with a molecular formula of C_22_H_24_N_2_O_4_S_2_ deduced by a HRESIMS peak at *m/z* 467.1070 [M + Na]^+^. Comparison of the ^1^H and ^13^C NMR data (Table 4) to those of phomazine B revealed that **6** shared the same skeleton with phomazine B [15]. The difference was the replacement of an acetoxyl group at C-6 (*δ*_C_ 74.9) in **6**, supported by the HMBC correlation from H-12 (*δ*_H_ 2.19) to C-11 (*δ_C_* 171.1) (Figure 3).

The relative configuration of **6** was established by ^1^H-^1^H coupling constants and ROESY data. The large *J* value (14.3 Hz) between H-9 (*δ*_H_ 4.90) and H-8 (*δ*_H_ 6.22) indicated the *trans*-orientations of H-8 and H-9, which was further supported by the observed ROE correlation between H-9 and H-12 (*δ*_H_ 2.19). In addition, ROESY correlations between H-9 and H-3a (*δ*_H_ 1.56), between H-9 and H-5′/8′ (*δ*_H_ 7.15) and between SMe-2 (*δ*_H_ 2.26) and H-3b (*δ*_H_ 2.58) (Figure 6) allowed the assignment of the two thiomethyl groups as *cis* to each other and both *trans* relative to H-9. Hence, the relative of **6** was assigned as 2R*,2′R*,8S*,9S*. To determine the absolute configuration of **6**, ECD calculations were performed at the B3LYP/6-31+G(d) level. The overall pattern of the experimental ECD spectrum was in reasonable agreement with the calculated one of (2*R*,2′*R*,8*S*,9*S*)-**6**, indicating the absolute configuration of C-2, C-2′, C-8 and C-9 in **6** as 2*R*,2′*R*,8*S*,9*S* (Figure 4).

Corallomycetellains G–I (**7**–**9**), isolated as white powder, have the molecular formulae of C_22_H_20_N_2_O_6_S_2_, C_24_H_24_N_2_O_6_S_2_ and C_22_H_20_N_2_O_6_S_3_, respectively, deduced by HRESIMS. The ^1^H and ^13^C NMR spectra of compound **7** revealed 10 proton signals and 11 carbon signals, precisely half of the total counts indicated by their molecular formulae. This observation suggests that **7** possesses a symmetrical structure. Comparative analysis of ^1^H and ^13^C NMR data (Table 2 and Table 5) of **7** and **3** confirmed that both share an identical 6/5/6/5/6 diketopiperazine core. The structural distinction was the presence of an additional disulfide bridge between C-2 and C-2′ in **7**. The planar structure of **7** was further elucidated based on 2D NMR correlations. Key COSY correlations were observed between H-6 (*δ*_H_ 5.98) and H-7 (*δ*_H_ 5.55) and between H-8 (*δ*_H_ 6.04) and H-9 (*δ*_H_ 4.98). Additionally, HMBC correlations from H-3 to C-1 (*δ*_C_ 162.8), C-2 (*δ*_C_ 78.2), C-4 (*δ*_C_ 132.3), C-5 (*δ*_C_ 119.8) and C-9 (*δ*_C_ 64.3); from H-7 to C-5 and C-9; from H-6 to C-8 (*δ*_C_ 74.1); and from both H-8 and H-12 (*δ*_H_ 2.15) to C-11 (*δ*_C_ 170.6) collectively supported the proposed connectivity, as illustrated in Figure 3.

The key structural difference between **7** and **8** was the presence of a butanoyl group at C-8′ (*δ*_C_ 74.3) in **8** instead of an acetoxy group in **7**, which was supported by the HMBC correlations from H-13′ (*δ*_H_ 1.66) to C-12′ (*δ*_C_ 35.9), C-11′ (*δ*_C_ 173.3) and C-14′ (*δ*_C_ 132.1) (Figure 3). In contrast to **7**, compound **9** was proposed to feature a trisulfide bridge. This inference was supported by HRESIMS data and by the distinct chemical shifts of C-2 and C-2′ in **9** (*δ*_C_ 78.2 and *δ*_C_ 82.4, respectively) compared to those in **7** (both *δ*_C_ 78.2).

Corallomycetellain G (**7**) was first isolated in 2001 from the fermentation broth filtrate of *Rhizostilbella* sp. [16] However, the relative configurations were not confirmed. Here, we determined the relative configurations of **7** based on the analysis of the coupling constants between H-8/8′ and H-9/9′ (^3^*J*_H-8/8′, H-9/9′_ = 13.2 Hz) as well as the ROESY correlations between H-3a (*δ*_H_ 3.70) and H-9 (*δ*_H_ 4.98) and between H-9 (*δ*_H_ 4.98) and H-12 (*δ*_H_ 2.15) (Figure 6). Comparison of 1D and 2D NMR spectra between **7** and **8**–**9** indicated the same relative configurations of them, supported by the coupling constants and chemical shifts (Table 5 and Table 6).

The comparison of the ECD curve of **7** [(218 (negative), 239 (negative), 276 (positive) and 359 nm (negative)] with those of emethallcin E [17] [(217 (negative), 237 (negative), 273 (positive), and 328 nm (negative)] confirmed that they had the same configuration around the epidithiodioxopiperazine ring. Therefore, the absolute configuration of **7** was determined as 2*R*,2′*R*,8*S*,8′*S*,9*S*,9′*S.* Based on the highly similar ECD spectra, the absolute configurations of **8** were proposed to be the same as those of **7** (Figure 5), which was further supported by computational calculations (Figure 4). The absolute configuration of **9** was determined as 2*R*,2′*R*,8*S*,8′*S*,9*S*,9′*S* by comparison of its experimental ECD spectrum with the calculated ones (Figure 4).

Corallomycetellain J (**10**) was obtained as a light-yellow amorphous powder with a molecular formula of C_20_H_16_N_2_O_4_S_2_ deduced by an HRESIMS peak at *m/z* 435.0432 [M + Na]^+^. The ^1^H and ^13^C NMR data of **10** revealed that **10** was similar to deoxyapoaranotin [18] (Table 6). The obvious difference was the presence of a 1,3-cyclohexadiene skeleton in ring A, supported by HMBC correlations from H-6 (*δ*_H_ 6.01) to C-4 (*δ*_C_ 132.1) and C-8 (*δ*_C_ 74.2) and from H-7 (*δ*_H_ 5.58) to C-5 (*δ*_C_ 120.0) and C-9 (*δ*_C_ 64.6), as well as a COSY correlation between H-8 (*δ*_H_ 6.14) and H-9 (*δ*_H_ 5.05) (Figure 3).

The coupling constants between H-8 and H-9 (^3^*J*_H-8, H-9_ = 18.0 Hz) indicated the *trans*orientation of H-8 and H-9. Comparison of the 1D NMR data for **10** with those of **7** (Table 5 and Table 6), particularly for positions C-1 through C-9 and C-1′ through C-3′, confirmed that they had the same configuration around the epidithiodioxopiperazine ring. Accordingly, the relative configurations of **10** were proposed as 2R*,2′R*,8S*,9S*. Comparing the ECD curve with the calculated ones (Figure 4), the absolute configuration of **10** was determined as 2*R*,2′*R*,8*S*,9*S*.

### 2.2. Biological Assays

The cytotoxicity of compounds **1**–**10** was evaluated against a panel of human cancer cell lines, including chronic myeloid leukemia (K562), pancreatic adenocarcinoma (ASPC-1 and MIA-PACA-2), small cell lung cancer (NCI-H446 and NCI-H446/EP) and pancreatic cancer (MIA-PACA-2 and MIA-PACA-2AR) as well as the human hepatocyte cell line (L-02) (Table 7). Compounds **7**–**10** possessed an *α*–*α*′ polysulfide bridge and exhibited strong anticancer activity with IC_50_ values ranging from 1.1 to 9.3 μM. Structure–activity relationship (SAR) analysis revealed that Compounds **7**–**8** and **10**, bearing a disulfide bridge, and compound **9**, containing a trisulfide bridge between C-2 and C-2′, showed stronger activity (IC_50_ = 1.1–5.2 μM) than **3** (IC_50_ = 25.8–27.0 μM), suggesting the crucial role of the *α–α*’ polysulfide linkage in cytotoxic potency. Among them, compound **10** exhibited the most potent activity, particularly against ASPC-1 and NCI-H446/EP cell lines (IC_50_ = 1.5–2.3 μM), surpassing the reference drug Doxorubicin (IC_50_ = 5.9–11.3 μM). Other compounds exhibited no inhibitory activity against the tested cancer cell lines.

## 3. Materials and Methods

### 3.1. General Experimental Procedures

Optical rotations were measured with a JASCO P-1020 digital polarimeter (JASCO Corporation, Tokyo, Japan). UV spectra were recorded on a Hitachi 5430 spectrophotometer (Hitachi Ltd., Tokyo, Japan). ECD spectra were measured on a JASCO J-715 (JASCO Corporation, Tokyo, Japan). NMR spectra were collected on Bruker AVANCE NEO 400 MHz spectrometer (Bruker, Karlsruhe, Germany), Agilent 500 MHz DD2 spectrometers (Agilent Technologies, Palo Alto, CA, USA) and a JNM-ECZ600R/S1 (JEOL Ltd., Tokyo, Japan). HRESIMS data were measured on a Thermo Scientific LTQ Orbitrap XL mass spectrometer (Thermo Fisher Scientific, Waltham, MA, USA). Sephadex LH-20 (Amersham Biosciences, Piscataway, NJ, USA) and silica gel (Qingdao Marine Chemical Factory, Qingdao, China) were used as stationary phases in column chromatography (CC). Semipreparative HPLC was performed on an ODS column (YMC-Pack ODS-A, YMC Co., Ltd., Fukuchiyama, Kyoto, Japan).

### 3.2. Fungal Material

Marine sediment collected from the Yellow River estuary, China (coordinates: 37°57′43″ N, 119°15′55″ E) in July 2023 was pretreated by ultrasonication for one minute. After suspending and diluting with seawater, the sediment was spread on three types of culture medium: PDA (Potato Dextrose Agar) medium, Rose Bengal medium and Sabouraud Dextrose Agar medium and cultured at 28 °C and 15 °C, respectively. The fungal strain HDN23-0007 was isolated from Rose Bengal medium at 28 °C and identified as *Corallomycetella repens* based on sequencing of the ITS region (GenBank No. PV628721) with 100% similarity. It was deposited at Key Laboratory of Marine Drugs, the Ministry of Education of China, School of Medicine and Pharmacy, Ocean University of China, Qingdao, People’s Republic of China.

### 3.3. Fermentation and Extraction

The fungal *Corallomycetella repens* HDN23-0007 was cultured in 1000 mL Erlenmeyer flasks containing 300 mL of malt culture medium (yeast extract 0.4%, malt extract 1%, glucose 0.4%) dissolved in naturally collected seawater (Huiquan Bay, Yellow Sea, Qiangdao, China). Fifty flasks were cultured under static conditions at 28 °C for 30 days. After 30 days of cultivation, the whole broth (15 L) was filtered and separated into supernatant and mycelia. The former was extracted three times with EtOAc, while the latter was extracted three times with methanol. All extracts were combined and evaporated under reduced pressure to give a crude extract (14.0 g).

### 3.4. Isolation and Purification

The crude extract was chromatographed over ODS, eluting with mixtures of MeOH/H_2_O to obtain five fractions (Fr.1 to Fr.5). Fr.1 and Fr.2 were separated on the Sephadex LH-20 column with MeOH to obtain Fr.1.1 and Fr.2.1. Fr.1.1 was further purified by semipreparative HPLC (20% ACN/H_2_O) to yield compound **5** (*t*_R_ = 9.8 min, 3.0 mg). Fr.2.1 was further separated by semipreparative HPLC (58% MeOH/H_2_O) to obtain Fr.2.1.1 and Fr.2.1.2. Fr.2.1.1 was further purified by semipreparative HPLC (62% ACN/H_2_O) to yield compound **3** (*t*_R_ = 10.5 min 6.0 mg). Fr.2.1.2 was further purified by semipreparative HPLC (50% ACN/H_2_O) to yield compound **4** (*t*_R_ = 15.0 min 3.0 mg).

Fr.3 was separated by semipreparative HPLC (58% MeOH/H_2_O) to obtain Fr.3.1 and Fr.3.2. Fr.3.1 was further purified by semipreparative HPLC (64% ACN/H_2_O), semipreparative HPLC (60% ACN/H_2_O) and semipreparative HPLC (52% ACN/H_2_O), respectively, to yield compounds **7** (*t*_R_ = 8.1 min 6.0 mg), **11** (*t*_R_ = 10.0 min 8.5 mg), **6** (*t*_R_ = 11.0 min 3.0 mg). Fr.3.2 was further purified by semipreparative HPLC (52% ACN/H_2_O) to yield compound **9** (*t*_R_ = 10.5 min 6.0 mg).

Fr.4 was separated by semipreparative HPLC (60% MeOH/H_2_O) to obtain Fr.4.1 and Fr.4.2. Fr.4.1 was further purified by semipreparative HPLC (52% ACN/H_2_O) to yield compound **1** (*t*_R_ = 13.8 min 2.5 mg). Fr.4.2 was further purified by semipreparative HPLC (58% ACN/H_2_O) to yield compound **10** (*t*_R_ = 11.2 min 3.0 mg). Fr.5 was separated by semipreparative HPLC (58% MeOH/H_2_O) to obtain Fr.5.1 and Fr.5.2. Fr.5.1 was further purified by semipreparative HPLC (64% ACN/H_2_O) to yield compound **8** (*t*_R_ = 9.8 min 3.0 mg). Fr.5.2 was further purified by semipreparative HPLC (62% ACN/H_2_O) to yield compound **2** (*t*_R_ = 12.0 min 3.0 mg).

Corallomycetellain A (**1**): light-yellow amorphous powder; ${\left[\alpha \right]}_{D}^{25}$ + 86.0 (*c* 0.5 mg/mL, MeOH); UV (MeOH) *λ*_max_: 249 nm; ECD (MeOH) *λ*_max_ 217 (−), 259 (−), 293 (+) nm; ^1^H and ^13^C NMR data, Table 1; HRESIMS *m/z* 417.0880 [M + Na]^+^ (calcd. for C_21_H_18_N_2_O_4_SNa, 417.0885).

Corallomycetellain B (**2**): light-yellow amorphous powder; ${\left[\alpha \right]}_{D}^{25}$ + 41.6 (*c* 0.5 mg/mL, MeOH); UV (MeOH) *λ*_max_: 253 nm; ECD (MeOH) *λ*_max_ 221 (−), 266 (−), 308 (+) nm; ^1^H and ^13^C NMR data, Table 1; HRESIMS *m/z* 449.0589 [M + Na]^+^ (calcd. for C_21_H_18_N_2_O_4_S_2_Na, 449.0606).

Corallomycetellain C (**3**): light-yellow amorphous powder; ${\left[\alpha \right]}_{D}^{25}$ − 36.0 (c 0.25 mg/mL, MeOH); UV (MeOH) *λ*_max_: 264 nm; ECD (MeOH) *λ*_max_ 227 (−), 285 (+) nm; ^1^H and ^13^C NMR data, Table 2; HRESIMS *m/z* 557.0835 [M + Na]^+^ (calcd. for C_24_H_26_N_2_O_6_S_3_Na, 557.0851).

Corallomycetellain D (**4**): light-yellow amorphous powder; ${\left[\alpha \right]}_{D}^{25}$ − 48.0 (c 0.25 mg/mL, MeOH); UV (MeOH) *λ*_max_: 263 nm; ECD (MeOH) *λ*_max_ 230 (−), 279 (+) nm; ^1^H and ^13^C NMR data, Table 2; HRESIMS *m/z* 541.1078 [M + Na]^+^ (calcd. for C_24_H_26_N_2_O_7_S_2_Na, 541.1079).

Corallomycetellain E (**5**): yellow oil; [*α*] ${\left[\alpha \right]}_{D}^{25}$ − 1.5 (c 0.5 mg/mL, MeOH); UV (MeOH) *λ*_max_: 211, 264 nm; ECD (MeOH) *λ*_max_ 222 (−), 256 (−), 285 (+) nm; ^1^H and ^13^C NMR data, Table 3; HRESIMS *m/z* 453.1139 [M + H]^+^ (calcd. for C_20_H_25_N_2_O_6_S_2_, 453.1154).

Corallomycetellain F (**6**): colorless oil; [*α*] ${\left[\alpha \right]}_{D}^{25}$ + 144.0 (c 0.5 mg/mL, MeOH); UV (MeOH) *λ*_max_: 216, 264 nm; ECD (MeOH) *λ*_max_ 226 (−), 280 (+) nm; ^1^H and ^13^C NMR data, Table 4; HRESIMS *m/z* 467.1070 [M + Na]^+^ (calcd. for C_21_H_18_N_2_O_4_SNa, 467.1075).

Corallomycetellain G (**7**): white powder; [*α*] ${\left[\alpha \right]}_{D}^{25}$ − 113.6 (c 0.5 mg/mL, MeOH); UV (MeOH) *λ*_max_: 265 nm; ECD (MeOH) *λ*_max_ 218 (−), 239 (−), 276 (+) 356 (−) nm. ^1^H and ^13^C NMR data, Table 5; HRESIMS *m/z* 495.0655 [M + Na]^+^ (calcd. for C_22_H_20_N_2_O_6_S_2_Na, 495.0660).

Corallomycetellain H (**8**): white powder; [*α*] ${\left[\alpha \right]}_{D}^{25}$ − 102.0 (c 0.5 mg/mL, MeOH); UV (MeOH) *λ*_max_: 264 nm; ECD (MeOH) *λ*_max_ 218 (−), 238 (−), 276 (+) 356 (−) nm; ^1^H and ^13^C NMR data, Table 5; HRESIMS *m/z* 523.0594 [M + Na]^+^ (calcd. for C_24_H_24_N_2_O_6_S_2_Na, 523.0973).

Corallomycetellain I (**9**): white powder; [*α*] ${\left[\alpha \right]}_{D}^{25}$ − 312.3 (c 0.3 mg/mL, MeOH); UV (MeOH) *λ*_max_: 268 nm; ECD (MeOH) *λ*_max_ 211 (−), 230 (+), 261 (−) nm; ^1^H and ^13^C NMR data, Table 6; HRESIMS *m/z* 527.0389 [M + Na]^+^ (calcd. for C_22_H_20_N_2_O_6_S_3_Na, 527.0381).

Corallomycetellain J (**10**): white powder; [*α*] ${\left[\alpha \right]}_{D}^{25}$ − 314.8 (c 0.5 mg/mL, MeOH); UV (MeOH) *λ*_max_: 212, 262 nm; ECD (MeOH) *λ*_max_ 221 (−), 242 (−), 285 (+) nm; ^1^H and ^13^C NMR data, Table 6; HRESIMS *m/z* 435.0432 [M + Na]^+^ (calcd. for C_20_H_16_N_2_O_4_S_2_Na, 435.0449).

Haematocin (**11**): white powder; ECD (MeOH) *λ*_max_ 219 (−), 285 (+) nm; ^1^H and ^13^C NMR data, Figures S91 and S92.

### 3.5. LC–MS/MS and Molecular Networking Analysis

Liquid chromatography–tandem mass spectrometry (LC–MS/MS) analyses were performed on a Dionex Ultimate 3000 UHPLC system (Thermo Scientific) coupled to a Q Exactive hybrid quadrupole-Orbitrap mass spectrometer (Thermo Scientific). Electrospray ionization was operated in negative-ion mode. The mobile phase comprised 0.1% (*v*/*v*) formic acid in ultrapure water (solvent A) and HPLC-grade acetonitrile (solvent B). The gradient was 0–1 min, 10% B; 1–23 min, linear increase to 100% B; 100% B held until 26 min; 26–30 min, return to 10% B and re-equilibration. The flow rate was 0.25 mL/min and the injection volume was 3 µL.

All MS/MS data were converted to mzXML format files by MSConvert software (v3.0.20169, ProteoWizard, Palo Alto, CA, USA). WinSCP as an FTP client was used to upload mzXML format files to GNPS for analysis. The desired files or entire folders were then assigned to different analysis groups (from G1 to G6). The molecular network was constructed using the GNPS data analysis workflow and algorithms. The network visualizations were completed through Cytoscape (v3.10.3; National Resource for Network Biology, San Diego, CA, USA).

### 3.6. ECD Calculations

Conformational searches were performed using the systematic protocol in Spartan’ 14 [19] with the Merck Molecular Force Field (MMFF). Each MMFF minimum was re-optimized at the B3LYP/6-31+G(d) level of density functional theory using Gaussian09 (D.01, Gaussian, Inc., Wallingford, CT, USA) [20] Optimizations were initiated from multiple starting conformers, and vibrational frequency analyses confirmed that all stationary points are true minima. For each configurational ensemble, the lowest energy conformers with Boltzmann populations > 5% were retained. Excited-state properties for these conformers were computed with time-dependent DFT (TD-DFT) for twenty singlet excited states; solvation in methanol was treated with the polarizable continuum model (PCM). ECD spectra were simulated with SpecDis [21]; each transition was convoluted with a Gaussian band shape (0.26 eV FWHM), and an overall blue or red shift was applied to match the experimental spectra.

### 3.7. In Vitro Cytotoxicity Assays

The cytotoxicity of compounds **1**–**10** against K562 cells was evaluated using the MTT assay, while their effects on a panel of adherent cell lines (L-02, ASPC-1, NCI-H446, NCI-H446/EP, MIA-PACA-2, and MIA-PACA-2AR) were assessed using the SRB assay. Doxorubicin was used as the positive control in all assays. The half-maximal inhibitory concentration (IC_50_) values were calculated by fitting the dose–response curves obtained after 72 h of continuous drug exposure. Data were presented as the mean from three independent experiments. The detailed methodologies for biological testing have been described in previous reports [22,23].

## 4. Conclusions

Ten new epipolythiodioxopiperazine (ETP) alkaloids, namely corallomycetellains A–J (**1**–**10**) and one known compound haematocin (**11**) were isolated from the marine sediment-derived fungi *Corallomycetella repens* HDN23-0007. Their structures, including absolute configurations, were elucidated by NMR, MS spectroscopic data and electronic circular dichroism (ECD) calculations. Among them, corallomycetellains A (**1**) and B (**2**) are the first reported aranotin-type ETPs containing aromatic indole moieties. In bioassays, corallomycetellains G–J (**7**–**10**), contained *α*–*α*′ polysulfide bridges, exhibited strong anticancer activity, with IC_50_ values ranging from 1.1 to 9.3 μM. Among them, corallomycetellain J (**10**) exhibited the most potent activity, particularly against ASPC-1 and NCI-H446/EP cell lines (IC_50_ = 1.5–2.3 μM), surpassing the reference drug Doxorubicin (IC_50_ = 5.9–11.3 μM). Overall, this study expands the chemical diversity of marine fungal metabolites and provides promising leads for the development of novel anticancer agents. It is worth noting that the ETP alkaloids, characterized by their sulfur bridges, often exhibit significant cytotoxicity. The potent activity observed in compounds **7**–**10** likely stems from the reactive polysulfide linkage, which can engage in redox cycling or cross-link with critical cellular thiols. While these effects contribute to their strong inhibitory impact on cancer cells, the potential for non-specific toxicity towards normal cells remains a challenge for their clinical translation. In terms of anti-angiogenesis, ETP-type molecules are known to disrupt the interaction between the transcription factor HIF-1α and its coactivator p300 by targeting the zinc-binding CH1 domain, suggesting that they could potentially inhibit tumor vascularization. Therefore, to mitigate these risks, future studies should focus on structural optimization, such as the development of prodrugs or targeted delivery systems to enhance tumor specificity.

## Figures and Tables

**Table 1:** ^1^H (500 MHz) and ^13^C (125 MHz) NMR data of **1**–**2** in CDCl_3_.

No.	1		2	
	*δ*_C_, Type	*δ*_H_ (*J* in Hz)	*δ*_C_, Type	*δ*_H_ (*J* in Hz)
1	163.7, C		163.6, C	
2	76.4, C		78.9, C	
3	40.6, CH_2_	3.10, d (15.8)	39.3, CH_2_	3.29, s
		3.22, d (15.0)		
4	133.9, C		132.9, C	
5	120.6, CH	6.03, m	121.0, CH	6.03, m
6	125.3, CH	6.03, m	125.0, CH	6.01, dd (9.7, 3.3)
7	128.5, CH	5.65, d (8.7)	128.9, CH	5.64, d (9.5)
8	74.9, CH	6.31, d (14.0)	74.6, CH	6.17, d (14.0)
9	64.7, CH	5.27, d (14.0)	64.7, CH	5.27, d (14.0)
11	171.4, C		171.0, C	
12	21.6, CH_3_	2.23, s	21.5, CH_3_	2.24, s
R-2	13.1, CH_3_	2.09, s	23.5, CH_3_	2.07, s
1′	155.7, C		155.8, C	
2′	128.9, C		129.6, C	
3′	115.0, CH	7.45, d (0.8)	114.7, CH	7.46, d (0.6)
4′	129.3, C		129.3, C	
5′	122.8, CH	7.70, dt (7.9, 1.0)	122.9, CH	7.71, d (7.8)
6′	125.6, CH	7.41, td (7.7, 1.1)	125.6, CH	7.41, td (8.1, 1.1)
7′	128.2, CH	7.54, ddd (8.3, 7.2, 1.2)	128.2, CH	7.53, ddd (8.4, 7.2, 1.2)
8′	116.4, CH	8.44, dt (8.6, 1.0)	116.1, CH	8.38, m
9′	134.7, C		135.1, C	

**Table 2:** ^1^H and ^13^C NMR data of **3**–**4** in CDCl_3_.

No.	3 ^a^		4 ^b^	
	*δ*_C_, Type	*δ*_H_ (*J* in Hz)	*δ*_C_, Type	*δ*_H_ (*J* in Hz)
1	165.8, C		166.6, C	
2	77.4, C		76.9, C	
3	38.3, CH_2_	2.89, m	38.5, CH_2_	2.89, m;
		3.69, d (15.9)		3.67, d (16.4)
4	133.7, C		133.2, C	
5	120.2, CH	5.94, m	120.4, CH	5.93, m
6	125.1, CH	5.95, m	125.2, CH	5.95, m
7	128.2, CH	5.58, t (6.4)	127.7, CH	5.57, m
8	75.2, CH	6.13, d (14.5)	74.9, CH	6.17, dd (14.6, 2.2)
9	64.6, CH	5.19, d (14.2)	65.1, CH	5.16, d (13.6)
11	170.3, C		170.9, C	
12	21.4, CH_3_	2.12, s	21.4, CH_3_	2.13, s
Me-R_1_-2	23.8, CH_3_	2.47, s	23.9, CH_3_	2.51, s
1′	164.9, C		163.7, C	
2′	74.2, C		95.4, C	
3′	39.7, CH_2_	2.89, m;	41.1, CH_2_	2.61, m;
		3.00, d (16.0)		3.04, d (15.3)
4′	133.5, C		134.0, C	
5′	120.2, CH	5.94, m	119.3, CH	5.91, m
6′	124.8, CH	5.95, m	125.2, CH	5.95, m
7′	128.1, CH	5.58, t (6.4)	128.1, CH	5.57, m
8′	76.0, CH	6.03, d (13.1)	75.3, CH	5.86, d (13.9)
9′	63.8, CH	5.19, d (14.2)	63.4, CH	5.18, d (13.6)
11′	170.5, C		170.5, C	
12′	21.4, CH_3_	2.09, s	21.4, CH_3_	2.10, s
Me-R_2_-2′	14.4, CH_3_	2.23, s	53.3, CH_3_	3.50, s

^a^ Data recorded at ^1^H (500 MHz) and ^13^C (125 MHz) NMR. ^b^ Data recorded at ^1^H (600 MHz) and ^13^C (150 MHz) NMR.

**Table 3:** ^1^H (500 MHz) and ^13^C (125 MHz) NMR data of **5** in DMSO-*d*_6_.

No.	*δ*_C_, Type	*δ*_H_ (*J* in Hz)	No.	*δ*_C_, Type	*δ*_H_ (*J* in Hz)
1	164.9, C		2′	75.1, C	
2	73.0, C		3′	49.4, CH_2_	2.50, d (8.6)
3	38.8, CH_2_	2.88, d (16.1)			2.92, d (14.1)
		3.08, d (15.5)	4′	69.8, C	
4	133.5, C		5′	65.5, CH	4.07, d (3.9)
5	118.9, CH	5.98, dt (5.5, 2.9)	6′	127.8, CH	5.74, d (1.7)
6	123.3, CH	5.90, ddd (9.8, 4.8, 2.7)	7′	132.0, CH	5.75, d (1.0)
7	130.5, CH	5.62, d (9.9)	8′	67.8, CH	4.40, d (9.6)
8	73.9, CH	4.63, d (13.4)	9′	68.2, CH	3.96, ddd (10.4, 6.9, 3.6)
9	67.6, CH	4.78, d (13.7)	SMe-2′	14.3, CH_3_	2.13, s
SMe-2	14.0, CH_3_	2.16, s	OH-8′		4.32, d (7.2)
OH-8		5.27, d (1.7)	OH-4′		5.32, s
1′	167.2, C		OH-5′		4.88, d (4.8)

**Table 4:** ^1^H (500 MHz) and ^13^C (125 MHz) NMR data of **6** in CDCl_3_.

No.	*δ*_C_, Type	*δ*_H_ (*J* in Hz)	No.	*δ*_C_, Type	*δ*_H_ (*J* in Hz)
1	167.2, C		1′	164.5, C	
2	68.4, C		2′	73.5, C	
3	39.0, CH_2_	1.56, m	3′	46.6, CH_2_	2.95, d (14.3)
		2.58, d (15.8)			3.66, d (14.3)
4	133.6, C		4′	133.5, C	
5	119.7, CH	5.79, q (3.5)	5′	130.8, CH	7.15, dd (7.7, 1.8)
6	125.3, CH	5.92, dt (8.5 3.7)	6′	128.7, CH	7.28, m
7	128.0, CH	5.57, dd (9.9, 1.9)	7′	128.0, CH	7.28, m
8	74.9, CH	6.22, d (14.3)	8′	130.8, CH	7.15, dd (7.7, 1.8)
9	65.4, CH	4.90, d (14.3)	9′	128.7, CH	7.28, m
11	171.1, C		SMe-2′	13.7, CH_3_	2.36, s
12	21.5, CH_3_	2.19, s	NH		6.70, s
SMe-2	14.6, CH_3_	2.26, s			

**Table 5:** ^1^H (600 MHz) and ^13^C (150 MHz) NMR data of **7** (CDCl_3_) and **8** (CD_3_OD).

No.	7		8	
	*δ*_C_, Type	*δ*_H_ (*J* in Hz)	*δ*_C_, Type	*δ*_H_ (*J* in Hz)
1	162.8, C		162.9, C	
2	78.2, C		78.7, C	
3	36.4, CH_2_	2.88, d (17.8)	35.8, CH_2_	2.97, m
		3.70, d (17.8)		3.68, m
4	132.3, C		133.2, C	
5	119.8, CH	5.98, m	119.1, CH	6.05, m
6	124.5, CH	5.98, m	124.6, CH	6.04, m
7	127.7, CH	5.55, dd (11.6, 4.4)	126.7, CH	5.52, m
8	74.1, CH	6.04, d (12.3)	74.1, CH	5.93, d (13.2)
9	64.3, CH	4.98, d (13.5)	64.1, CH	5.12, d (13.3)
11	170.6, C		171.0, C	
12	21.3, CH_3_	2.15, s	20.0, CH_3_	2.08, s
1′	162.8, C		162.8, C	
2′	78.2, C		78.7, C	
3′	36.4, CH_2_	2.88, d (17.8)	35.8, CH_2_	2.97, m
		3.70, d (17.8)		3.68, m
4′	132.3, C		133.2, C	
5′	119.8, CH	5.98, m	119.1, CH	6.05, m
6′	124.5, CH	5.98, m	124.6, CH	6.04, m
7′	127.7, CH	5.55, dd (11.6, 4.4)	126.8, CH	5.52, m
8′	74.1, CH	6.04, d (12.3)	74.3, CH	5.93, d (13.2)
9′	64.3, CH	4.98, d (13.5)	64.2, CH	5.12, d (13.3)
11′	170.6, C		173.3, C	
12′	21.3, CH_3_	2.15, s	35.9, CH_2_	2.38, td (7.4 2.8)
13′			17.7, CH_2_	1.66, h (7.4)
14′			12.7, CH_3_	0.96, s

**Table 6:** ^1^H (600 MHz) and ^13^C (150 MHz) NMR data of **9**–**10** (in CDCl_3_).

No.	9		10	
	*δ*_C_, Type	*δ*_H_ (*J* in Hz)	*δ*_C_, Type	*δ*_H_ (*J* in Hz)
1	163.9, C		161.0, C	
2	78.2, C		78.1, C	
3	40.0, CH_2_	2.86, d (16.8);	36.3, CH_2_	3.00, d (17.9)
		3.28, m		3.82, d (17.7)
4	131.2, C		132.1, CH	
5	120.7, CH	5.93, dt (5.2, 2.3)	120.0, CH	6.04, m
6	124.3, CH	5.93, dt (5.2, 2.3)	124.4, CH	6.01, m
7	128.0, CH	5.56, m	127.8, CH	5.58, d (9.8)
8	75.0, CH	6.22, t (14.0, 13.0)	74.2, CH	6.14, d (12.9)
9	64.9, CH	5.32, d (13.3)	64.6, CH	5.05, d (13.0)
11	170.5, C		170.7, C	
12	21.5, CH_3_	2.21, s	21.4, CH_3_	2.18, s
1′	164.9, C		162.2, C	
2′	82.4, C		76.6, C	
3′	41.8, CH_2_	2.92, dd (17.2)	36.1, CH_2_	3.26, d (18.4)
		3.33, m		4.26, d (18.5)
4′	131.9 C		138.2, C	
5′	120.4, CH	5.93, dt (5.2, 2.3)	125.7, CH	7.20, dd (7.2, 6.7)
6′	124.5, CH	5.93, dt (5.2, 2.3)	125.3, CH	7.33, dd (2.8, 2.4)
7′	128.6, CH	5.56, m	128.7, CH	7.33, dd (2.8, 2.4)
8′	74.7, CH	6.19, t (14.0, 13.0)	115.7, CH	7.93, d (8.2)
9′	63.9, CH	5.08, d (14.8)	128.3, C	
11′	170.5, C			
12′	21.4, CH_3_	2.16, s		

**Table 7:** Cytotoxicity against six cancer cell lines of **3** and **7**–**10** (IC_50_, μM).

No.	IC_50_ (μM)						
	K562	L-02	ASPC-1	NCI-H446	NCI-H446/EP	MIA-PACA-2 ^a^	MIA-PACA-2AR ^a^
**3**	25.8	18.4	>30.0	27.0	>30.0	-	-
**7**	4.9	2.1	4.2	5.1	9.3	-	-
**8**	1.4	3.5	1.9	4.0	5.2	4.8	2.7
**9**	1.9	1.2	2.9	3.4	3.4	-	-
**10**	1.1	1.8	1.5	2.7	2.3	3.5	2.7
Doxorubicin	0.3	0.3	5.9	0.8	11.3	0.8	0.3

^a^ Compounds **3**, **7** and **9** were not evaluated against the cancer cell lines MIA-PACA-2 and MIA-PACA-2AR.

## Data Availability

The original contributions presented in this study are included in the article/Supplementary Materials. Further inquiries can be directed to the corresponding authors.

## Supplementary Materials

The following supporting information can be downloaded at https://www.mdpi.com/article/10.3390/md24020062/s1, Figure S1: The pictures of sediment sample and *Corallomycetella repens* HDN23-0007; Figure S2: The structure of **1** and **2**, known aranotin-type ETPs and analogue; Figures S3–S89: The HRESIMS, 1D, 2D NMR, UV and CD spectra of compounds **1**–**10**; Figures S90–S93: The 1D NMR, CD and UPLC-MS spectra of **11**; Figures S94–S103: HPLC purity chromatogram of compounds **1**–**10**; Figure S104: Isolation of compounds from the extract of fungus HDN23-0007; Figure S105: Comparative HPLC analysis of corallomycetellains A–J (**1**–**10**) and ITS (Internal Transcribed Spacer) sequence data of the strain *Corallomycetella repens* HDN23-0007.
