# Supplementary material for: Corallomycetellains A–J, Cytotoxic Epipolythiodioxopiperazine Alkaloids Isolated from the Fungi Corallomycetella repens HDN23-0007
Source: Mar Drugs. 2026 Feb 1;24(2):62. doi: 10.3390/md24020062 (PMC12942383; doi:10.3390/md24020062)
Supplement: Supplementary file 1 [file marinedrugs-24-00062-s001.zip › marinedrugs-4109001-supplementary.pdf]

Supporting Information for

Corallomycetellains A−J, Cytotoxic Epipolythiodioxopiperazine Alkaloids Isolated from the Fungi *Corallomycetella repens* HDN23-0007

Chenqi Zhang [1,†], Luning Zhou [1,†], Shuo Zhao, Wenxue Wang [1], Xiaomin Zhang [1], Qian Che[1,2], Tianjiao Zhu [1,2], Mei Han[3,*] and Dehai Li [1,2,*]

[1] School of Medicine and Pharmacy, Key Laboratory of Marine Drugs Ministry of Education, Sanya Oceanographic Institute, Frontiers Science Center for Deep Ocean Multispheres and Earth System, Ocean University of China, Qingdao 266000, China;

[2] Laboratory for Marine Drugs and Bioproducts, Qingdao Marine Science and Technology Center, Qingdao 266237, China

[3] Department of Pharmacology, School of Pharmacy, Qingdao University, Qingdao 266021, China

[*] Correspondence: dehaili@ouc.edu.cn (D.L.), hanmei@qdu.edu.cn (M.H.)

† These authors contributed equally to this work.

Contents

Figure S1. The pictures of sediment sample and *Corallomycetella repens* HDN23-0007. 1

Figure S2. The structure of 1 and 2, known aranotin-type ETPs and analogue. 1

Figure S3. HRESIMS spectrum of corallomycetellain A (**1**). 2

Figure S4. ^{1}H NMR (500 MHz, $CDCl_3$) spectrum of corallomycetellain A (**1**). 2

Figure S5. ^{13}C NMR (125 MHz, $CDCl_3$) spectrum of corallomycetellain A (**1**). 3

Figure S6. ^{1}H-^{1}H COSY spectrum of corallomycetellain A (**1**). 3

Figure S7. HSQC spectrum of corallomycetellain A (**1**). 4

Figure S8. HMBC spectrum of corallomycetellain A (**1**). 4

Figure S9. ROESY spectrum of corallomycetellain A (**1**). 5

Figure S10. The UV spectrum of corallomycetellain A (**1**). 5

Figure S11. The CD spectrum of corallomycetellain A (**1**). 6

Figure S12. The HRESIMS spectrum of corallomycetellain B (**2**). 6

Figure S13. ^{1}H NMR (500 MHz, $CDCl_3$) spectrum of corallomycetellain B (**2**). 7

Figure S14. ^{13}C NMR (125 MHz, $CDCl_3$) spectrum of corallomycetellain B (**2**). 7

Figure S15. ^{1}H-^{1}H COSY spectrum of corallomycetellain B (**2**). 8

Figure S16. HSQC spectrum of corallomycetellain B (**2**). 8

Figure S17. HMBC spectrum of corallomycetellain B (**2**). 9

Figure S18. ROESY spectrum of corallomycetellain B (**2**). 9

Figure S19. The UV spectrum of corallomycetellain B (**2**). 10

Figure S20. The CD spectrum of corallomycetellain B (**2**). 10

Figure S21. The HRESIMS spectrum of corallomycetellain C (**3**). 11

Figure S22. ^{1}H NMR (500 MHz, $CDCl_3$) spectrum of corallomycetellain C (**3**). 11

Figure S23. ^{13}C NMR (125 MHz, $CDCl_3$) spectrum of corallomycetellain C (**3**). 12

Figure S24. ^{1}H-^{1}H COSY spectrum of corallomycetellain C (**3**). 12

Figure S25. HSQC spectrum of corallomycetellain C (**3**). 13

Figure S26. HMBC spectrum of corallomycetellain C (**3**). 13

Figure S27. ROESY spectrum of corallomycetellain C (**3**). 14

Figure S28. The UV spectrum of corallomycetellain C (**3**). 14

Figure S29. The CD spectrum of corallomycetellain C (**3**). 15

Figure S30. The HRESIMS spectrum of corallomycetellain D (**4**). 15

Figure S31. ^{1}H NMR (600 MHz, $CDCl_3$) spectrum of corallomycetellain D (**4**) 16

Figure S32. ^{13}C NMR (150 MHz, $CDCl_3$) spectrum of corallomycetellain D (**4**) 16

Figure S33. ^{1}H-^{1}H COSY spectrum of corallomycetellain D (**4**). 17

Figure S34. HSQC spectrum of corallomycetellain D (**4**). 17

Figure S35. HMBC spectrum of corallomycetellain D (**4**). 18

Figure S36. ROESY spectrum of corallomycetellain D (**4**). 18

Figure S37. The UV spectrum of corallomycetellain D (**4**). 19

Figure S38. The CD spectrum of corallomycetellain D (**4**). 19
Figure S39. The HRESIMS spectrum of corallomycetellain E (**5**). 20
Figure S40. ^{1}H NMR (500 MHz, DMSO-d_6) spectrum of corallomycetellain E (**5**). 20
Figure S41. ^{13}C NMR (125 MHz, DMSO-d_6) spectrum of corallomycetellain E (**5**). 21
Figure S42. ^{1}H-^{1}H COSY spectrum of corallomycetellain E (**5**). 21
Figure S43. HSQC spectrum of corallomycetellain E (**5**). 22
Figure S44. HMBC spectrum of corallomycetellain E (**5**). 22
Figure S45. NOESY spectrum of corallomycetellain E (**5**). 23
Figure S46. The UV spectrum of corallomycetellain E (**5**). 23
Figure S47. The CD spectrum of corallomycetellain E (**5**). 24
Figure S48. The HRESIMS spectrum of corallomycetellain F (**6**). 24
Figure S49. ^{1}H NMR (500 MHz, $CDCl_3$) spectrum of corallomycetellain F (**6**). 25
Figure S50. ^{13}C NMR (125 MHz, $CDCl_3$) spectrum of corallomycetellain F (**6**). 25
Figure S51. ^{1}H-^{1}H COSY spectrum of corallomycetellain F (**6**). 26
Figure S52. HSQC spectrum of corallomycetellain F (**6**). 26
Figure S53. HMBC spectrum of corallomycetellain F (**6**). 27
Figure S54. ROESY spectrum of corallomycetellain F (**6**) 27
Figure S55. The UV spectrum of corallomycetellain F (**6**). 28
Figure S56. The CD spectrum of corallomycetellain F (**6**). 28
Figure S57. The HRESIMS spectrum of corallomycetellain G (**7**). 29
Figure S58. ^{1}H NMR (600 MHz, $CDCl_3$) spectrum of corallomycetellain G (**7**). 29
Figure S59. ^{13}C NMR (150 MHz, $CDCl_3$) spectrum of corallomycetellain G (**7**). 30
Figure S60. ^{1}H-^{1}H COSY spectrum of corallomycetellain G (**7**). 30
Figure S61. HSQC spectrum of corallomycetellain G (**7**). 31
Figure S62. HMBC spectrum of corallomycetellain G (**7**). 31
Figure S63. ROESY spectrum of corallomycetellain G (**7**). 32
Figure S64. The UV spectrum of corallomycetellain G (**7**). 32
Figure S65. The CD spectrum of corallomycetellain G (**7**). 33
Figure S66. The HRESIMS spectrum of corallomycetellain H (**8**). 33
Figure S67. ^{1}H NMR (600 MHz, $CDCl_3$) spectrum of corallomycetellain H (**8**). 34
Figure S68. ^{13}C NMR (150 MHz, $CDCl_3$) spectrum of corallomycetellain H (**8**) 34
Figure S69. ^{1}H-^{1}H COSY spectrum of corallomycetellain H (**8**). 35
Figure S70. HSQC spectrum of corallomycetellain H (**8**). 35
Figure S71. HMBC spectrum of corallomycetellain H (**8**). 36
Figure S72. The UV spectrum of corallomycetellain H (**8**). 36
Figure S73. The CD spectrum of corallomycetellain H (**8**). 37
Figure S74. The HRESIMS spectrum of corallomycetellain I (**9**). 37
Figure S75. ^{1}H NMR (600 MHz, $CDCl_3$) spectrum of corallomycetellain I (**9**). 38

Figure S76. ^{13}C NMR (150 MHz, $CDCl_3$) spectrum of corallomycetellain I (**9**). 38
Figure S77. ^{1}H-^{1}H COSY spectrum of corallomycetellain I (**9**). 39
Figure S78. HSQC spectrum of corallomycetellain I (**9**). 39
Figure S79. HMBC spectrum of corallomycetellain I (**9**). 40
Figure S80. The UV spectrum of corallomycetellain I (**9**). 40
Figure S81. The CD spectrum of corallomycetellain I (**9**). 41
Figure S82. HRESIMS spectrum of corallomycetellain J (**10**). 41
Figure S83. ^{1}H NMR (600 MHz, $CDCl_3$) spectrum of corallomycetellain J (**10**). 42
Figure S84. ^{13}C NMR (150 MHz, $CDCl_3$) spectrum of corallomycetellain J (**10**). 42
Figure S85. ^{1}H-^{1}H COSY spectrum of corallomycetellain J (**10**). 43
Figure S86. HSQC spectrum of corallomycetellain J (**10**). 43
Figure S87. HMBC spectrum of corallomycetellain J (**10**). 44
Figure S88. The UV spectrum of corallomycetellain J (**10**). 44
Figure S89. The CD spectrum of corallomycetellain J (**10**). 45
Figure S90. ^{1}H NMR (600 MHz, $CDCl_3$) spectrum of haematocin (**11**). 45
Figure S91. ^{13}C NMR (150 MHz, $CDCl_3$) spectrum of haematocin (**11**). 46
Figure S92. The CD spectrum of haematocin (**11**). 46
Figure S93. The UPLC-MS spectrum of haematocin (**11**). 47
Figure S94. HPLC purity chromatogram of **1**. 48
Figure S95. HPLC purity chromatogram of **2**. 48
Figure S96. HPLC purity chromatogram of **3**. 49
Figure S97. HPLC purity chromatogram of **4**. 49
Figure S98. HPLC purity chromatogram of **5**. 50
Figure S99. HPLC purity chromatogram of **6**. 50
Figure S100. HPLC purity chromatogram of **7**. 51
Figure S101. HPLC purity chromatogram of **8**. 51
Figure S102. HPLC purity chromatogram of **9**. 52
Figure S103. HPLC purity chromatogram of **10**. 52
Figure S104. Isolation of compounds from the extract of fungus HDN23-0007 53
Figure S105. Comparative HPLC analysis of corallomycetellains A-J (**1**-**10**). 53
The ITS gene sequences data of Corallomycetella repens HDN23-0007. 54

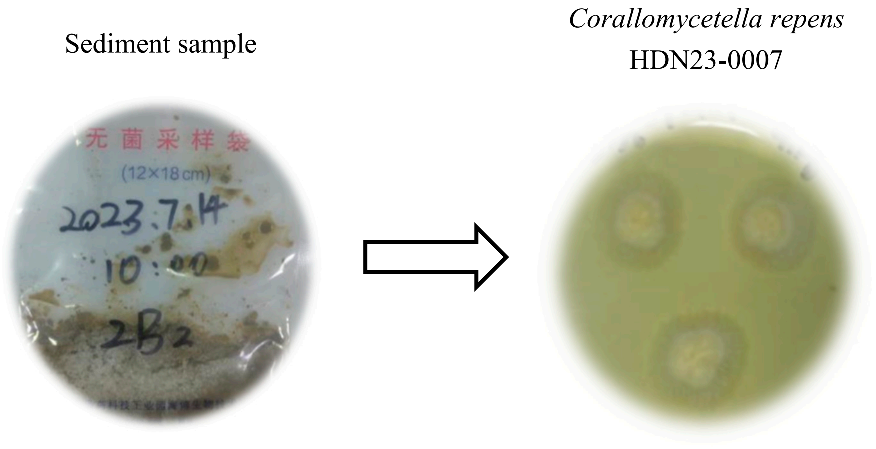

Figure S1. The pictures of sediment sample and *Corallomycetella repens* HDN23-0007.

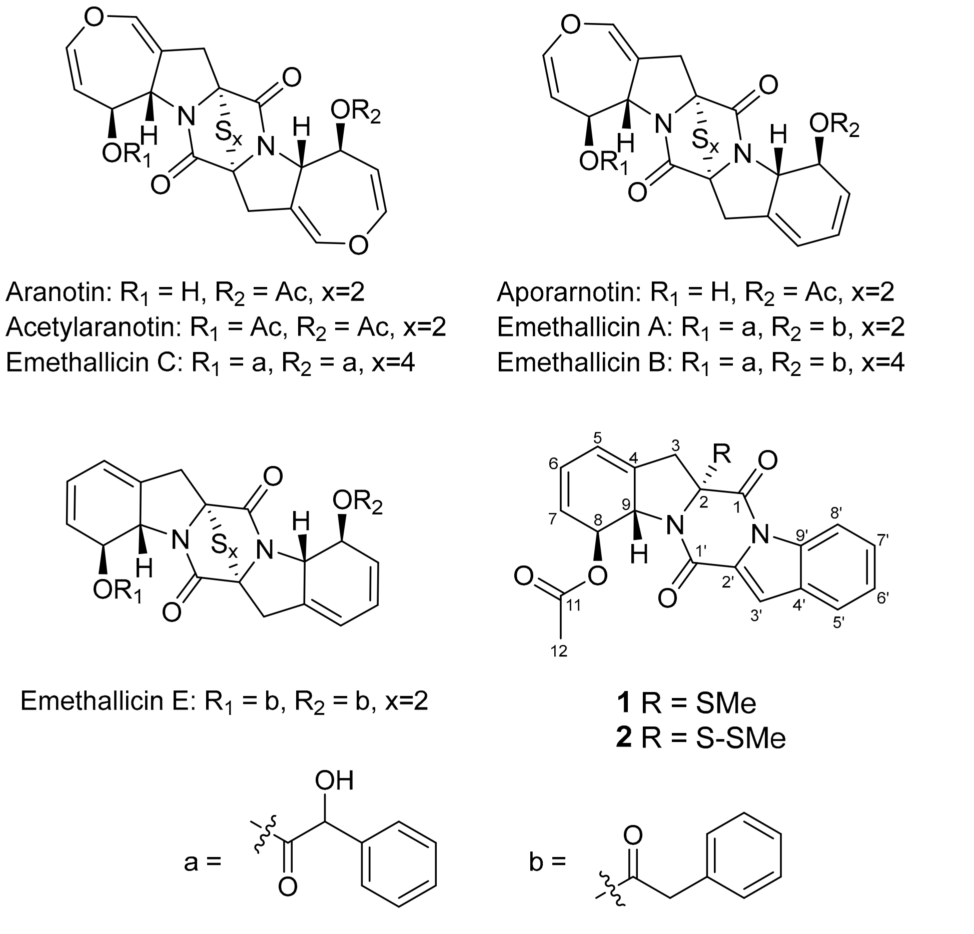

Figure S2. The structure of **1** and **2**, known aranotin-type ETPs and analogue.

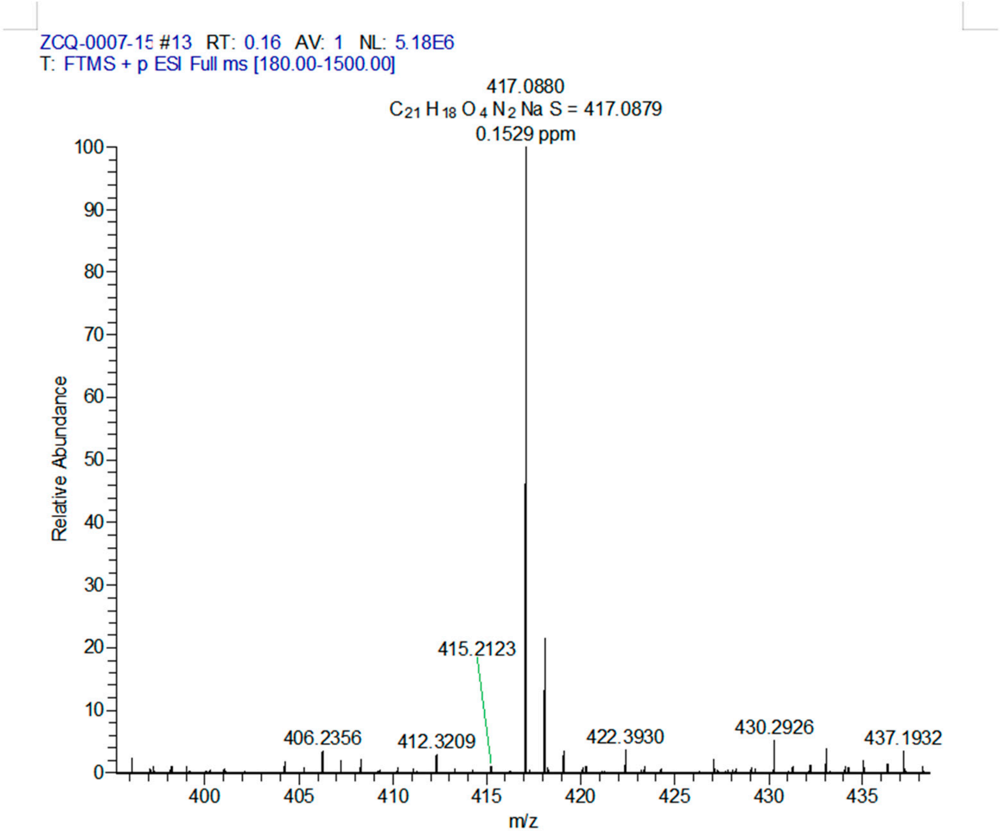

Figure S3. HRESIMS spectrum of corallomycetellain A (**1**).

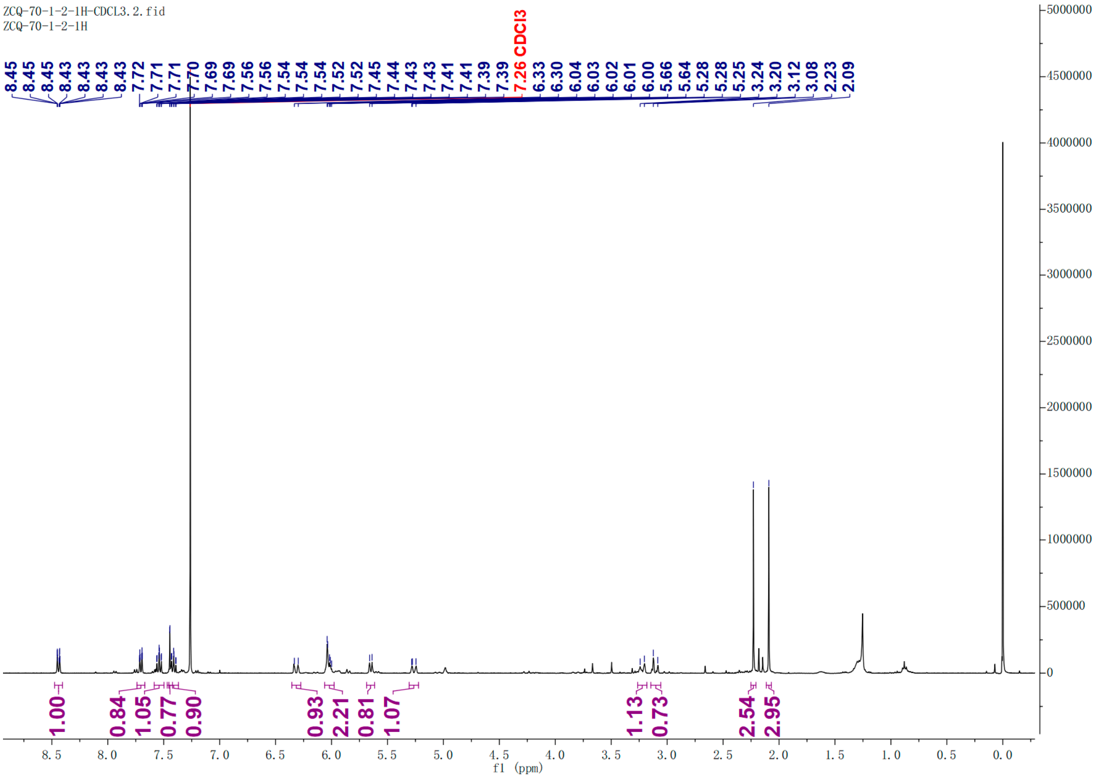

Figure S4. ^{1}H NMR (500 MHz, $CDCl_3$) spectrum of corallomycetellain A (**1**).

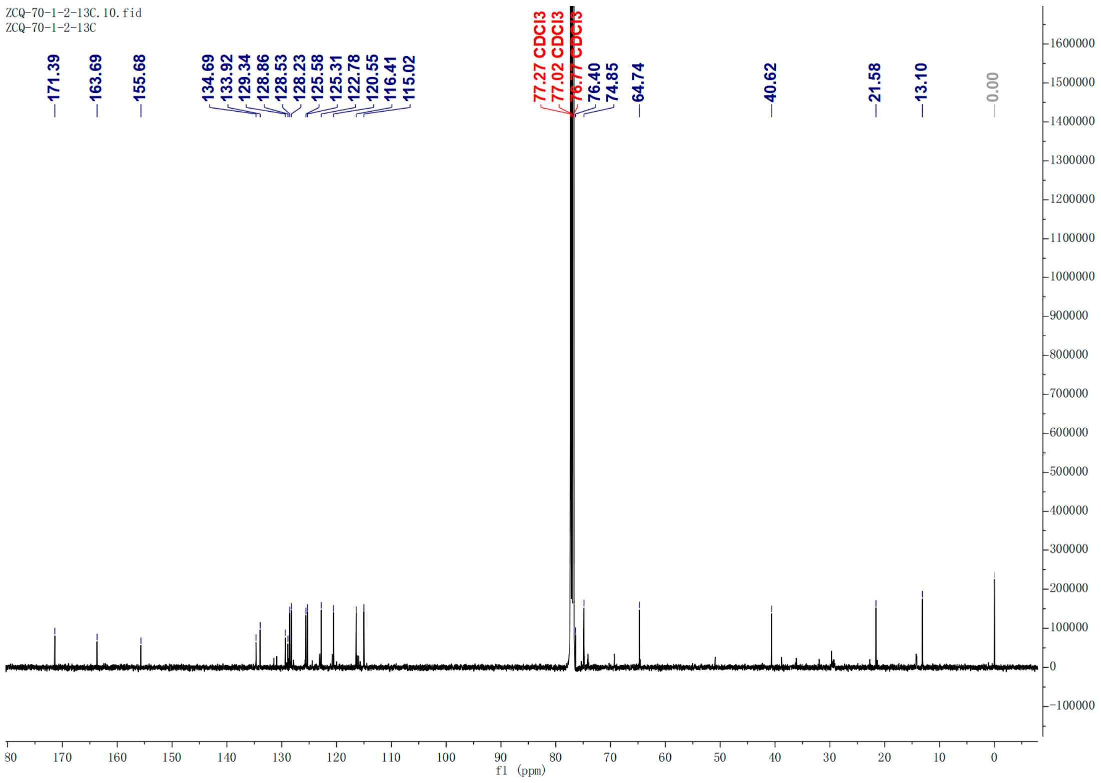

Figure S5. ^{13}C NMR (125 MHz, $CDCl_3$) spectrum of corallomycetellain A (**1**).

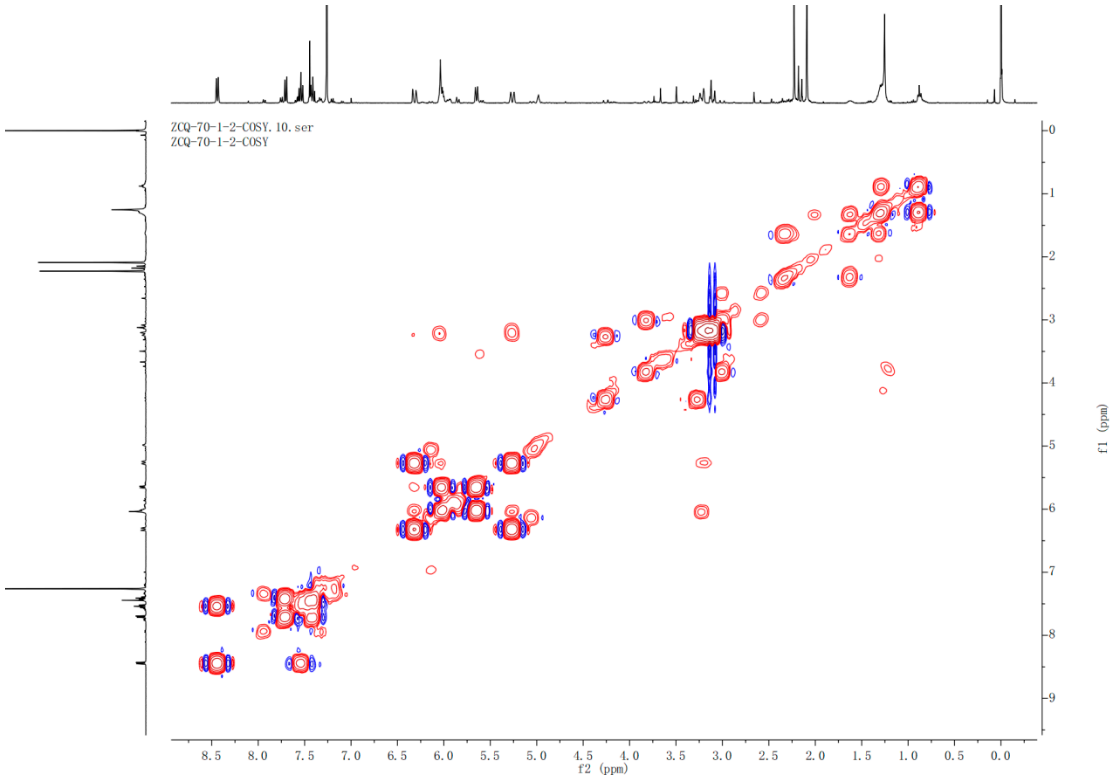

Figure S6. ^1H-^1H COSY spectrum of corallomycetellain A (**1**).

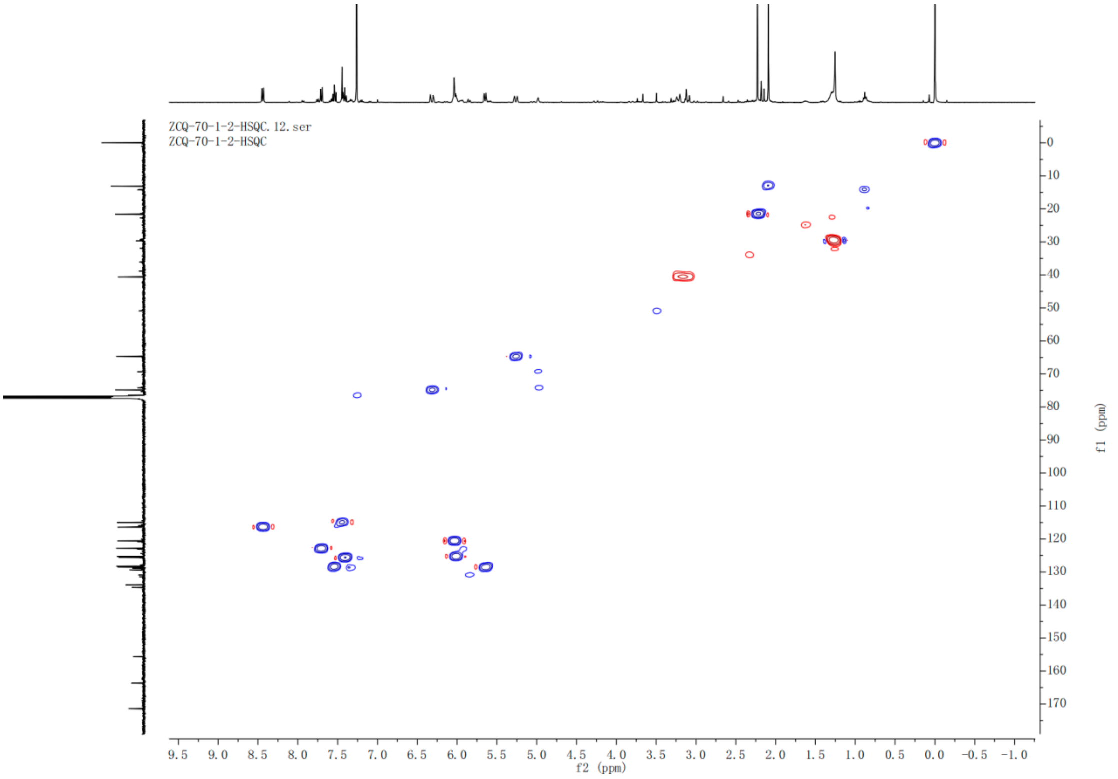

Figure S7. HSQC spectrum of corallomycetellain A (**1**).

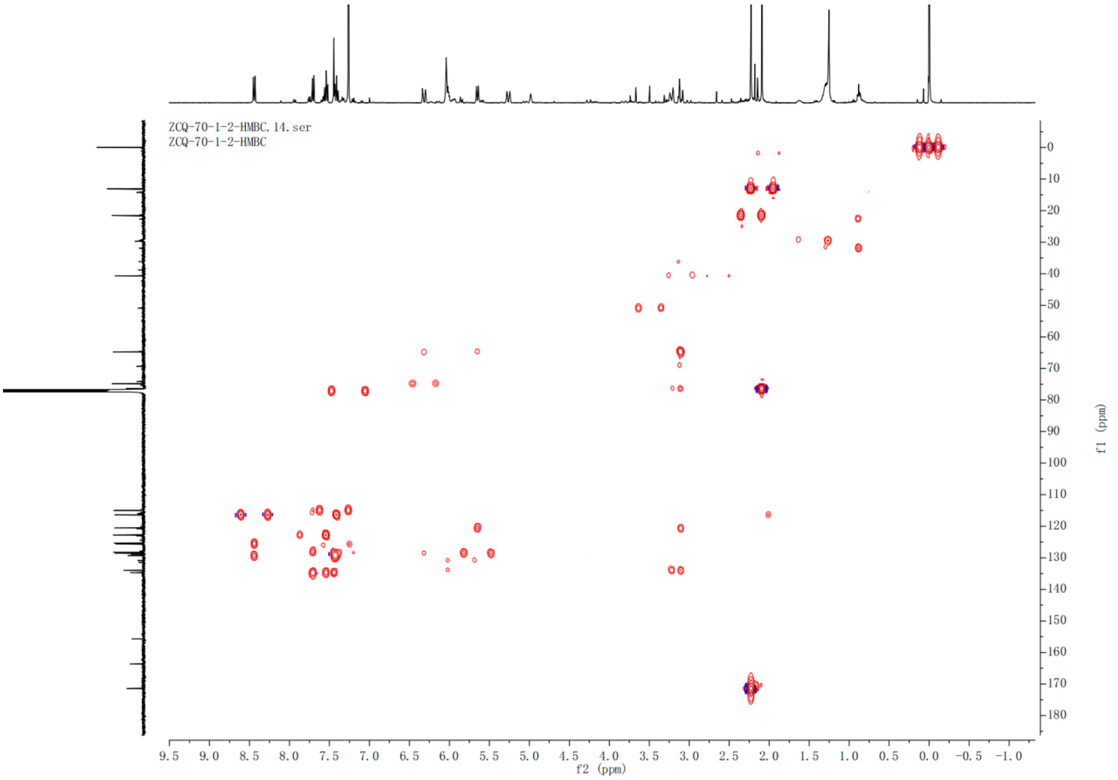

Figure S8. HMBC spectrum of corallomycetellain A (**1**).

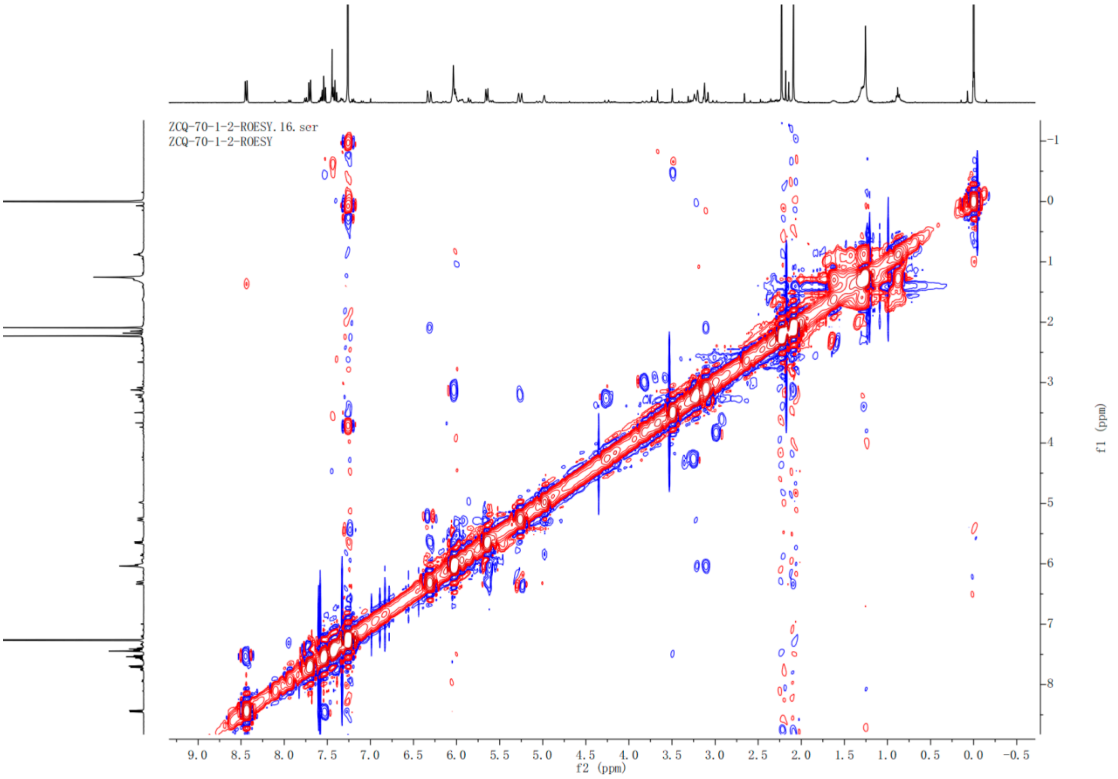

Figure S9. ROESY spectrum of corallomycetellain A (**1**).

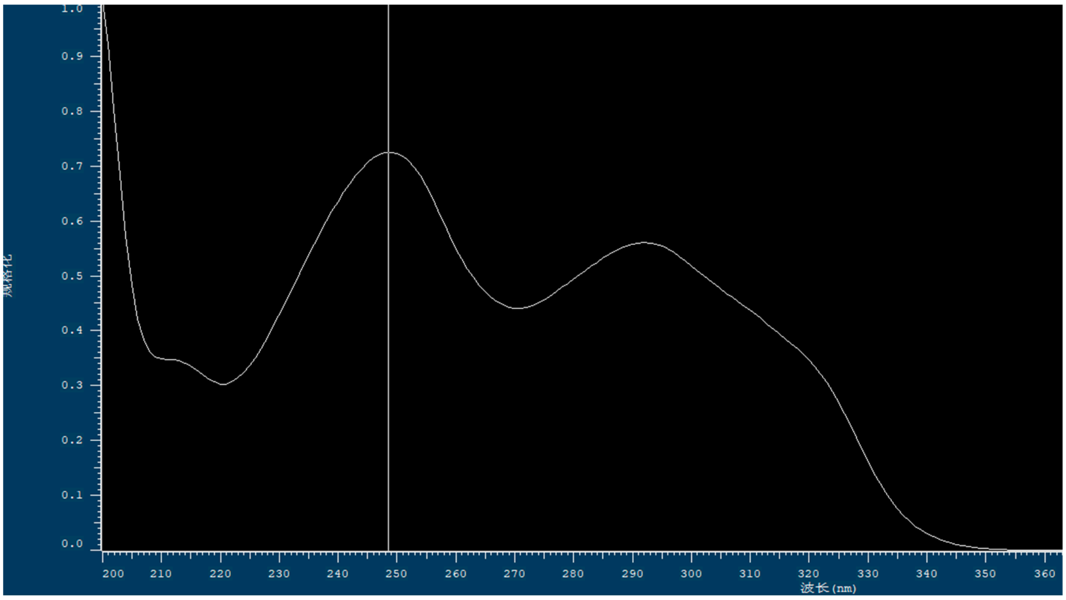

Figure S10. The UV spectrum of corallomycetellain A (**1**).

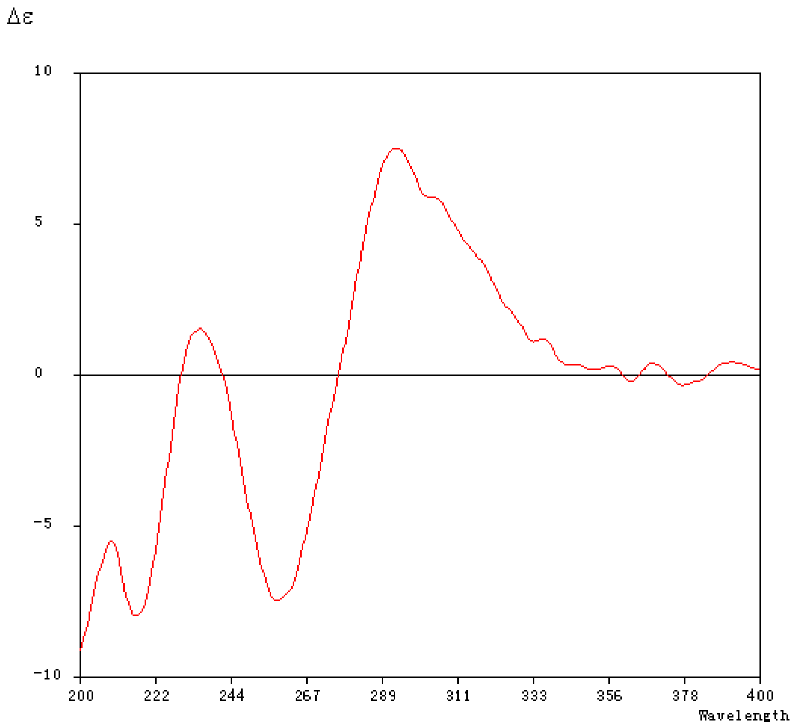

Figure S11. The CD spectrum of corallomycetellain A (**1**).

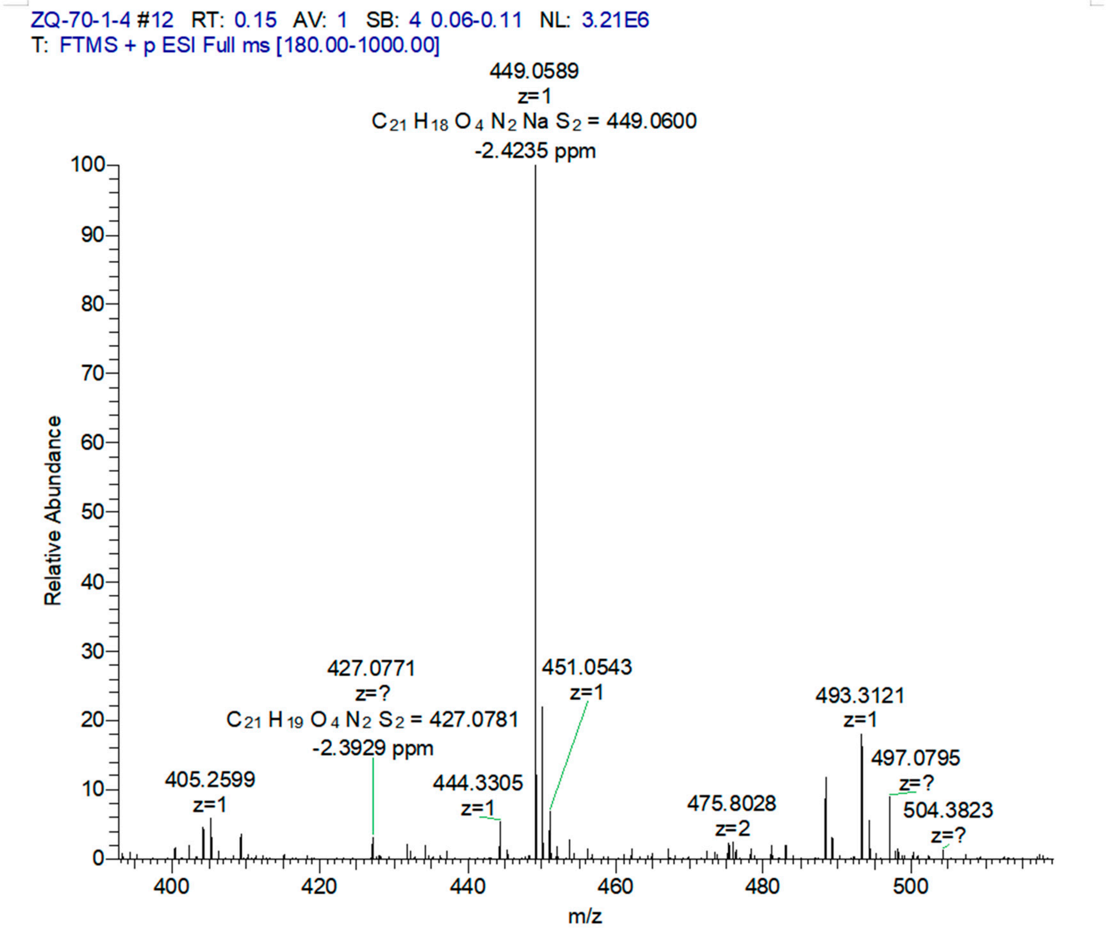

Figure S12. The HRESIMS spectrum of corallomycetellain B (**2**).

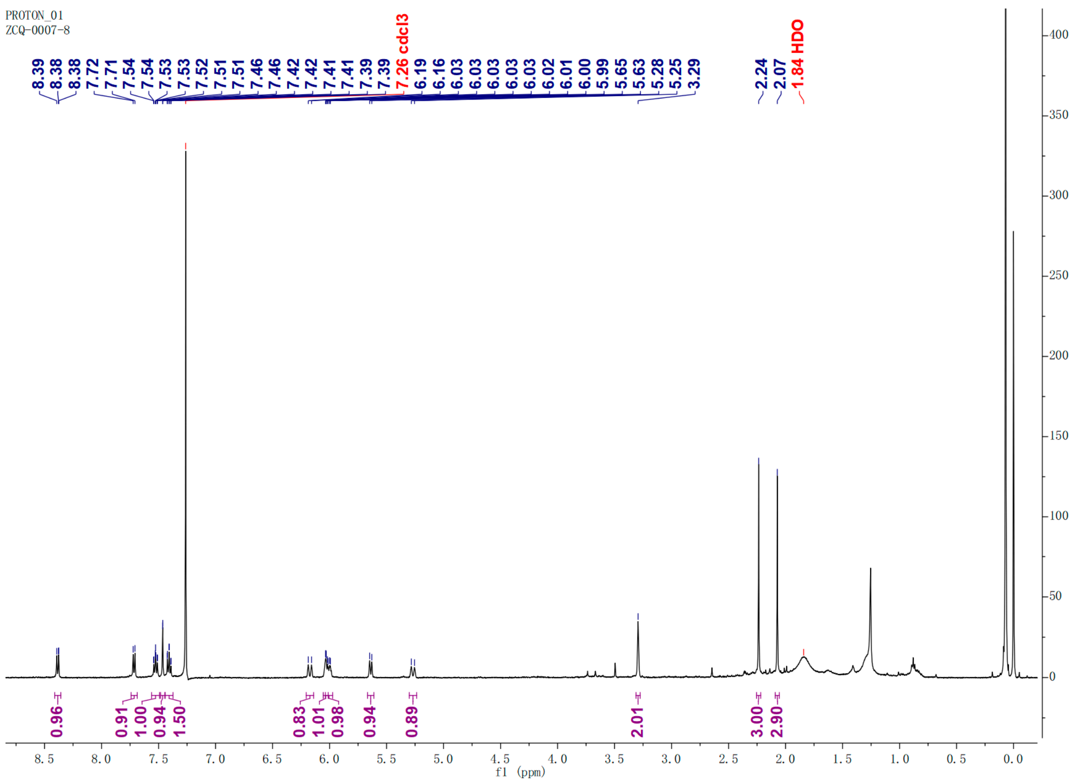

Figure S13. ^{1}H NMR (500 MHz, $CDCl_3$) spectrum of corallomycetellain B (**2**).

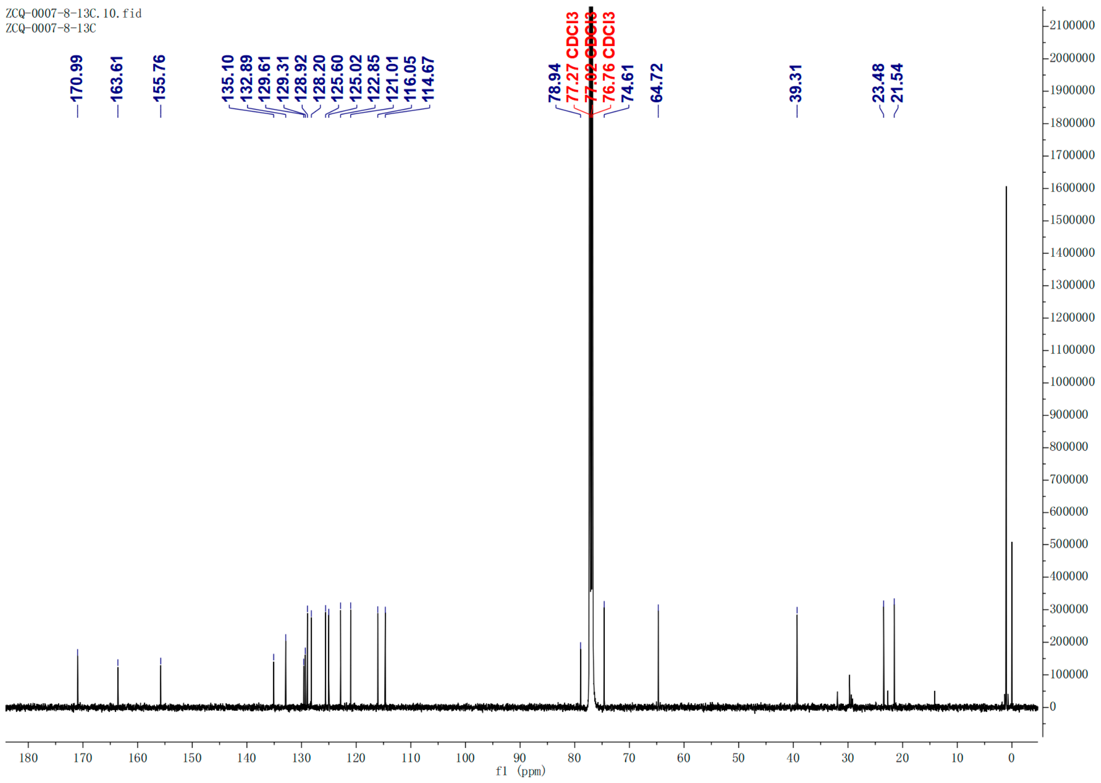

Figure S14. ^{13}C NMR (125 MHz, $CDCl_3$) spectrum of corallomycetellain B (**2**).

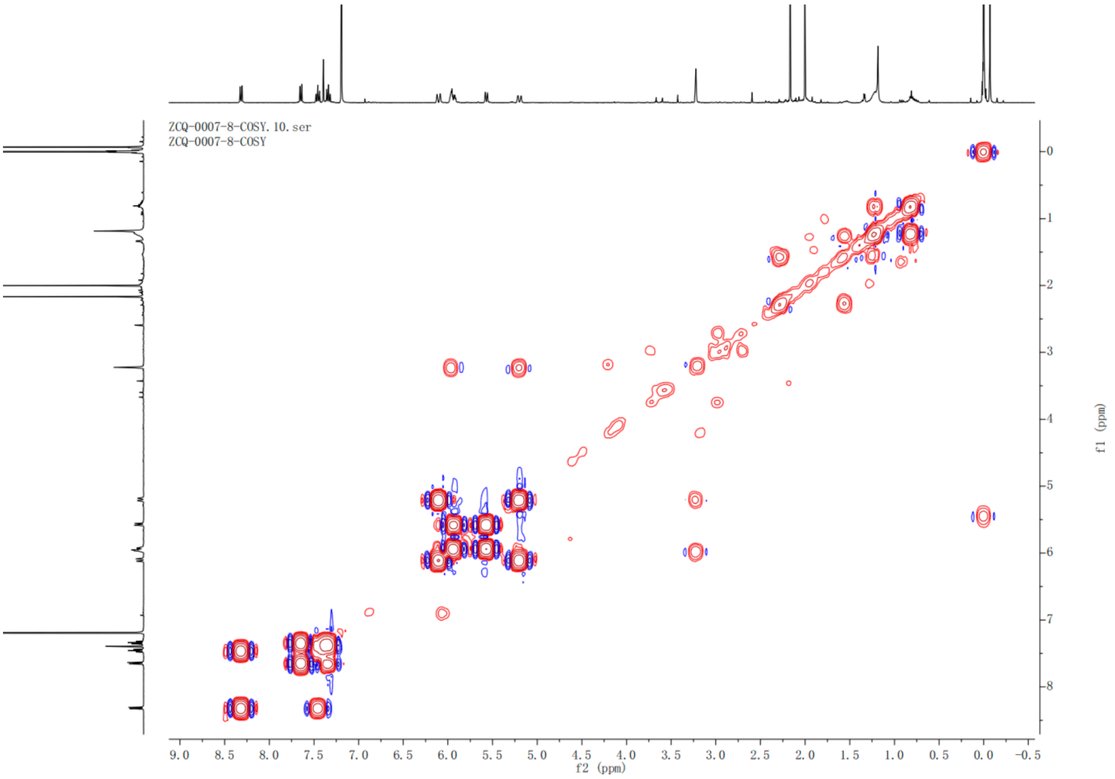

Figure S15. ^1H-^1H COSY spectrum of corallomycetellain B (**2**).

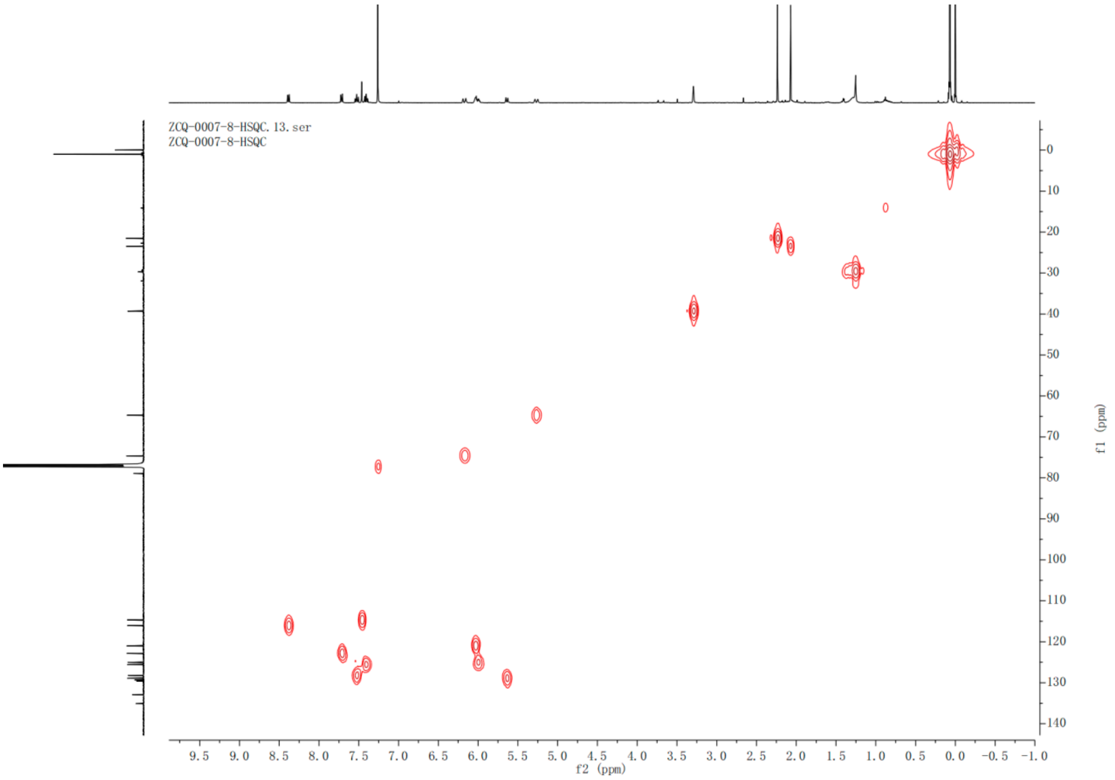

Figure S16. HSQC spectrum of corallomycetellain B (**2**).

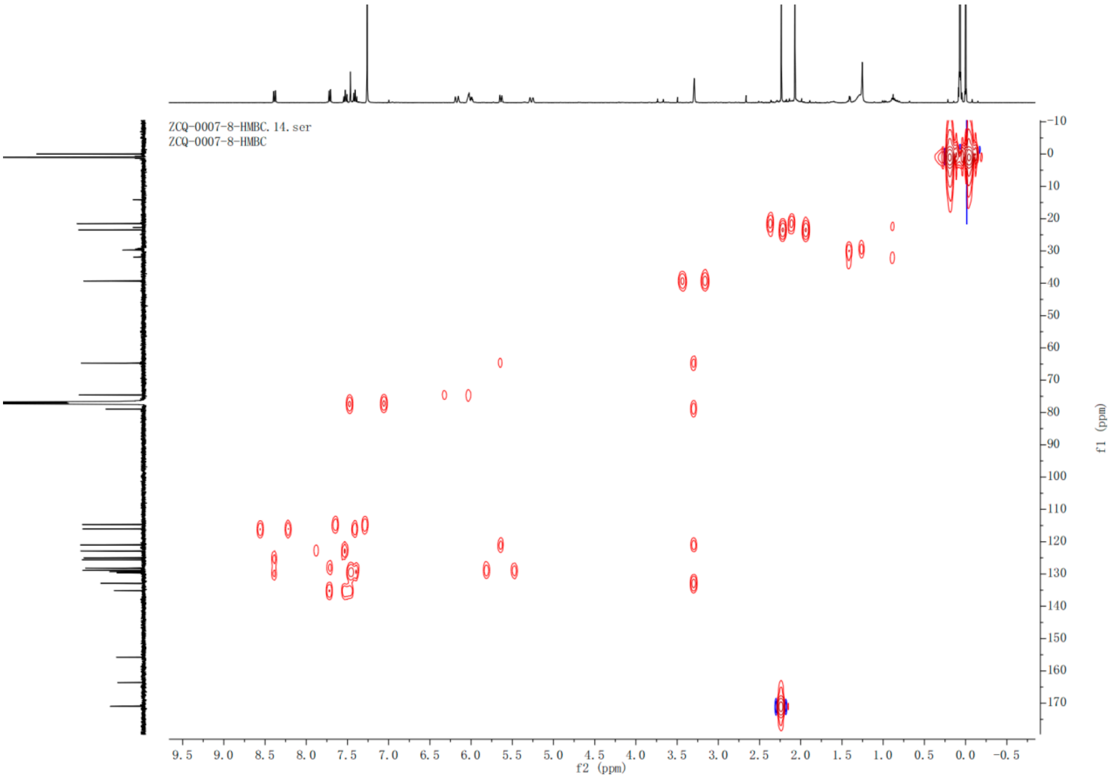

Figure S17. HMBC spectrum of corallomycetellain B (**2**).

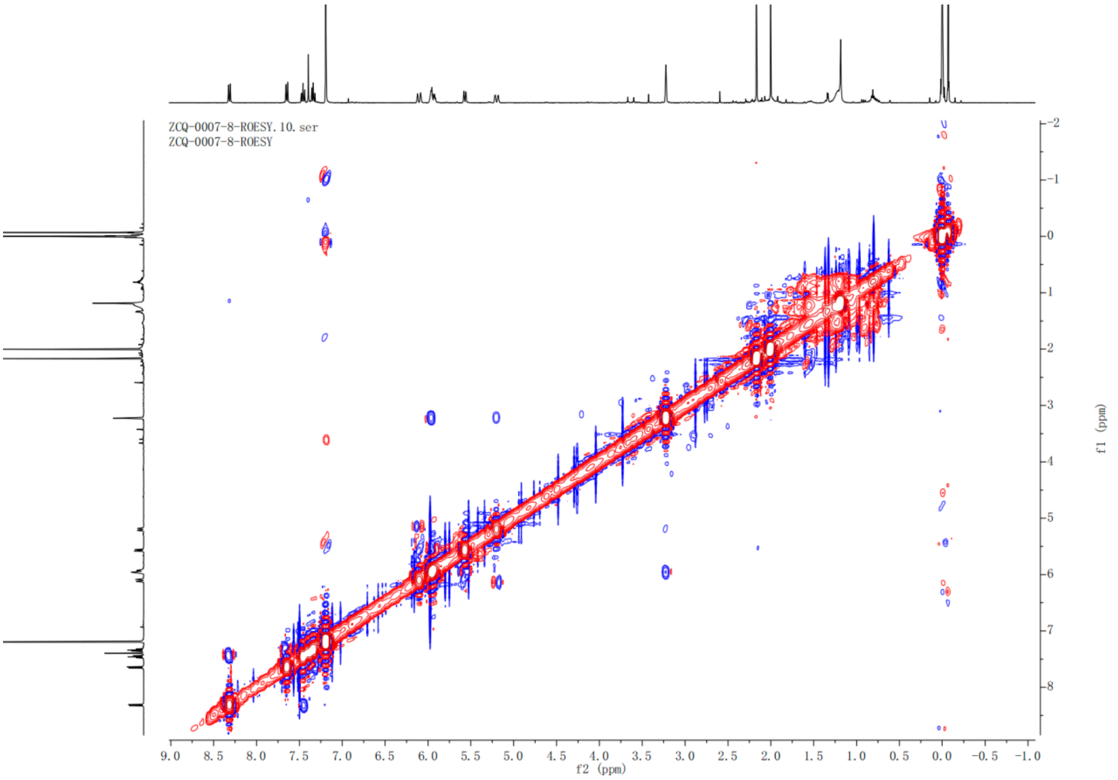

Figure S18. ROESY spectrum of corallomycetellain B (**2**).

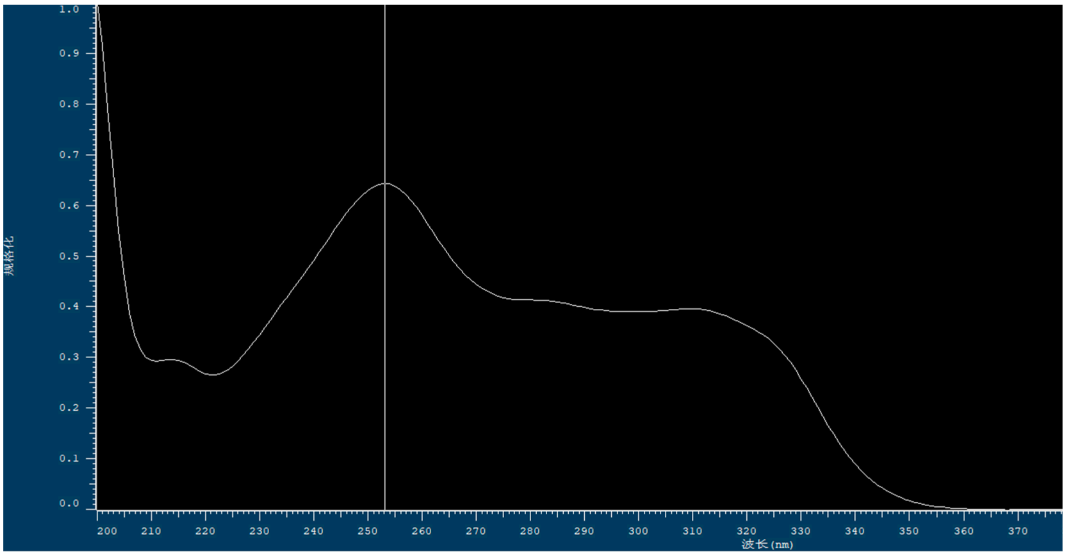

Figure S19. The UV spectrum of corallomycetellain B (**2**).

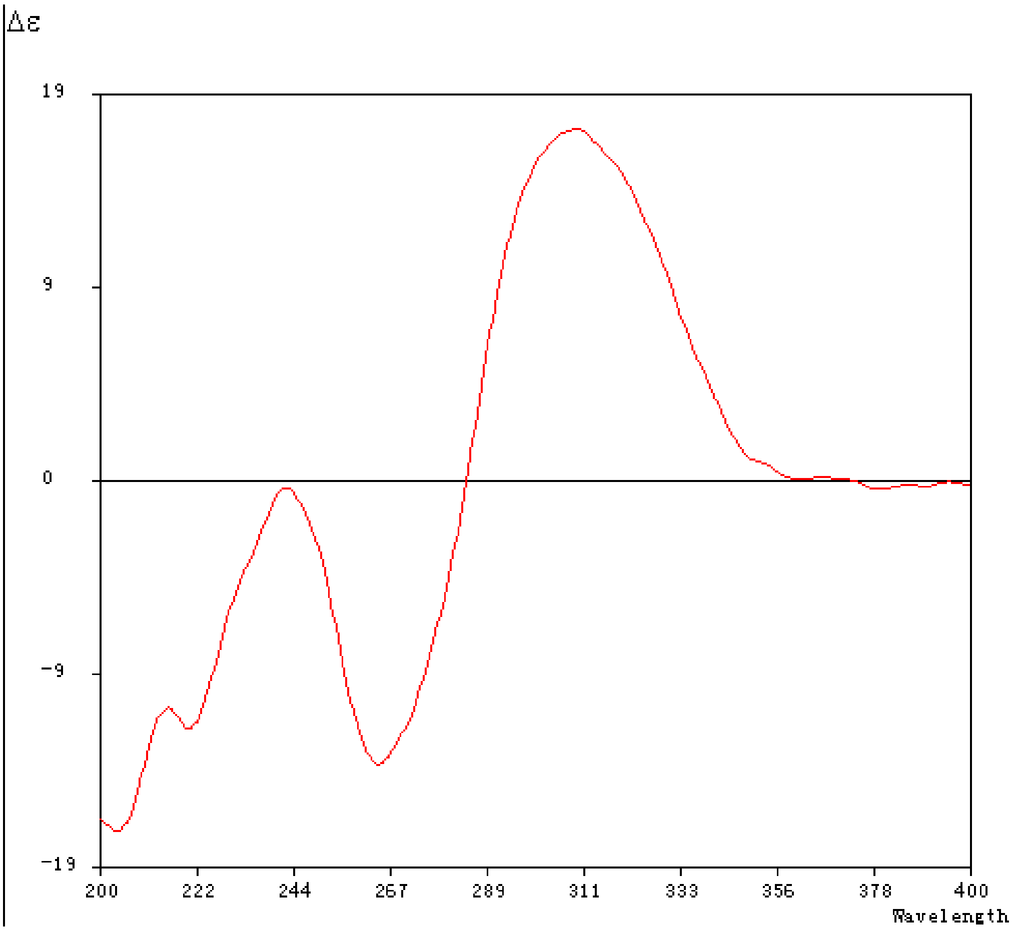

Figure S20. The CD spectrum of corallomycetellain B (**2**).

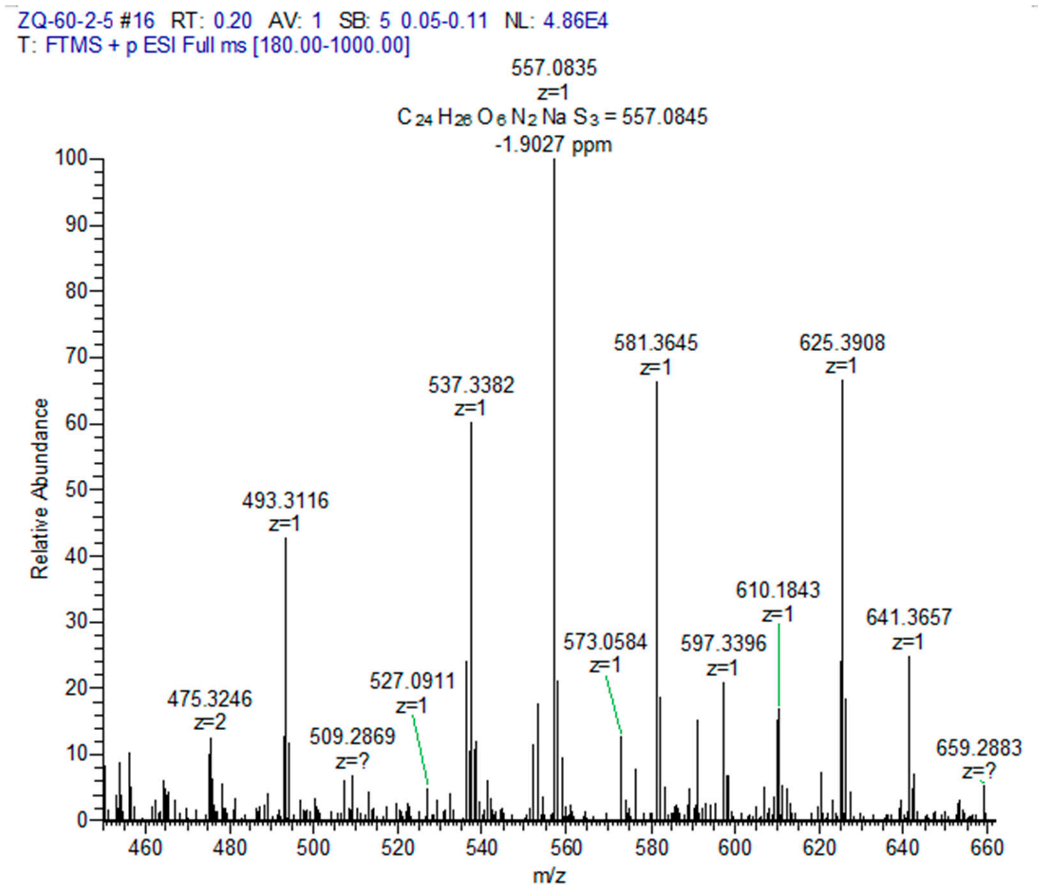

Figure S21. The HRESIMS spectrum of corallomycetellain C (**3**).

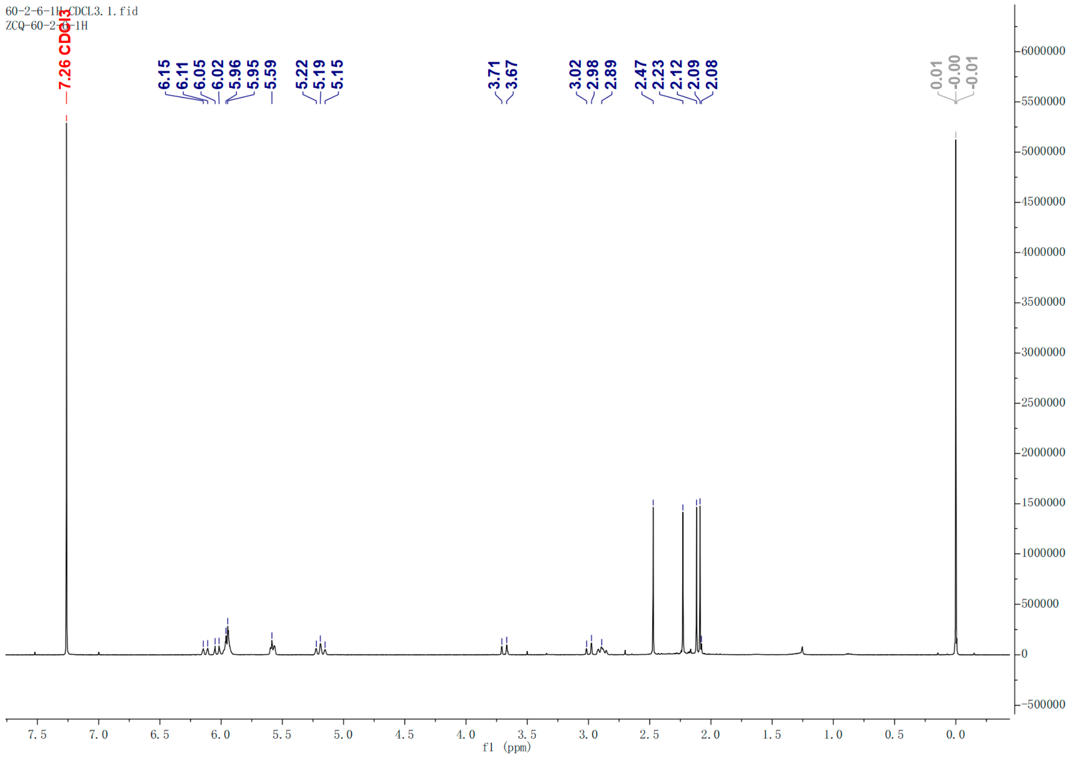

Figure S22. ^{1}H NMR (500 MHz, $CDCl_3$) spectrum of corallomycetellain C (**3**).

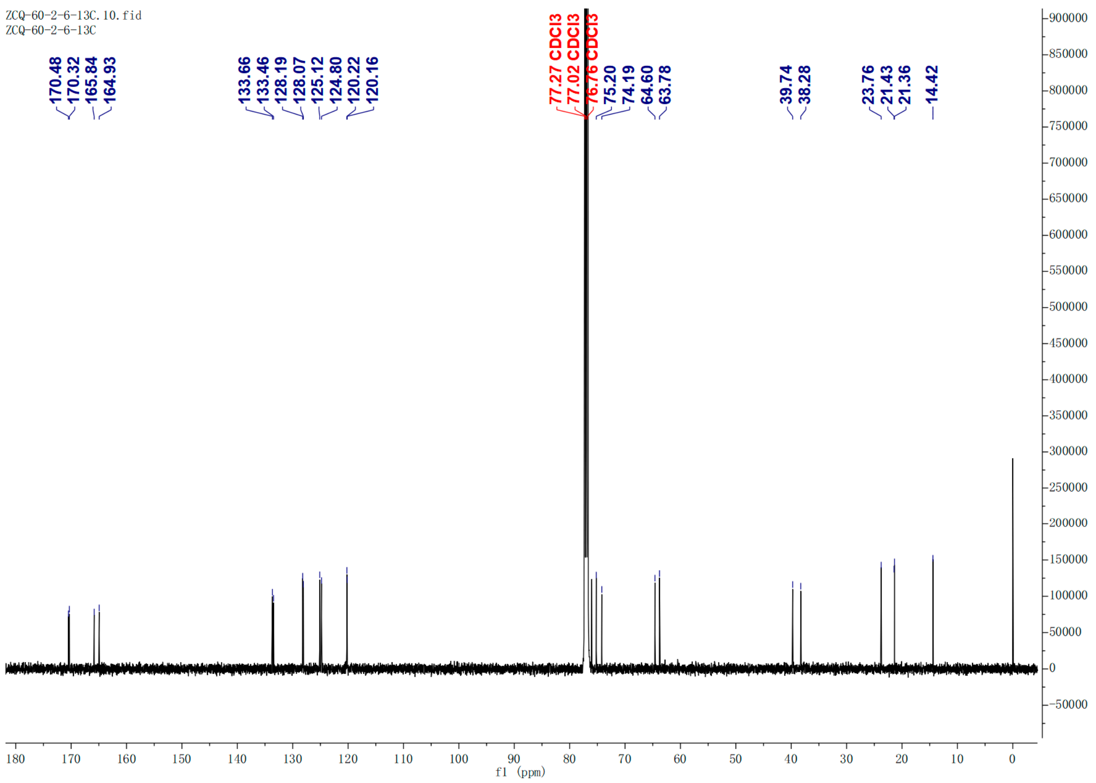

Figure S23. ^{13}C NMR (125 MHz, $CDCl_3$) spectrum of corallomycetellain C (**3**).

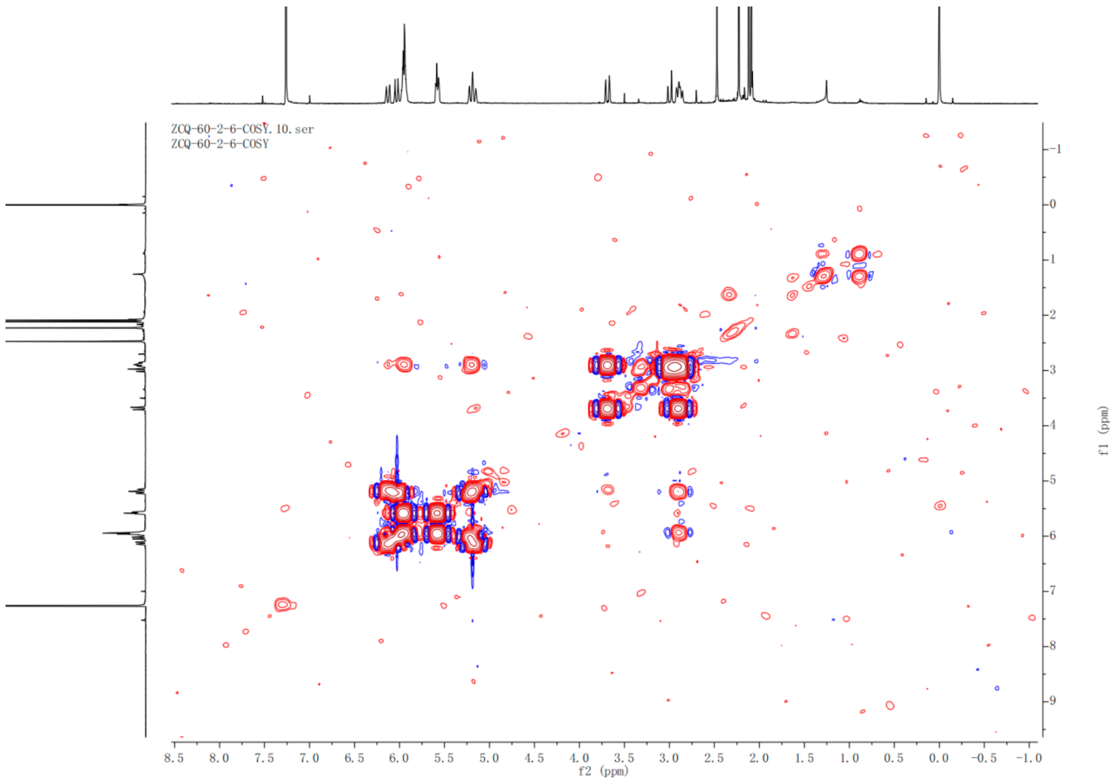

Figure S24. ^{1}H-^{1}H COSY spectrum of corallomycetellain C (**3**).

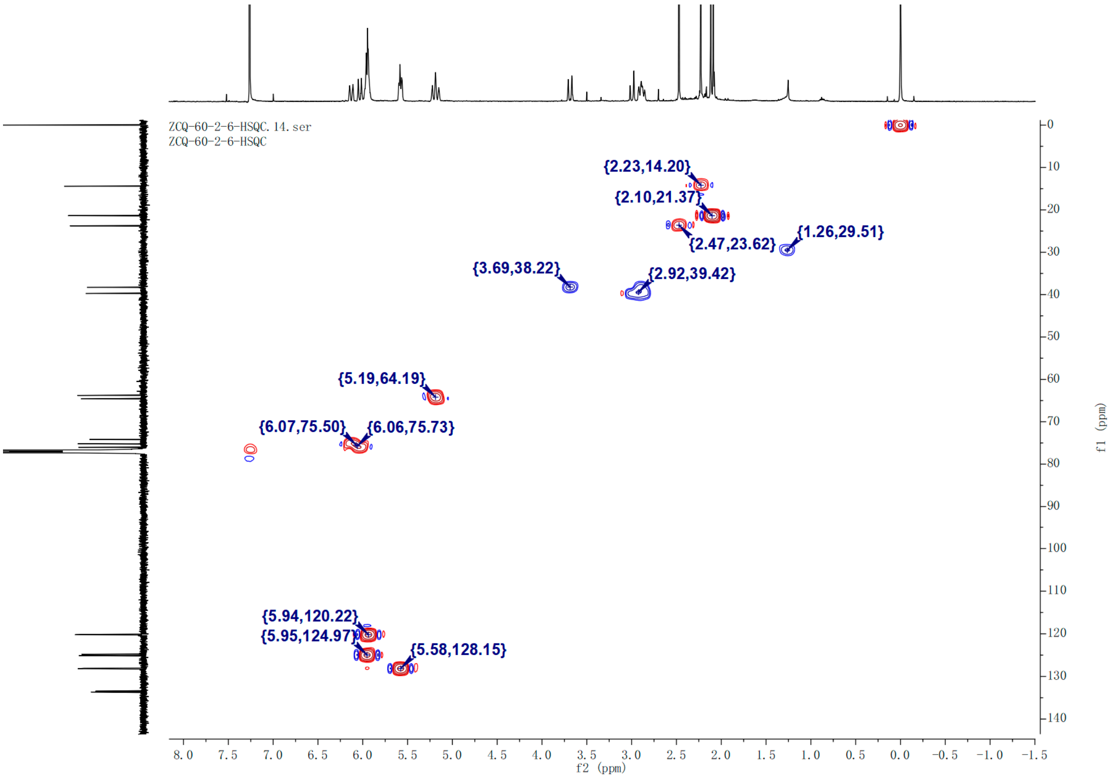

Figure S25. HSQC spectrum of corallomycetellain C (**3**).

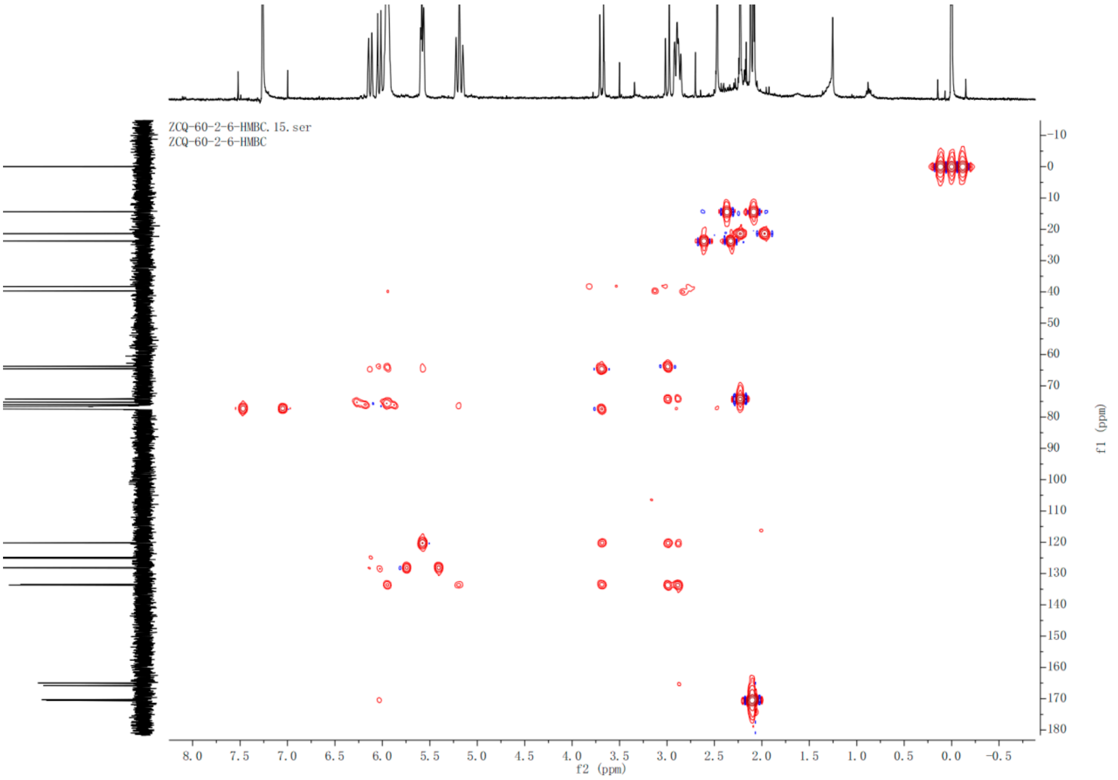

Figure S26. HMBC spectrum of corallomycetellain C (**3**).

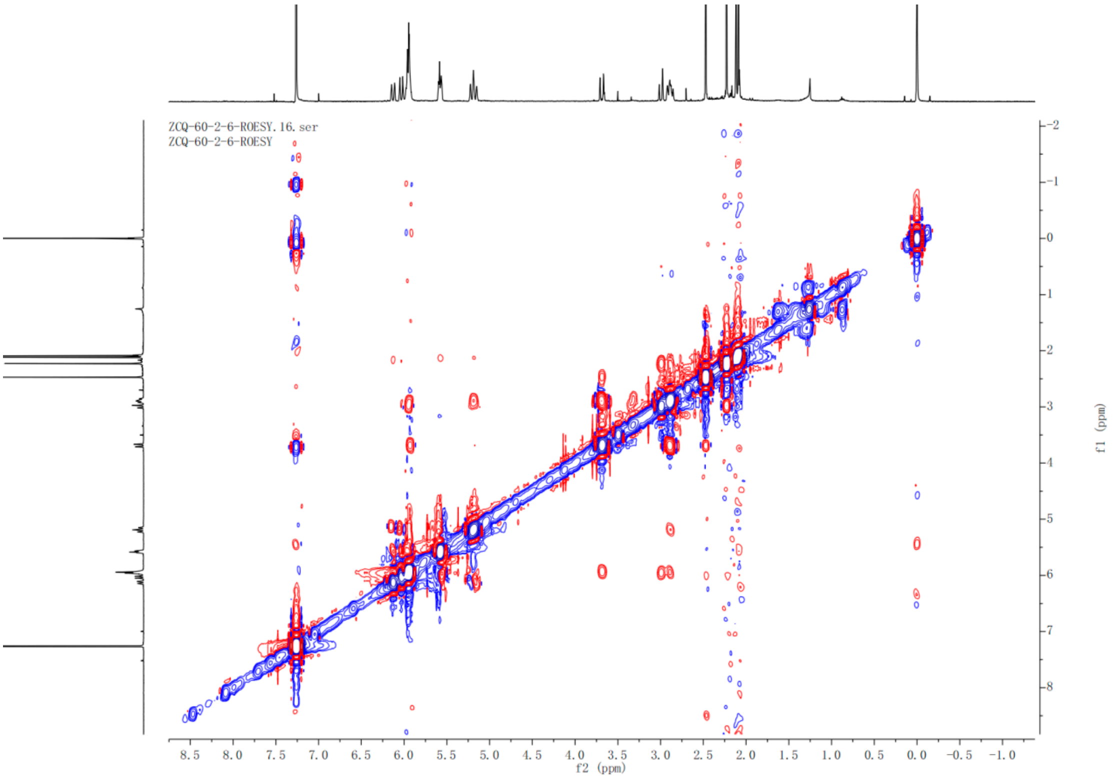

Figure S27. ROESY spectrum of corallomycetellain C (**3**).

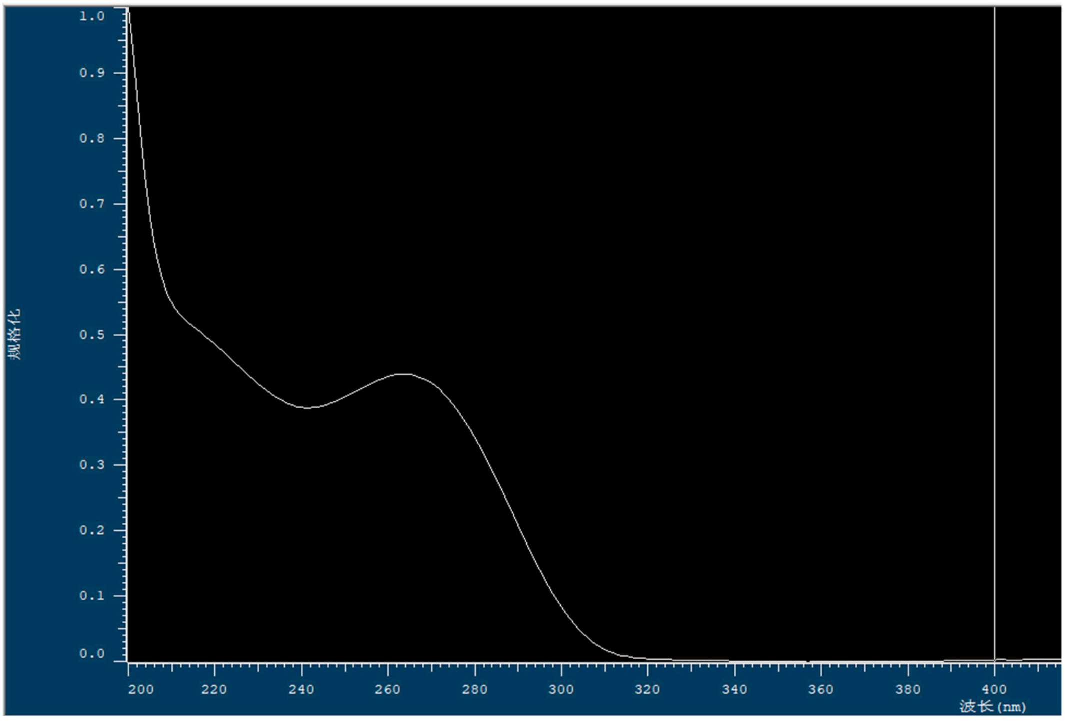

Figure S28. The UV spectrum of corallomycetellain C (**3**).

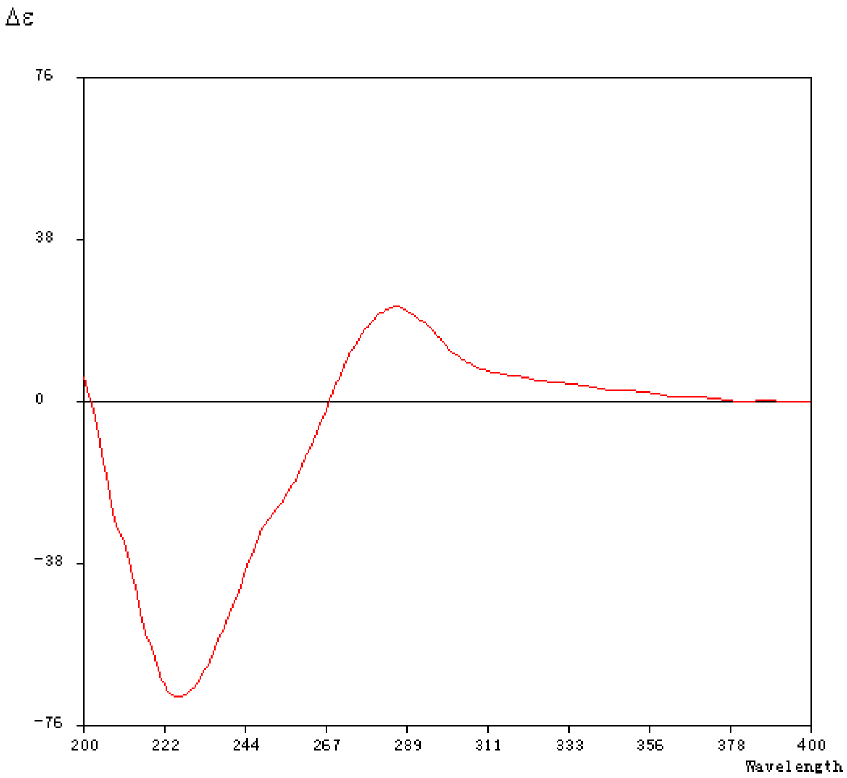

Figure S29. The CD spectrum of corallomycetellain C (**3**).

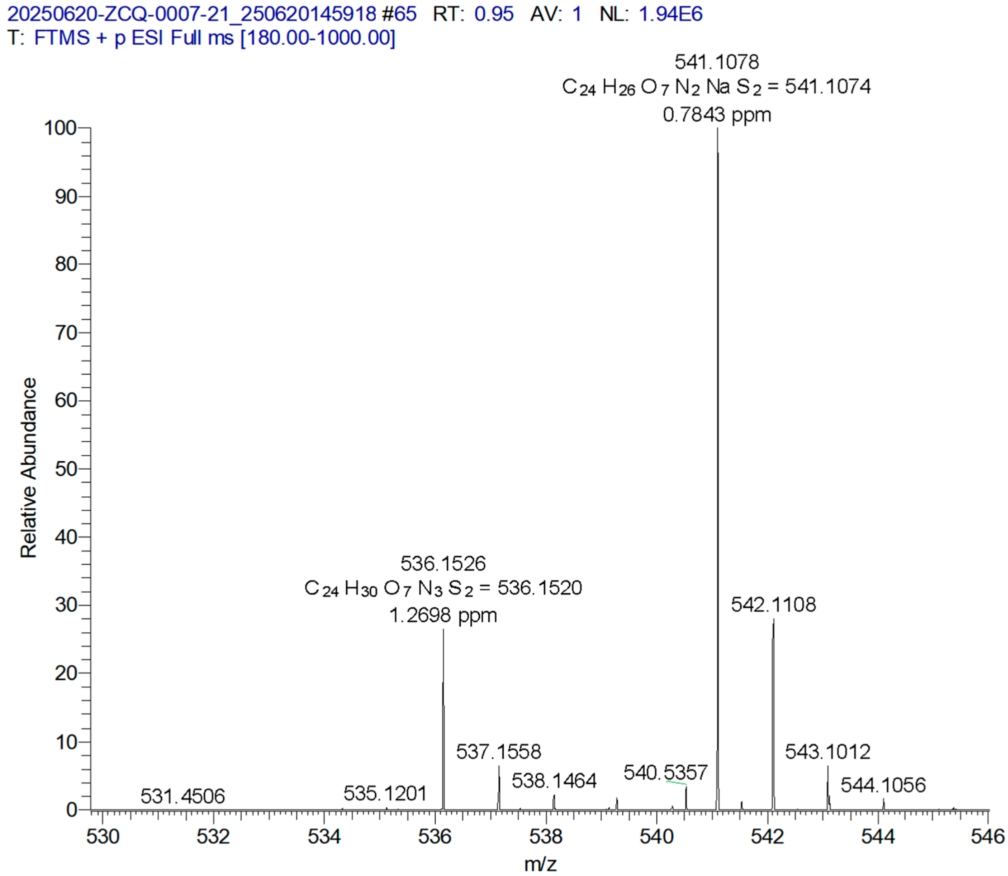

Figure S30. The HRESIMS spectrum of corallomycetellain D (**4**).

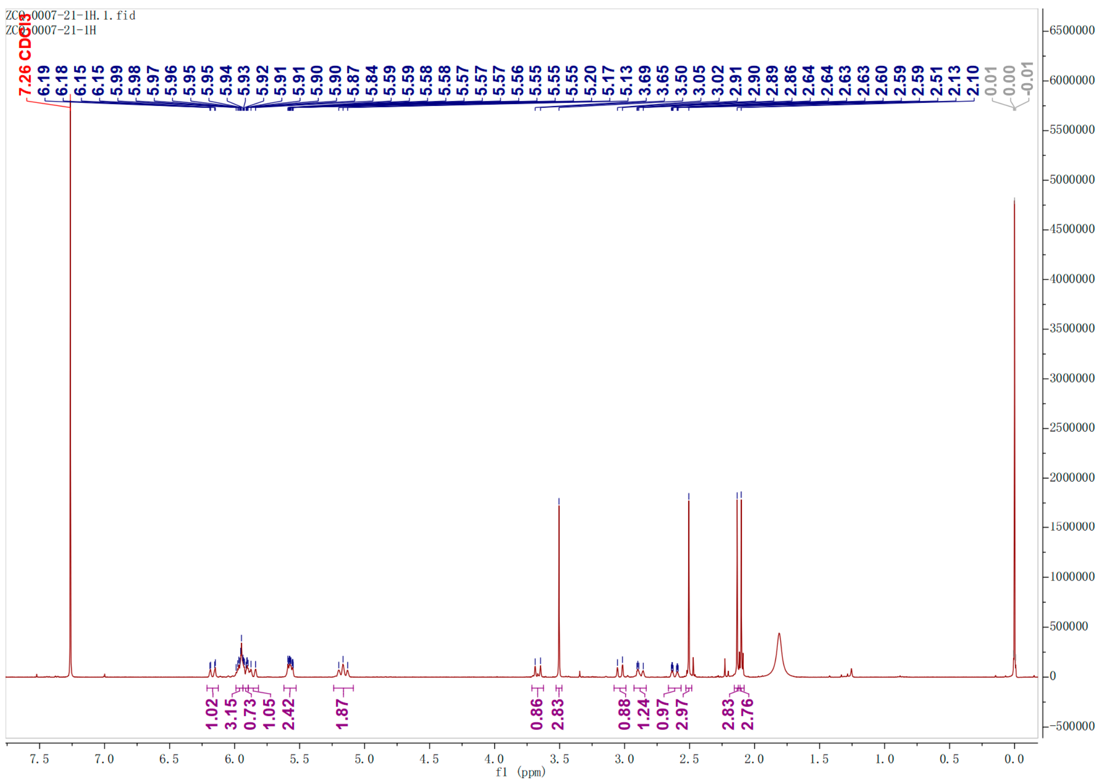

Figure S31. ^{1}H NMR (600 MHz, $CDCl_3$) spectrum of corallomycetellain D (**4**).

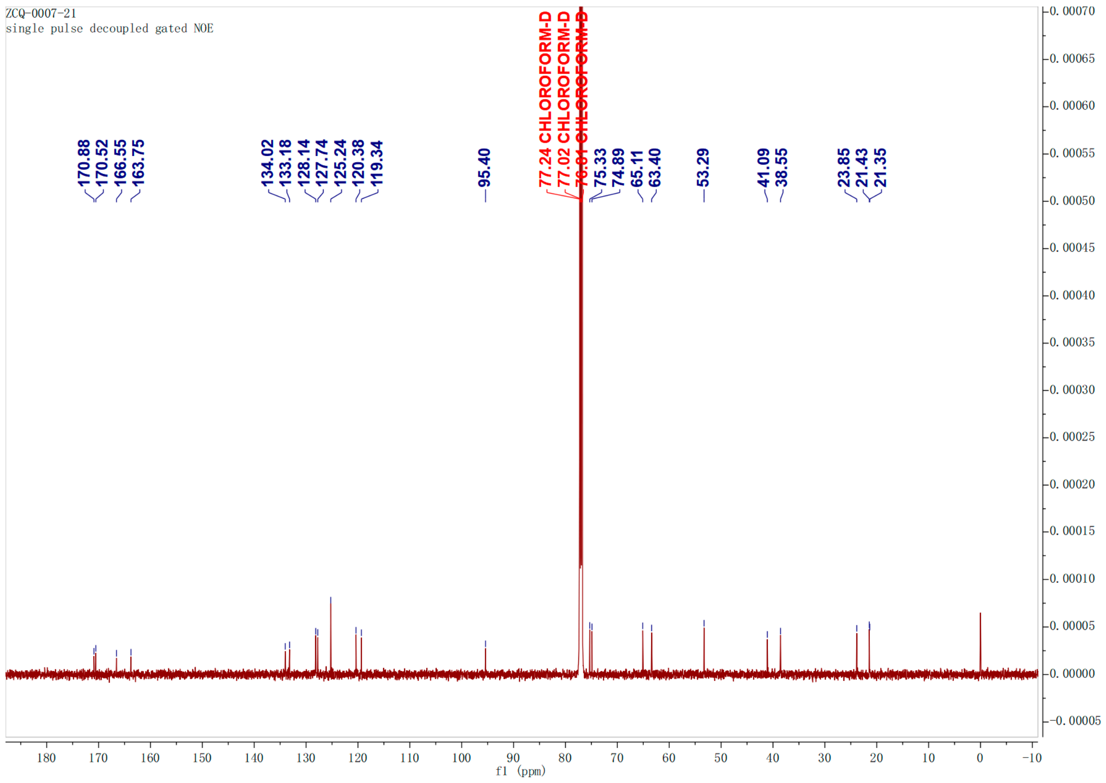

Figure S32. ^{13}C NMR (150 MHz, $CDCl_3$) spectrum of corallomycetellain D (**4**).

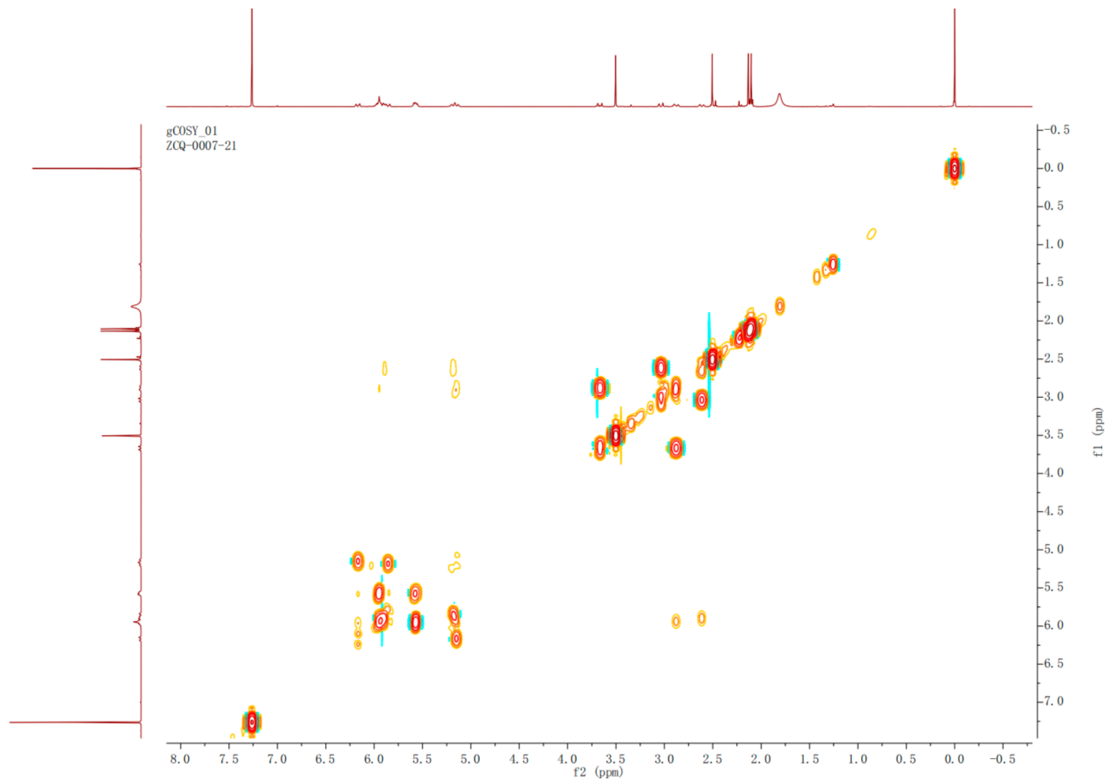

Figure S33. ^{1}H-^{1}H COSY spectrum of corallomycetellain D (**4**).

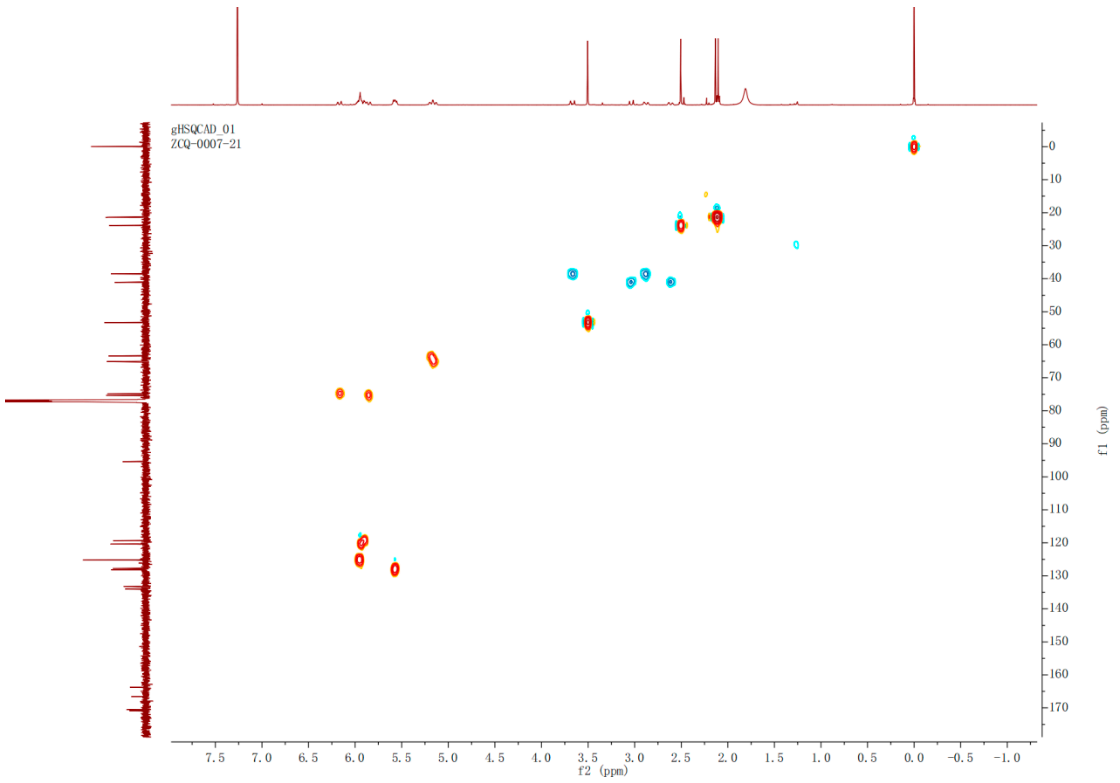

Figure S34. HSQC spectrum of corallomycetellain D (**4**).

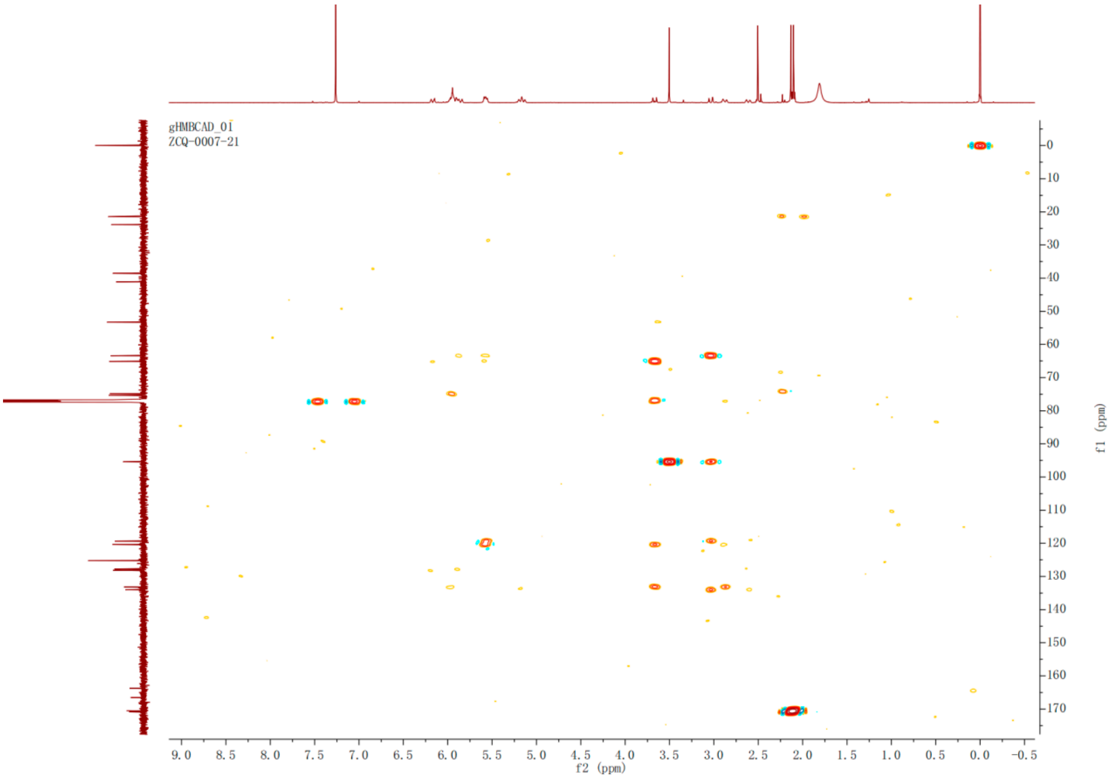

Figure S35. HMBC spectrum of corallomycetellain D (**4**).

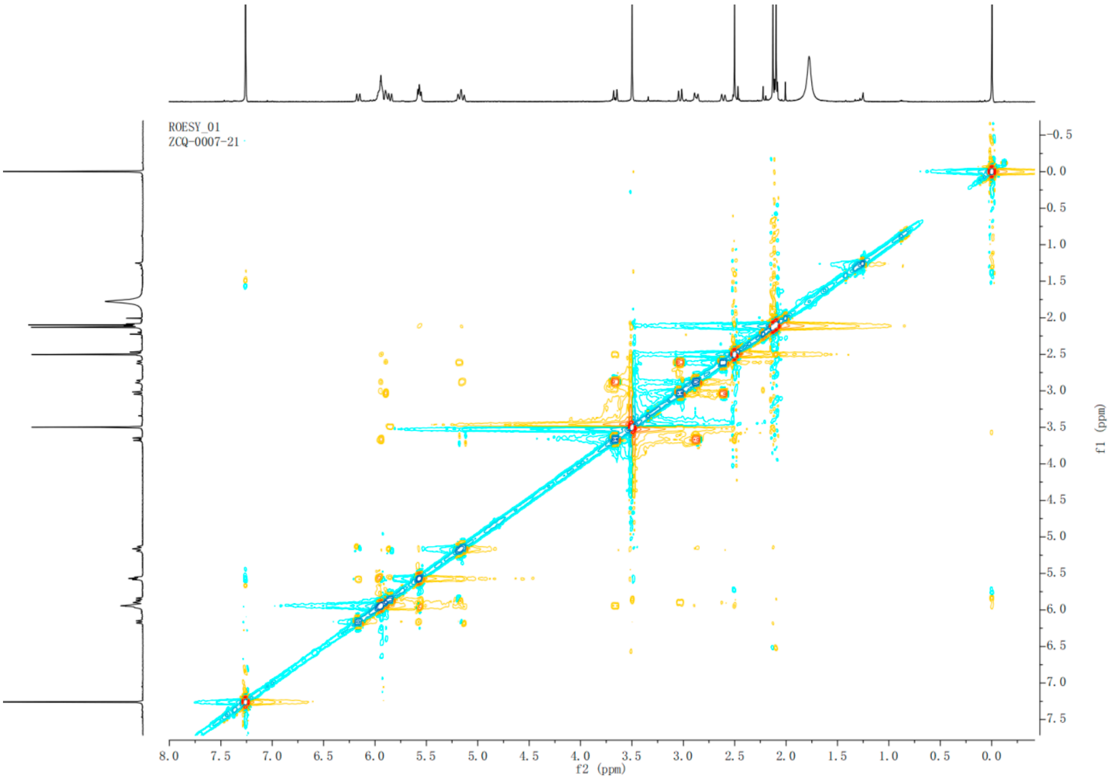

Figure S36. ROESY spectrum of corallomycetellain D (**4**).

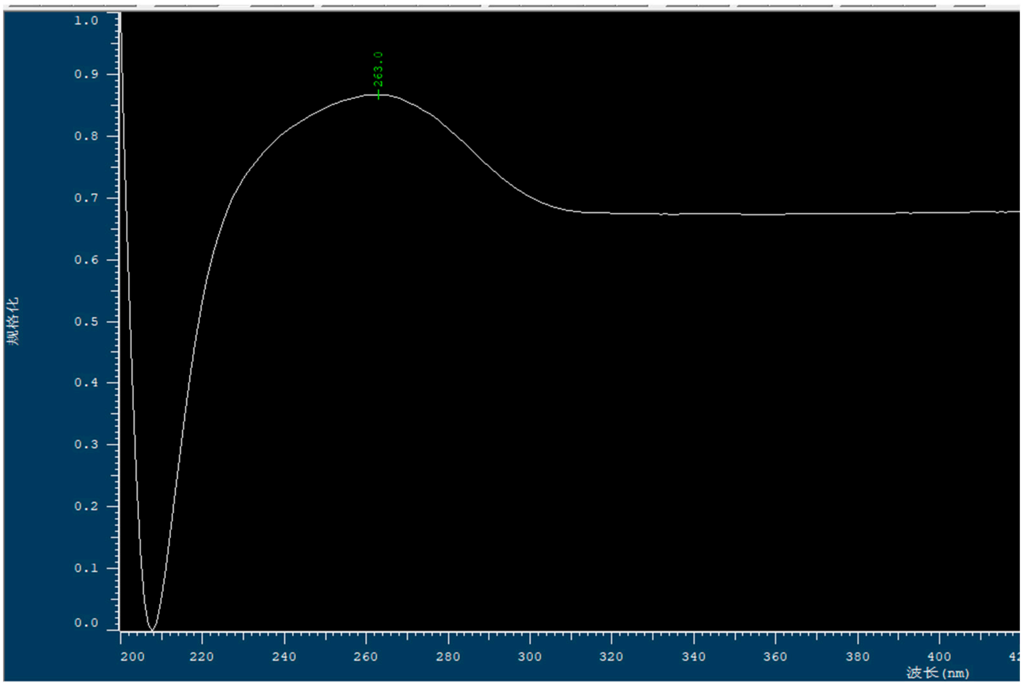

Figure S37. The UV spectrum of corallomycetellain D (**4**).

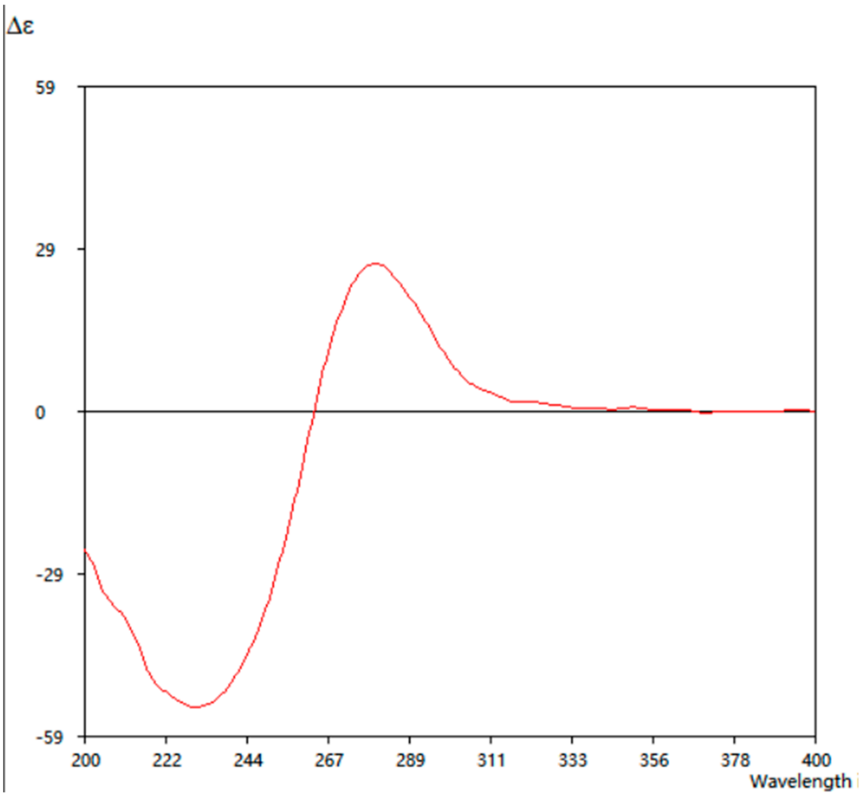

Figure S38. The CD spectrum of corallomycetellain D (**4**).

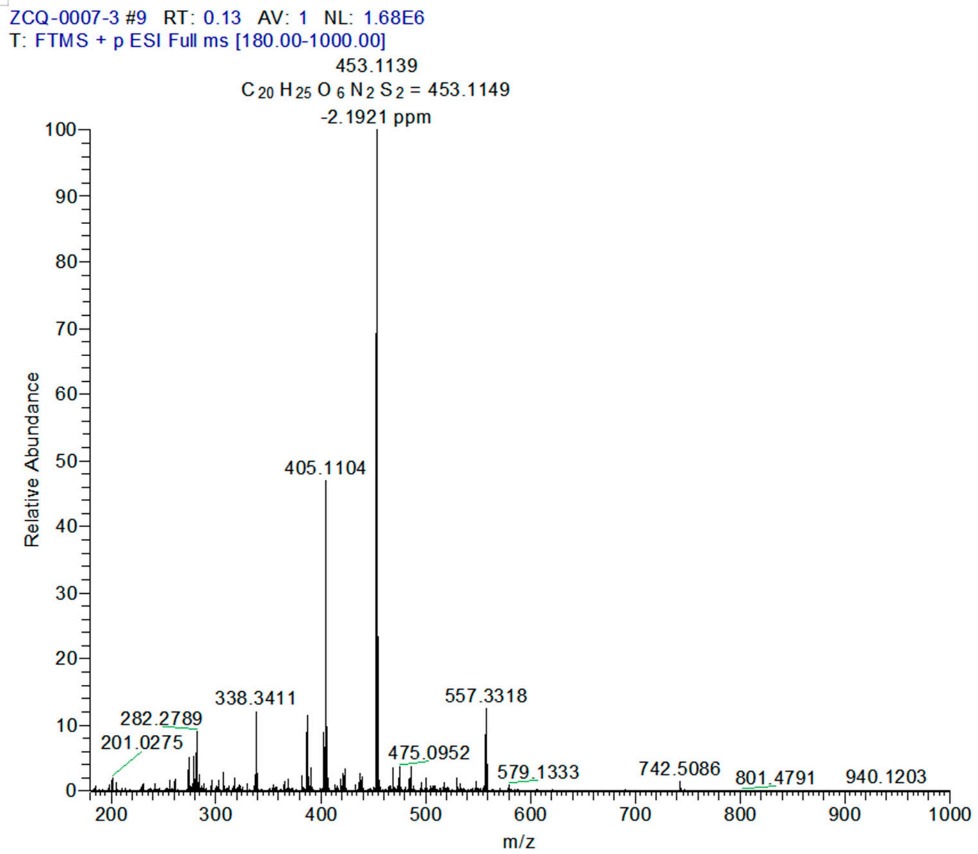

Figure S39. The HRESIMS spectrum of corallomycetellain E (**5**).

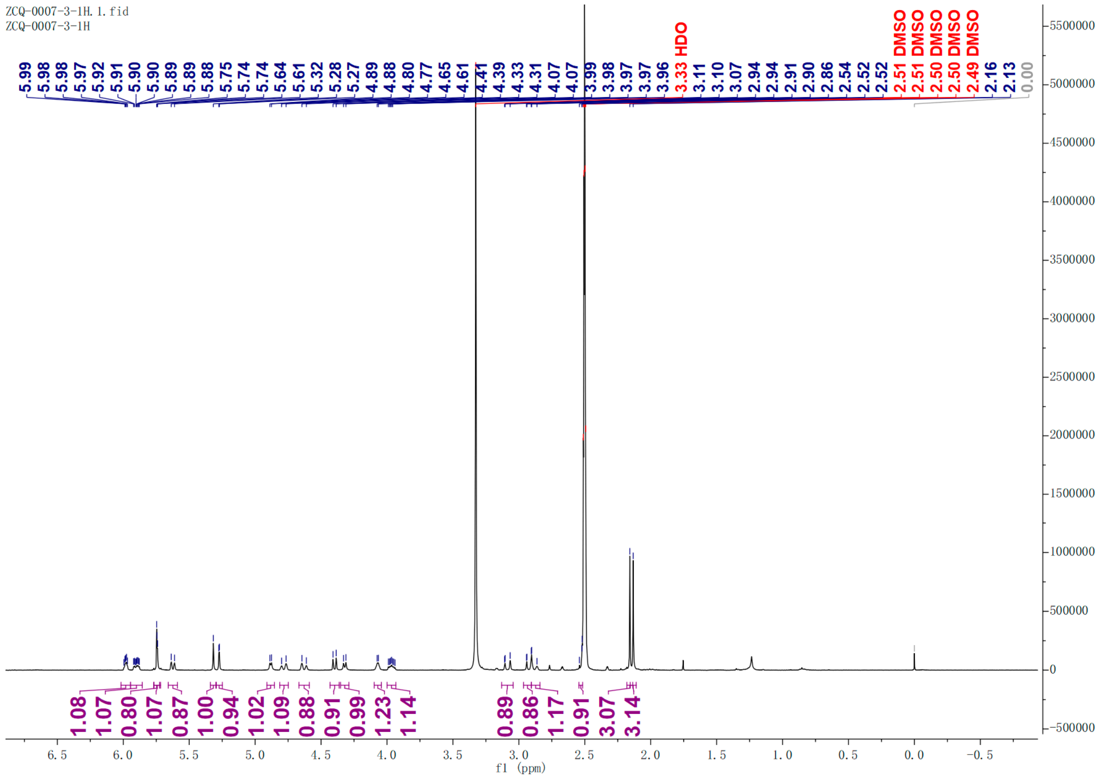

Figure S40. ^{1}H NMR (500 MHz, DMSO-d_6) spectrum of corallomycetellain E (**5**).

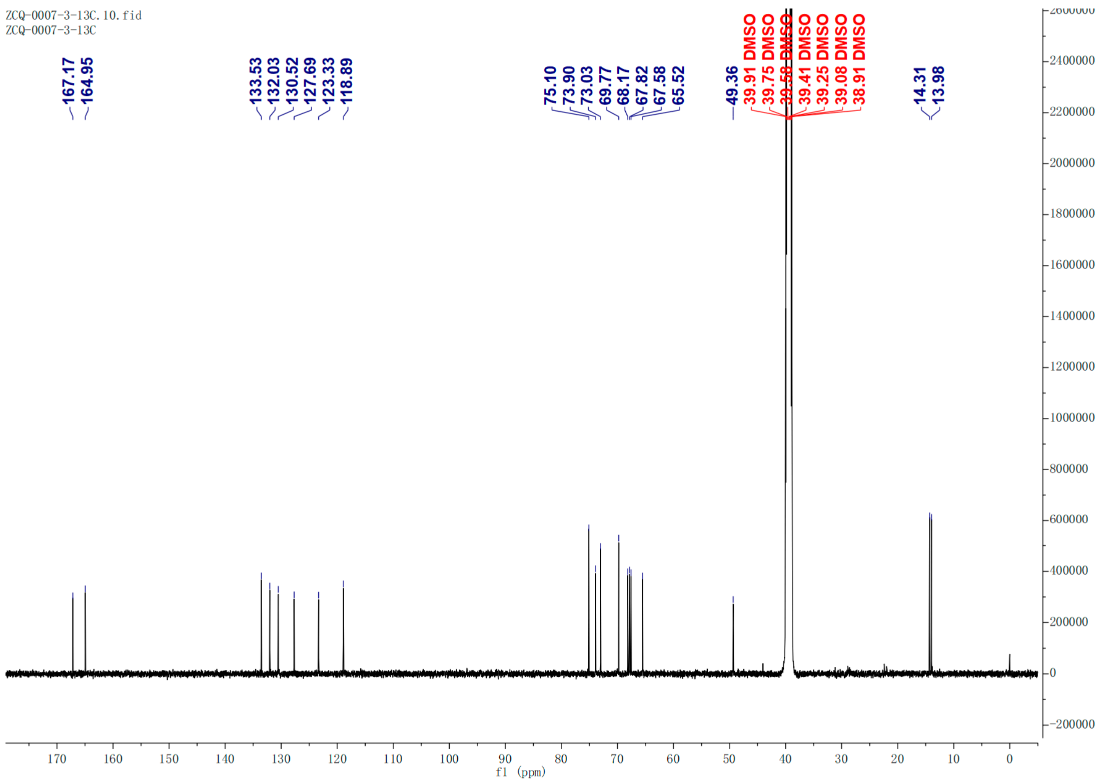

Figure S41. ^{13}C NMR (125 MHz, DMSO-d_6) spectrum of corallomycetellain E (**5**).

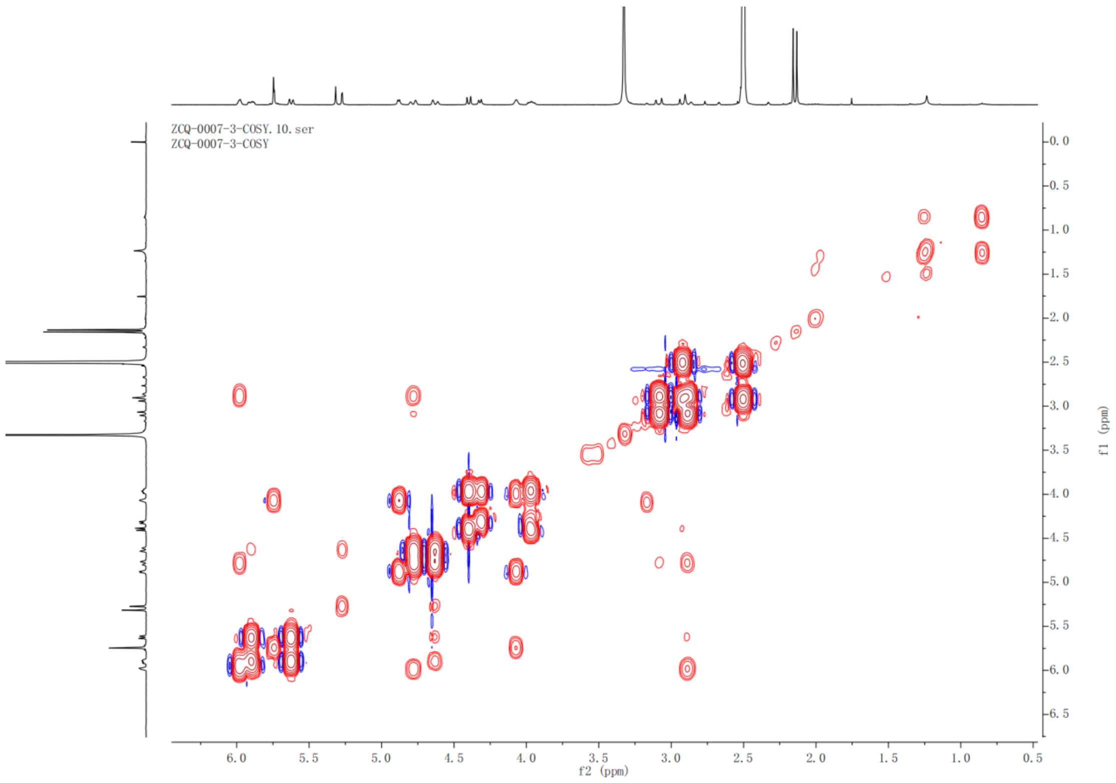

Figure S42. ^1H-^1H COSY spectrum of corallomycetellain E (**5**).

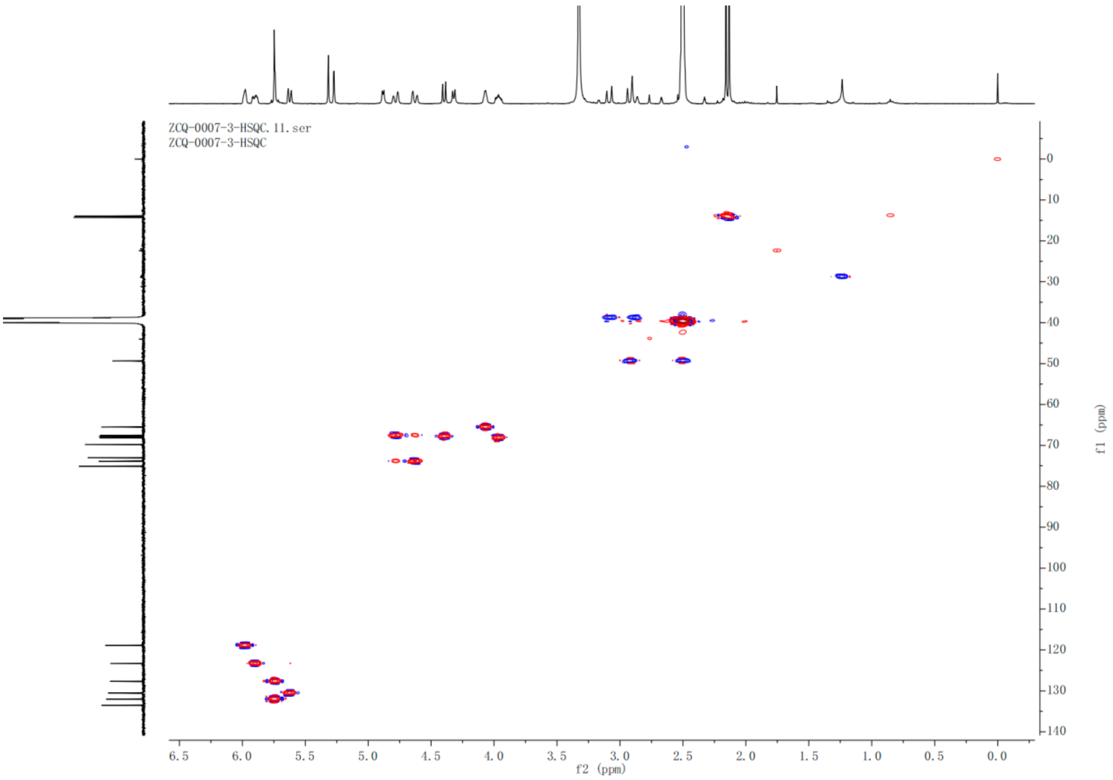

Figure S43. HSQC spectrum of corallomycetellain E (**5**).

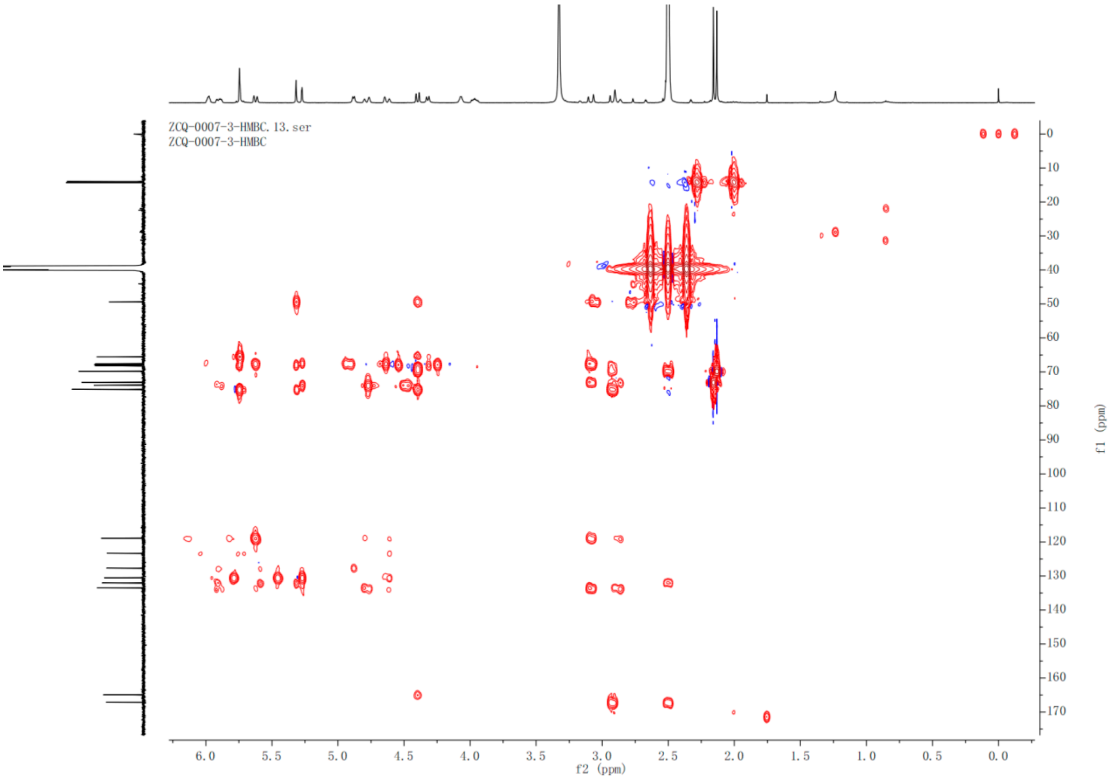

Figure S44. HMBC spectrum of corallomycetellain E (**5**).

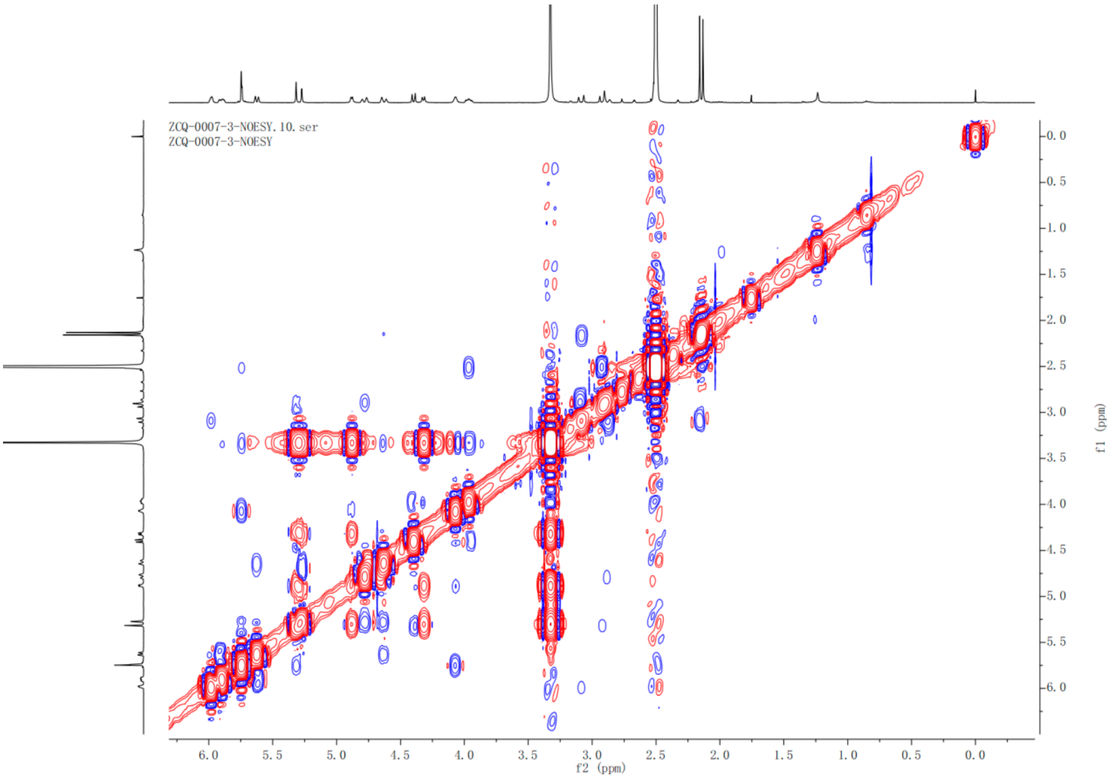

Figure S45. NOESY spectrum of corallomycetellain E (**5**).

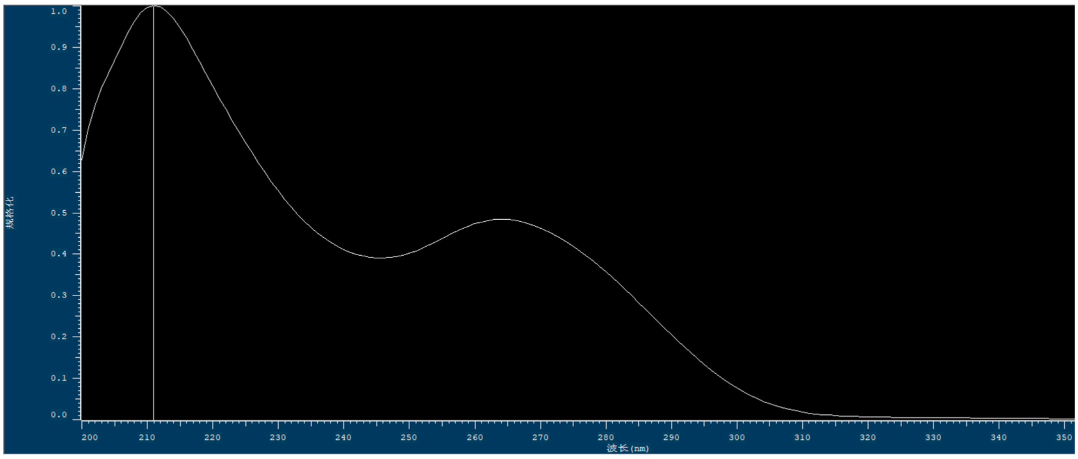

Figure S46. The UV spectrum of corallomycetellain E (**5**).

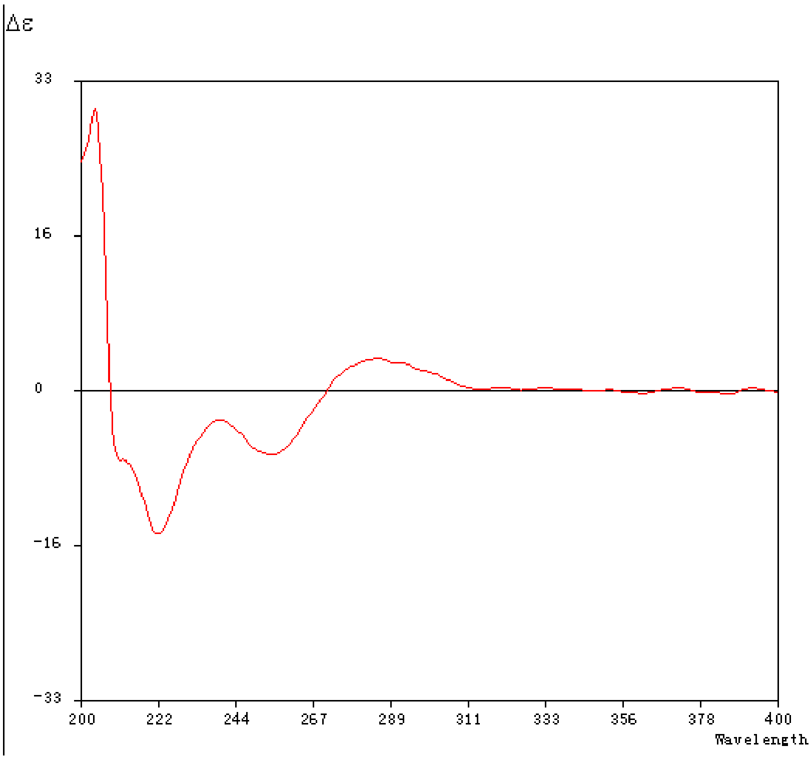

Figure S47. The CD spectrum of corallomycetellain E (**5**).

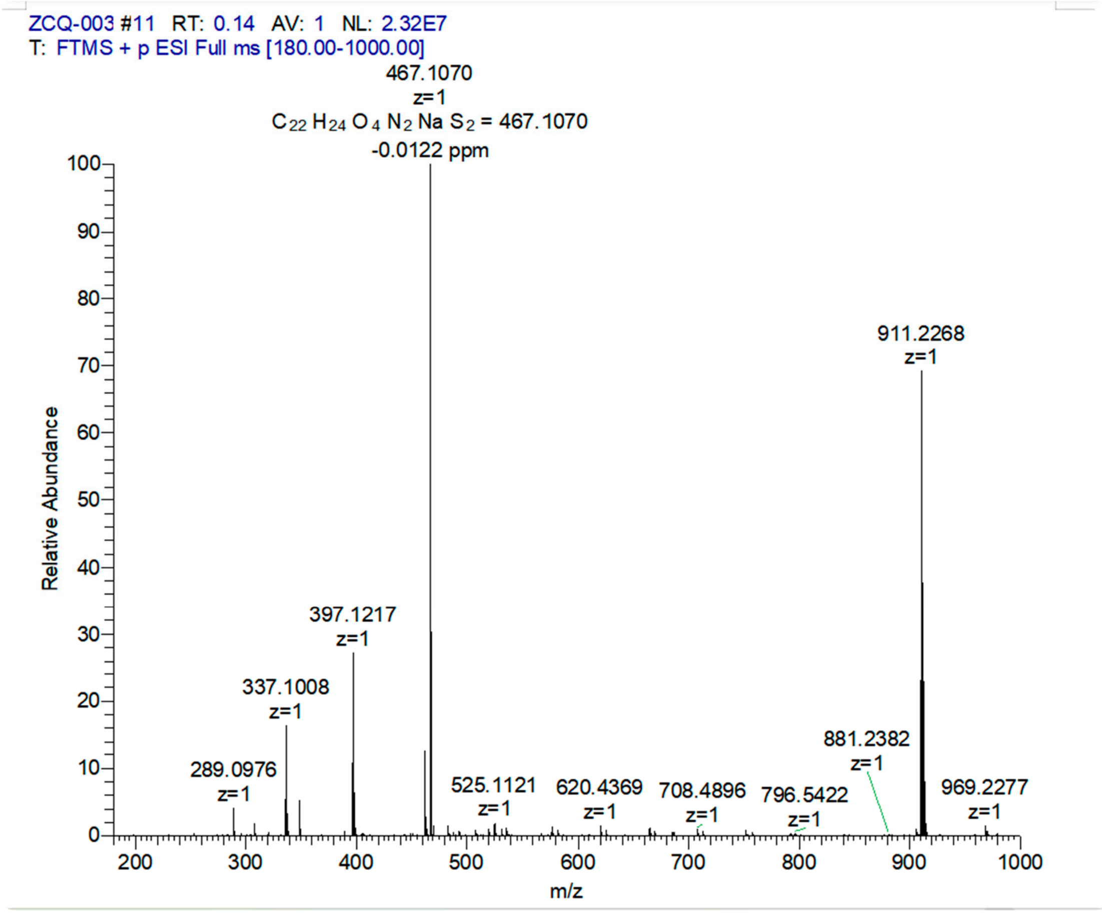

Figure S48. The HRESIMS spectrum of corallomycetellain F (**6**).

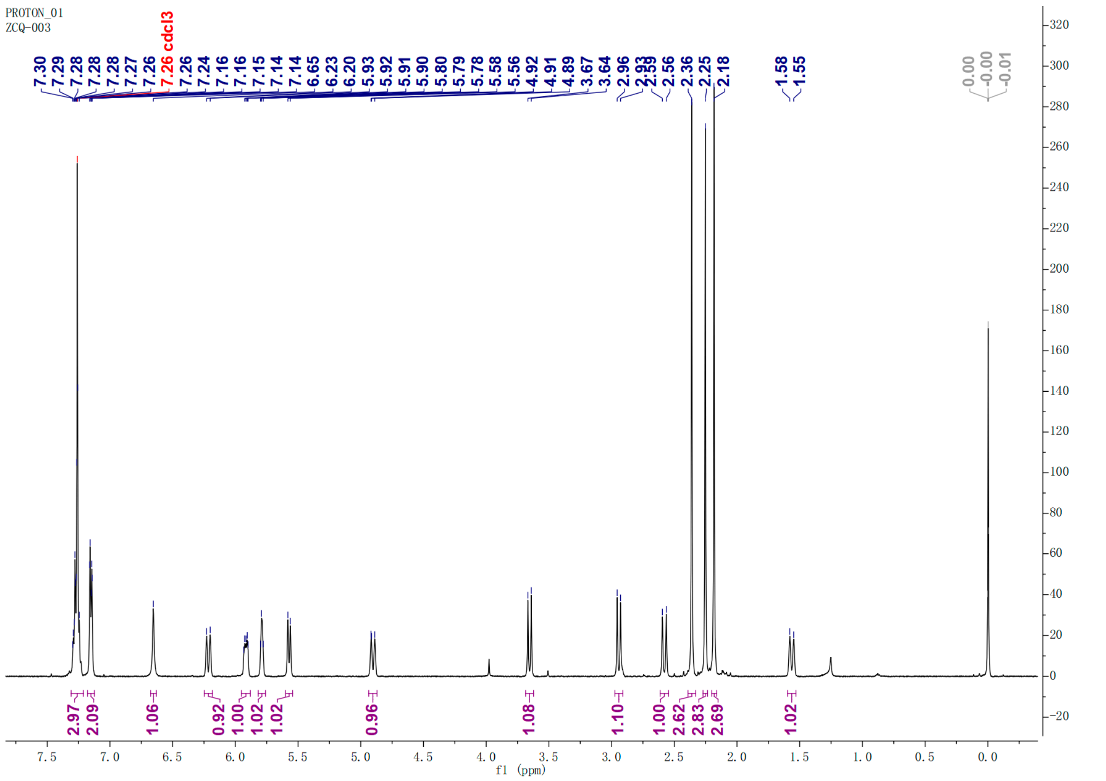

Figure S49. ^{1}H NMR (500 MHz, $CDCl_3$) spectrum of corallomycetellain F (**6**).

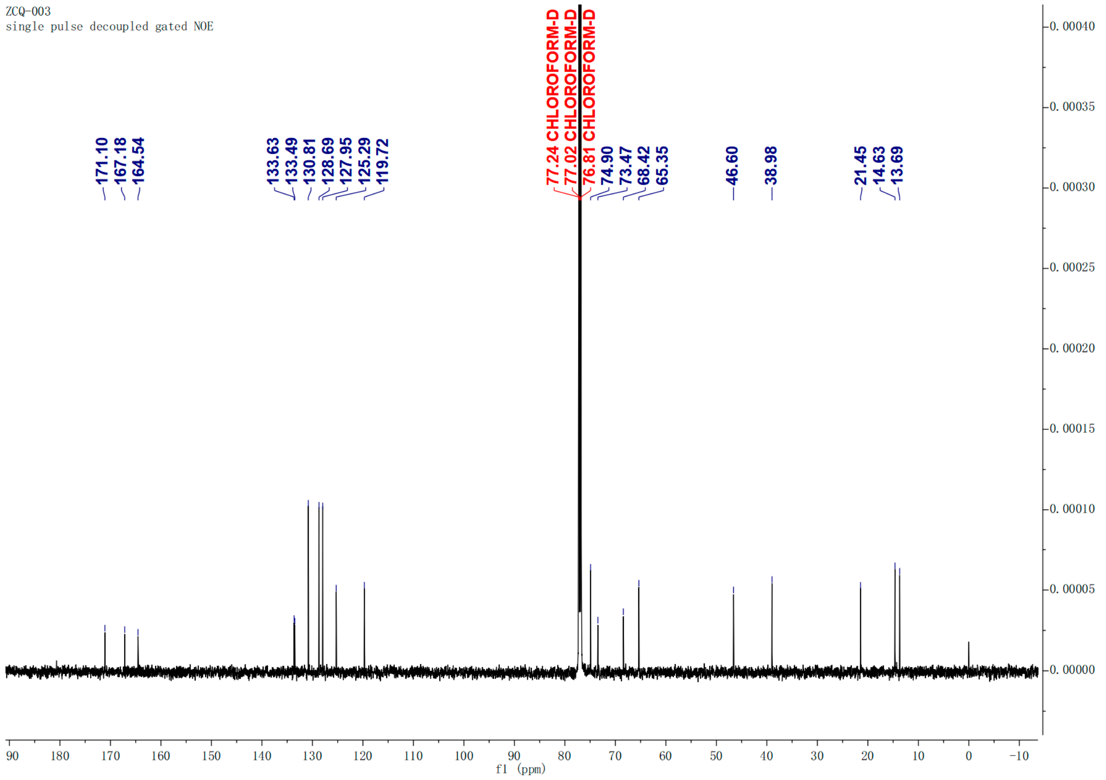

Figure S50. ^{13}C NMR (125 MHz, $CDCl_3$) spectrum of corallomycetellain F (**6**).

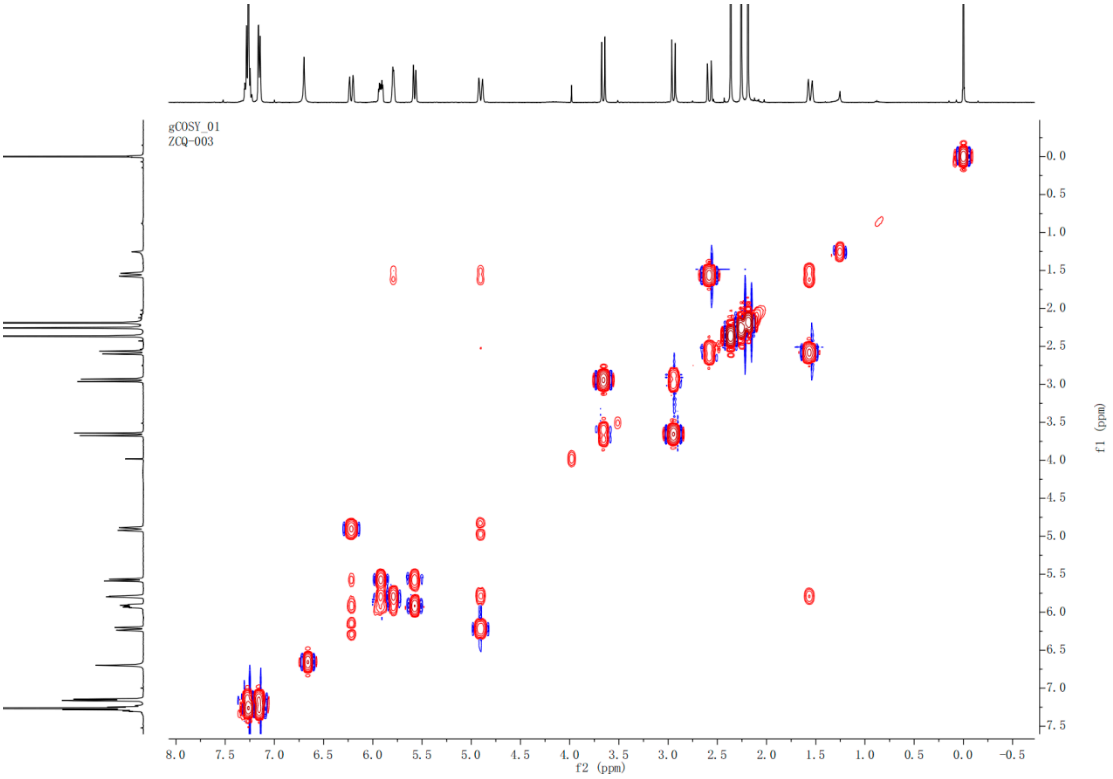

Figure S51. ^{1}H-^{1}H COSY spectrum of corallomycetellain F (**6**).

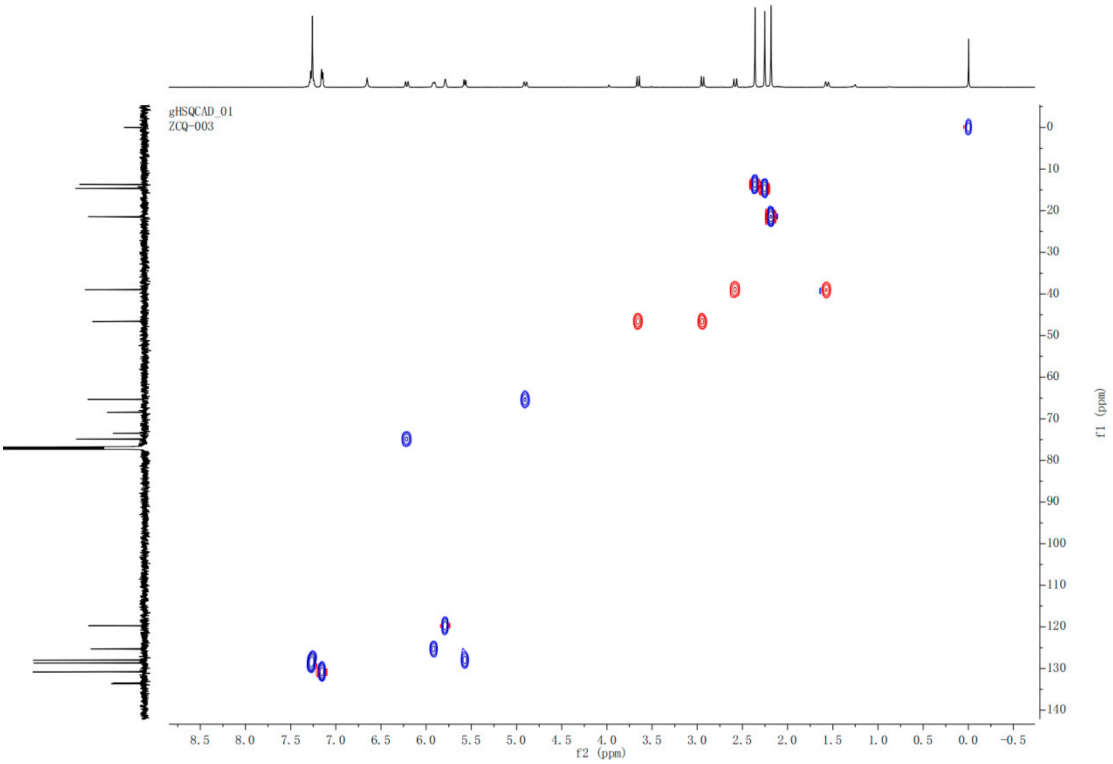

Figure S52. HSQC spectrum of corallomycetellain F (**6**).

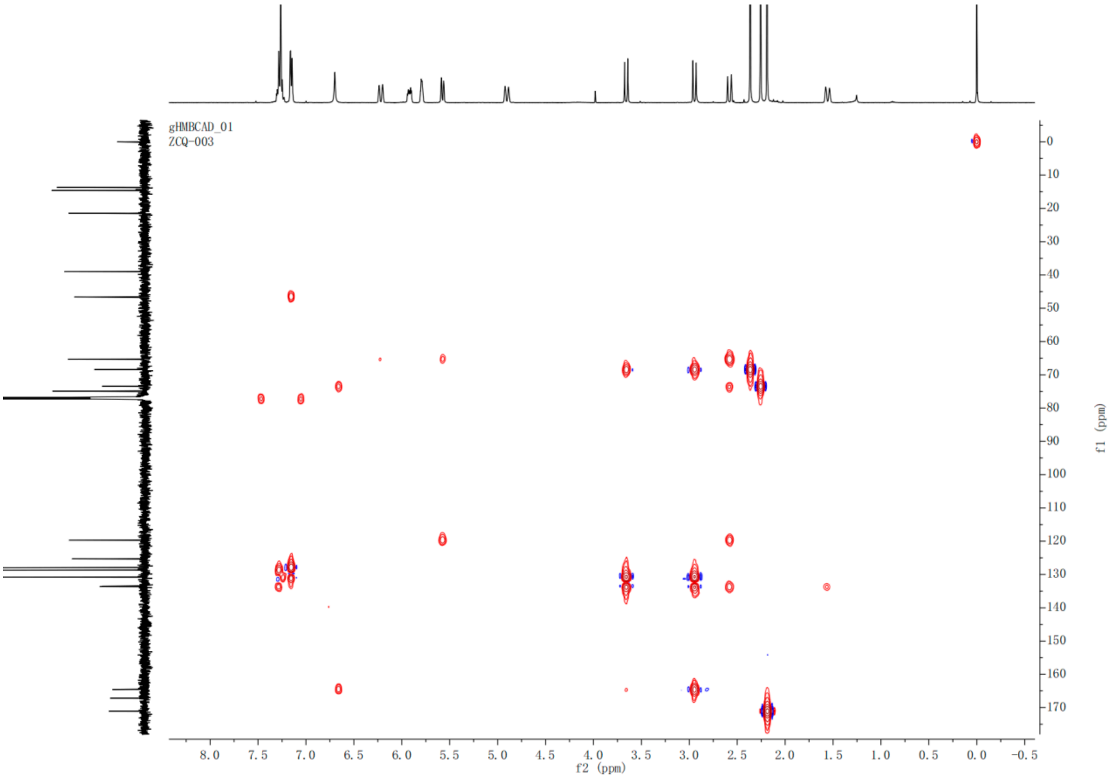

Figure S53. HMBC spectrum of corallomycetellain F (**6**).

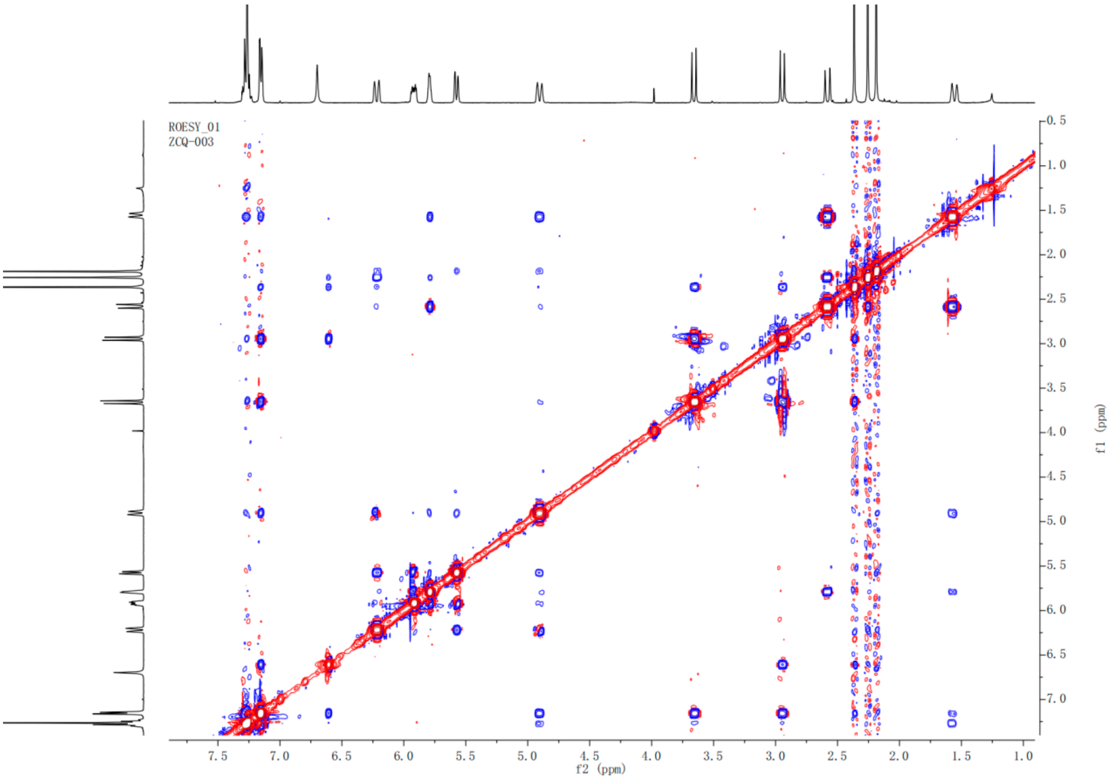

Figure S54. ROESY spectrum of corallomycetellain F (**6**).

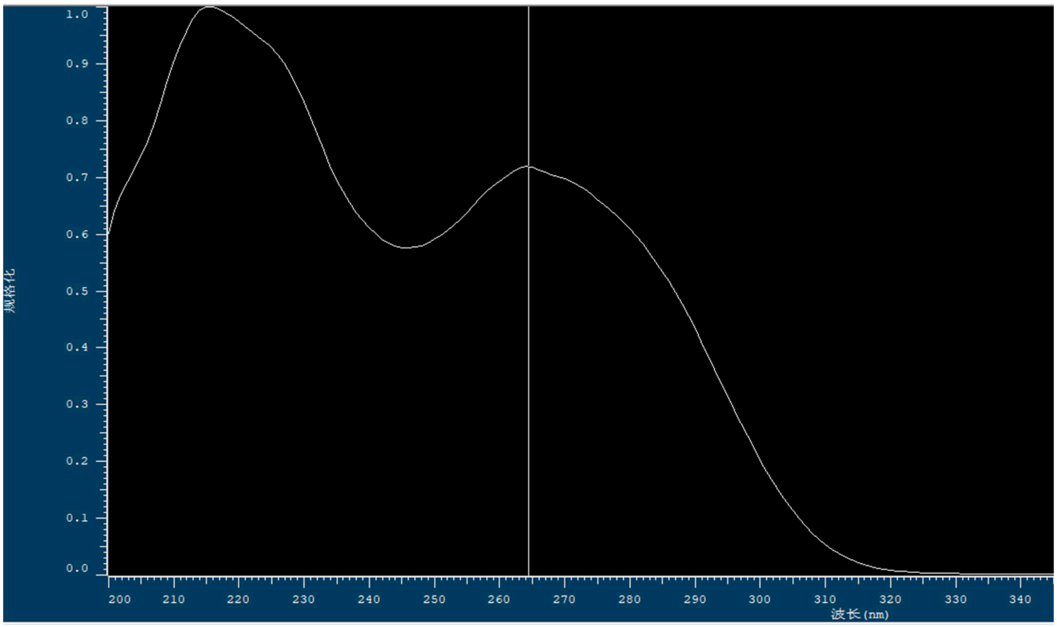

Figure S55. The UV spectrum of corallomycetellain F (**6**).

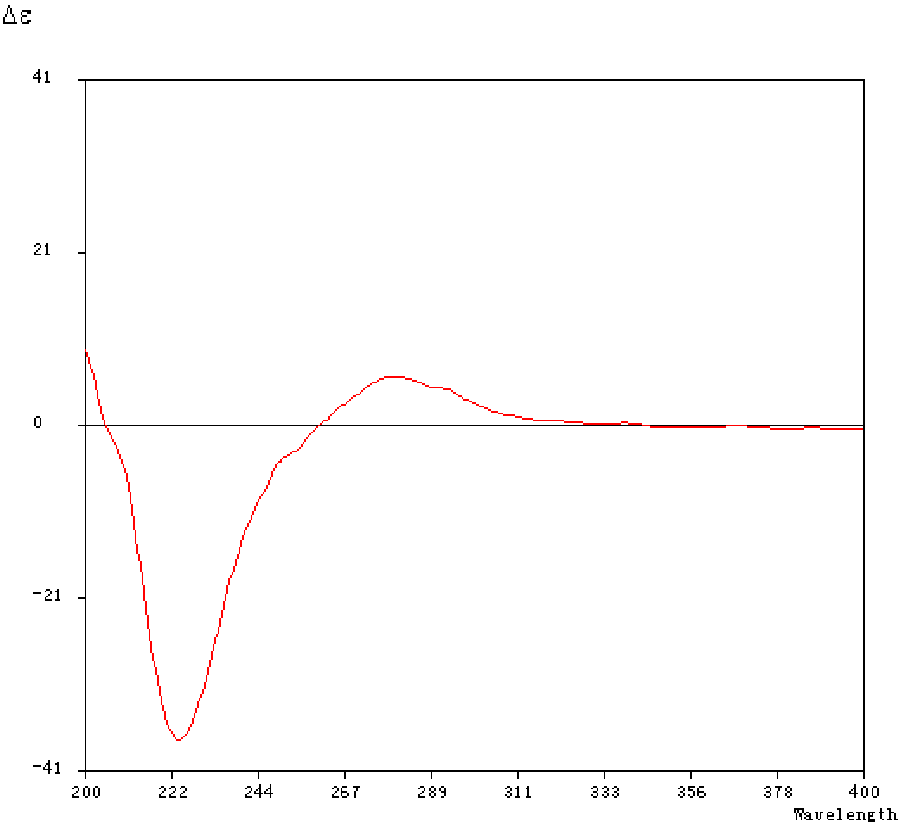

Figure S56. The CD spectrum of corallomycetellain F (**6**).

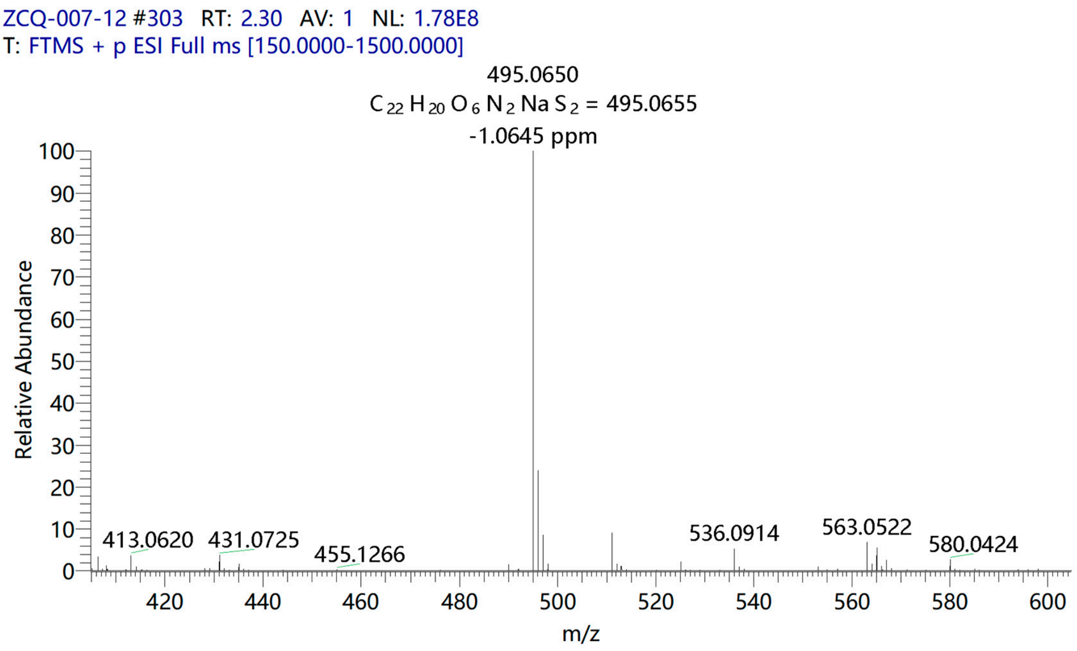

Figure S57. The HRESIMS spectrum of corallomycetellain G (**7**).

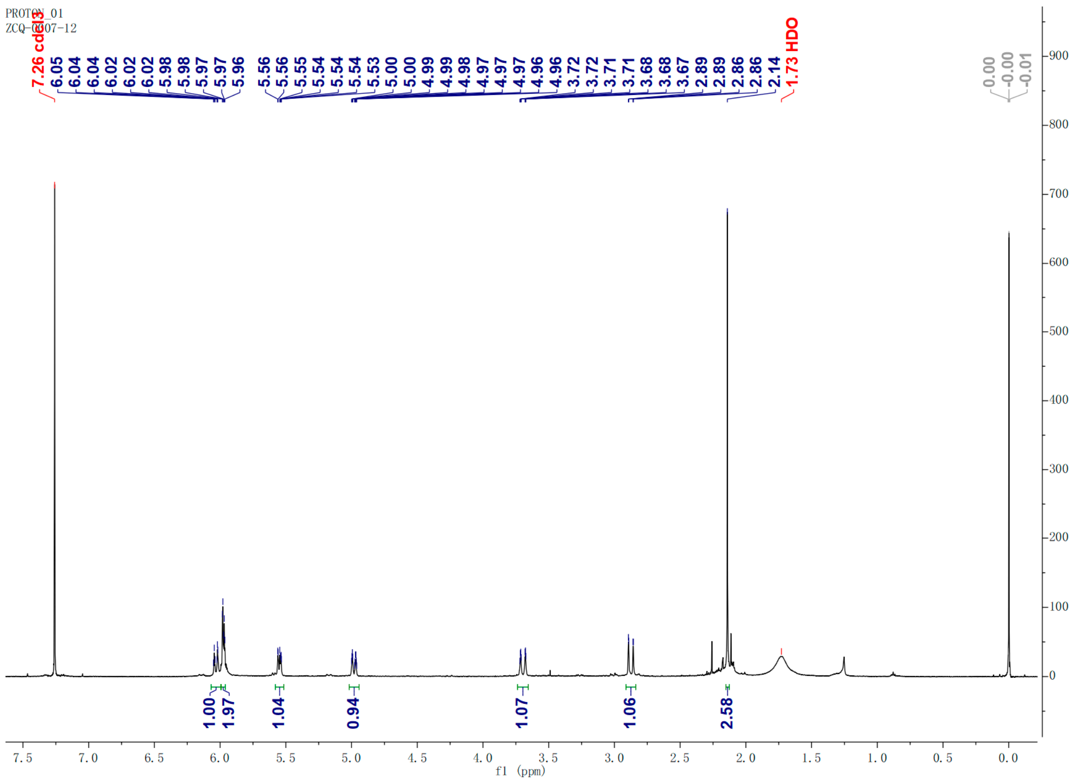

Figure S58. ^{1}H NMR (600 MHz, $CDCl_3$) spectrum of corallomycetellain G (**7**).

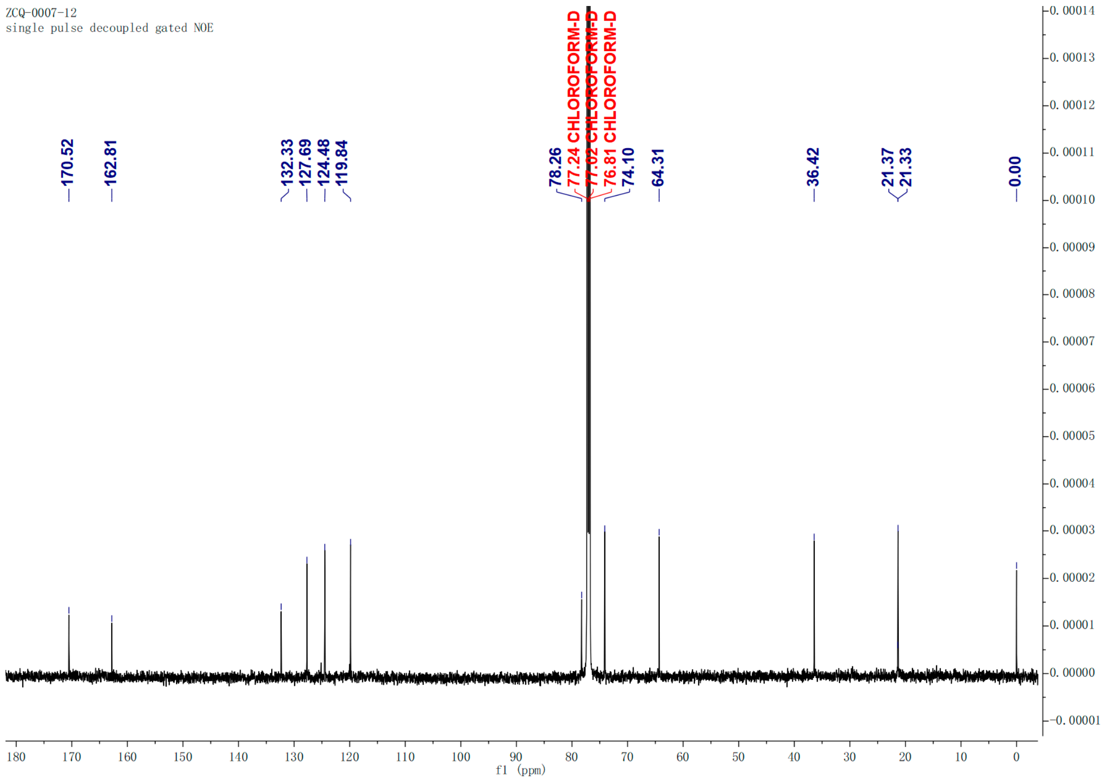

Figure S59. ^{13}C NMR (150 MHz, $CDCl_3$) spectrum of corallomycetellain G (**7**).

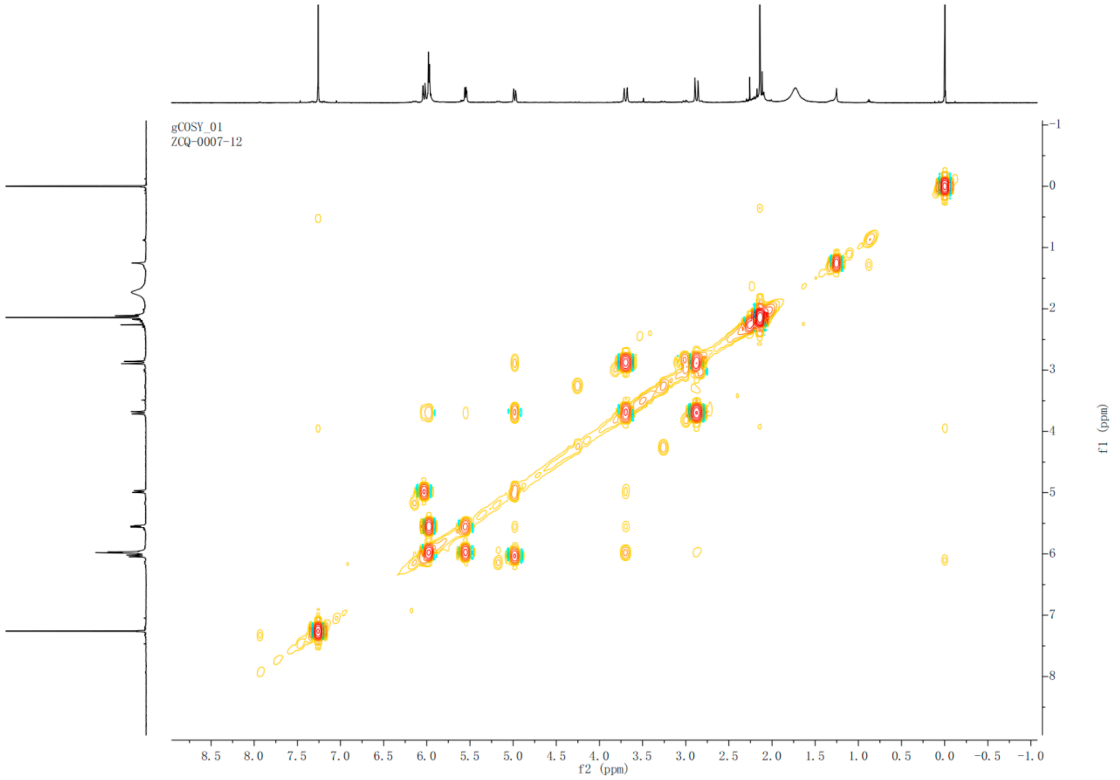

Figure S60. ^{1}H-^{1}H COSY spectrum of corallomycetellain G (**7**).

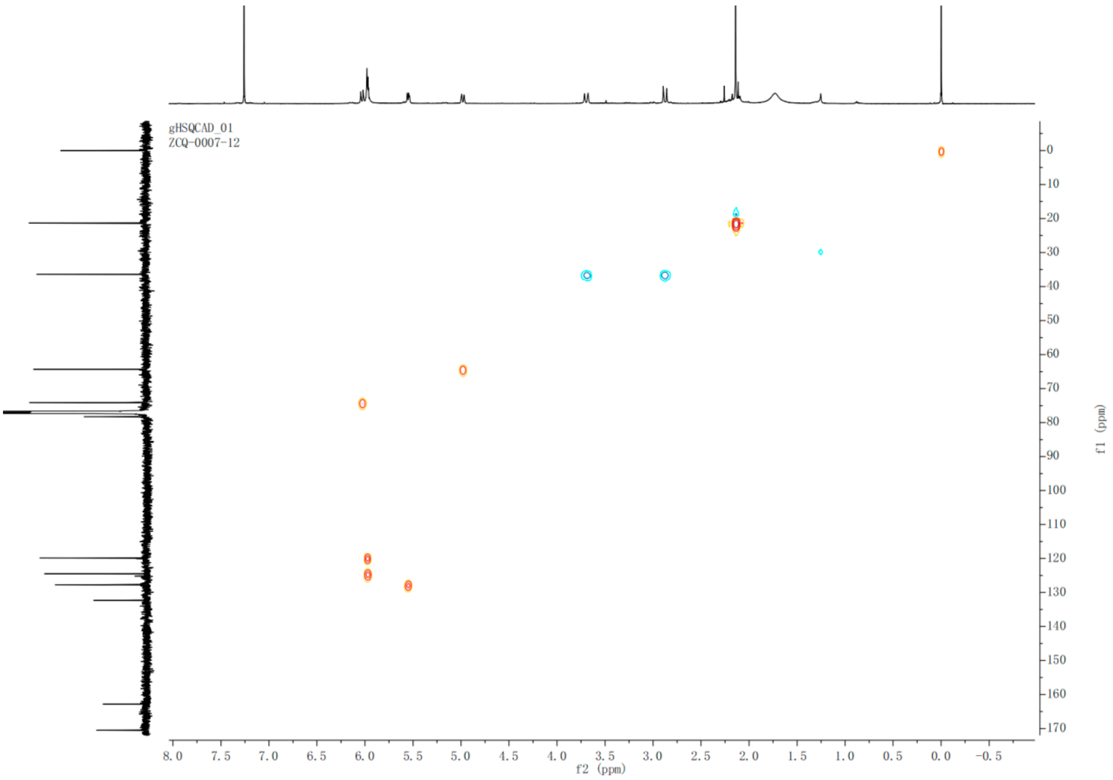

Figure S61. HSQC spectrum of corallomycetellain G (**7**).

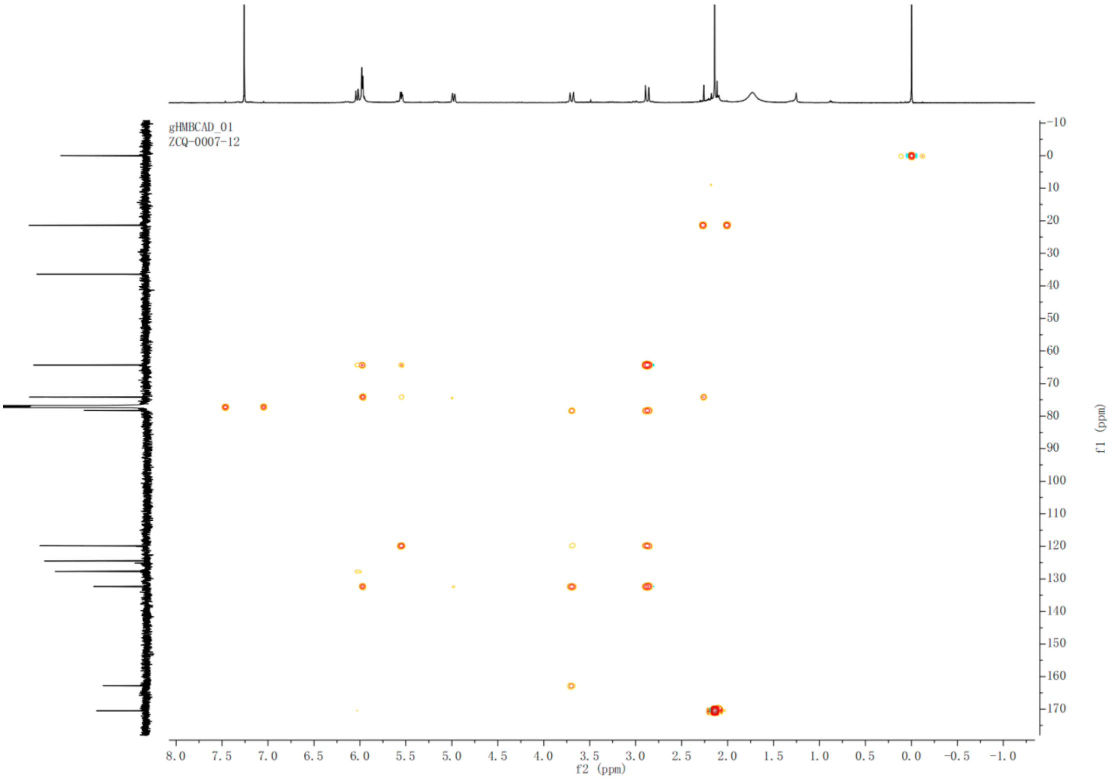

Figure S62. HMBC spectrum of corallomycetellain G (7).

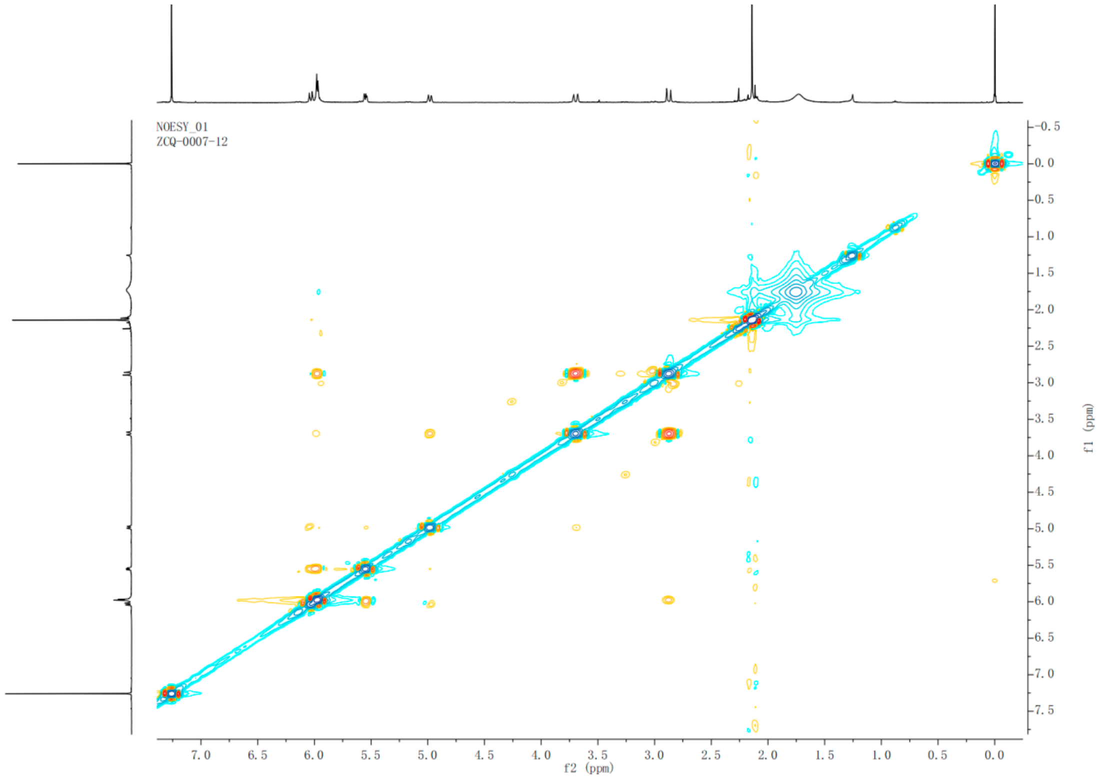

Figure S63. ROESY spectrum of corallomycetellain G (**7**).

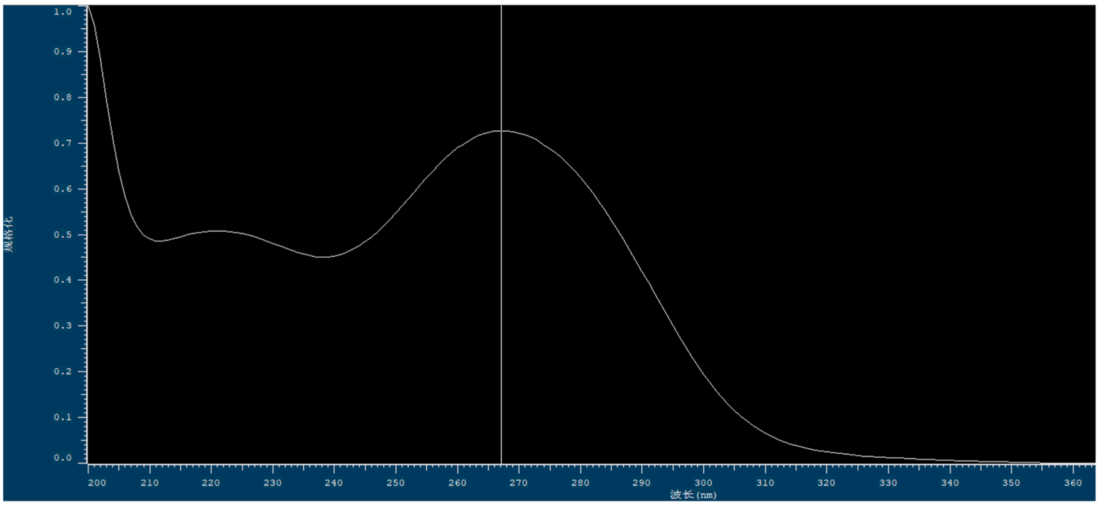

Figure S64. The UV spectrum of corallomycetellain G (**7**).

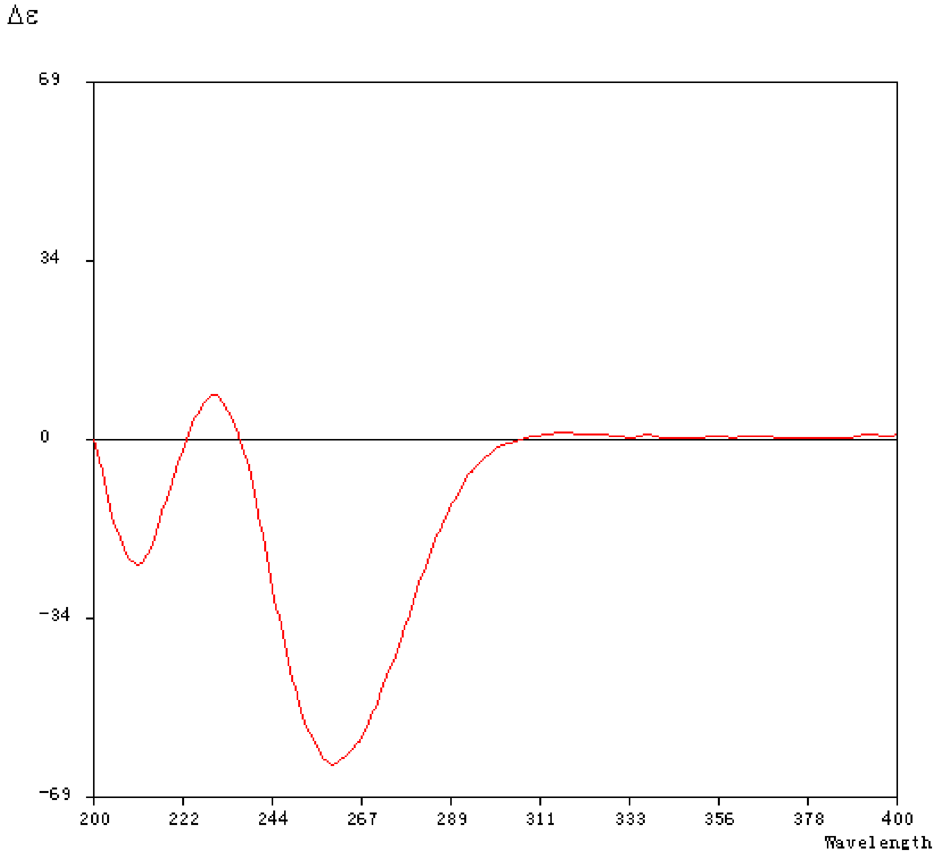

Figure S65. The CD spectrum of corallomycetellain G (**7**).

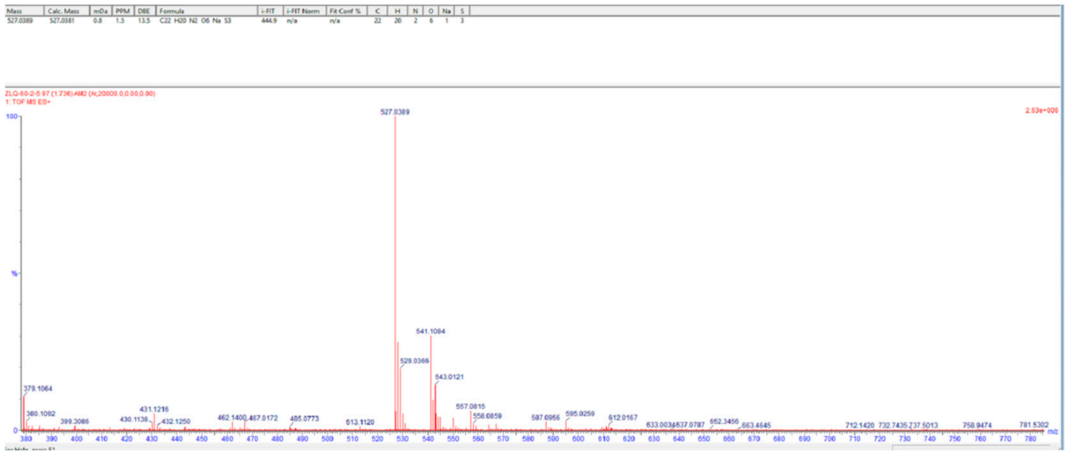

Figure S66. The HRESIMS spectrum of corallomycetellain H (**8**).

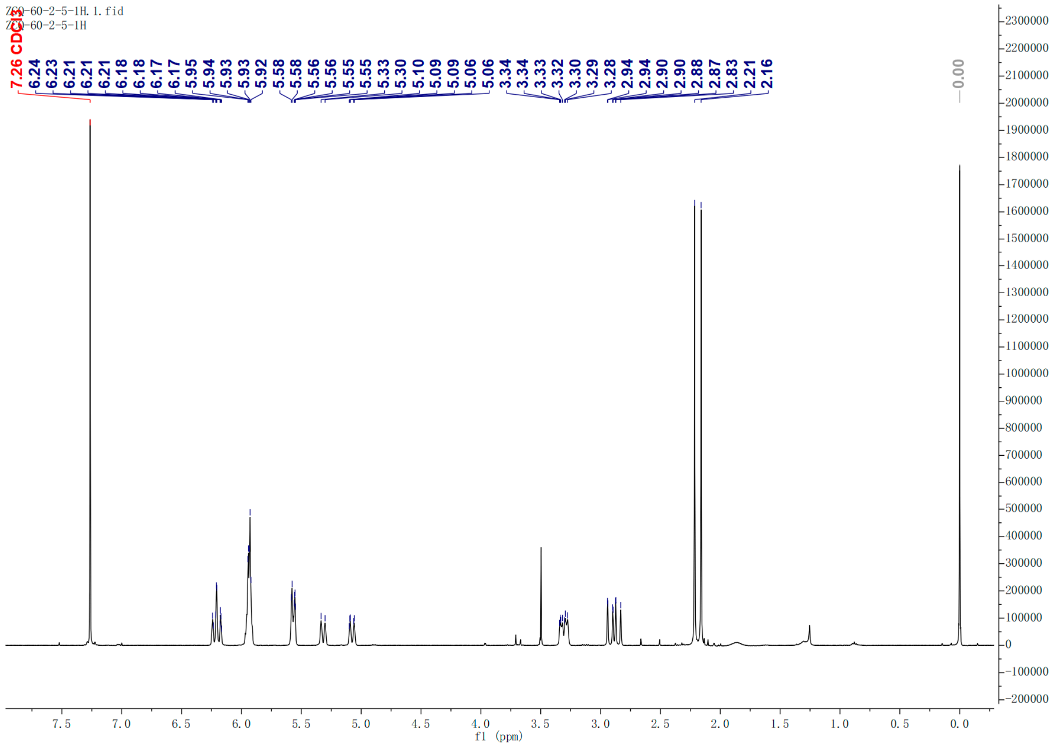

Figure S67. ^{1}H NMR (600 MHz, $CDCl_3$) spectrum of corallomycetellain H (**8**).

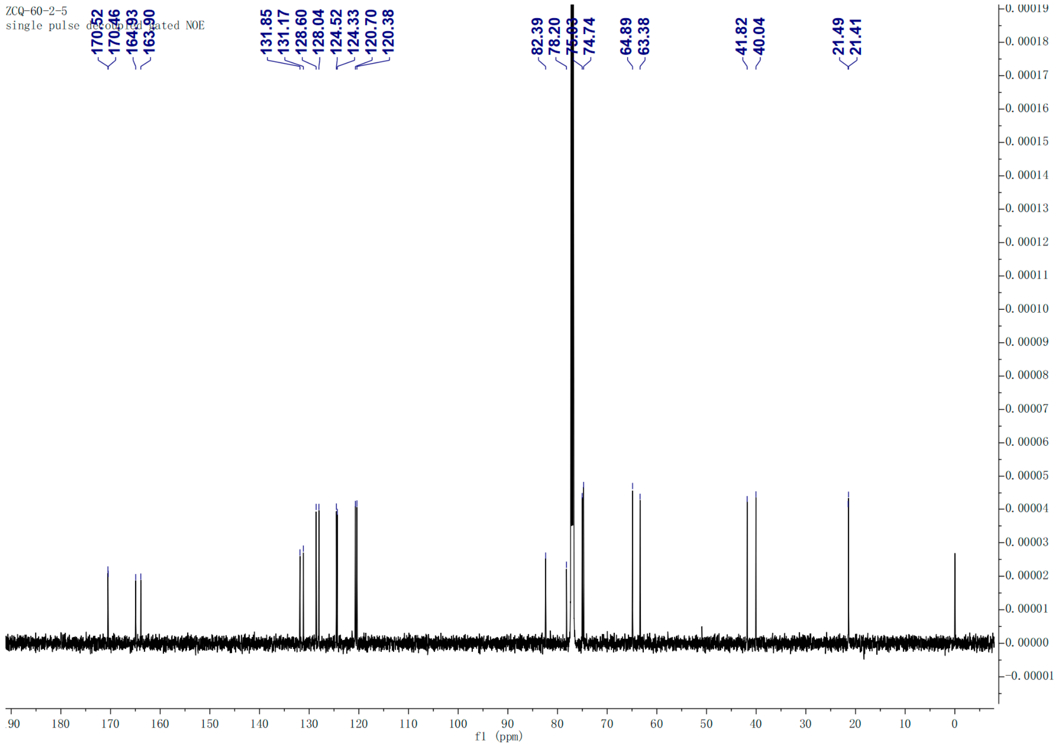

Figure S68. ^{13}C NMR (150 MHz, $CDCl_3$) spectrum of corallomycetellain H (**8**).

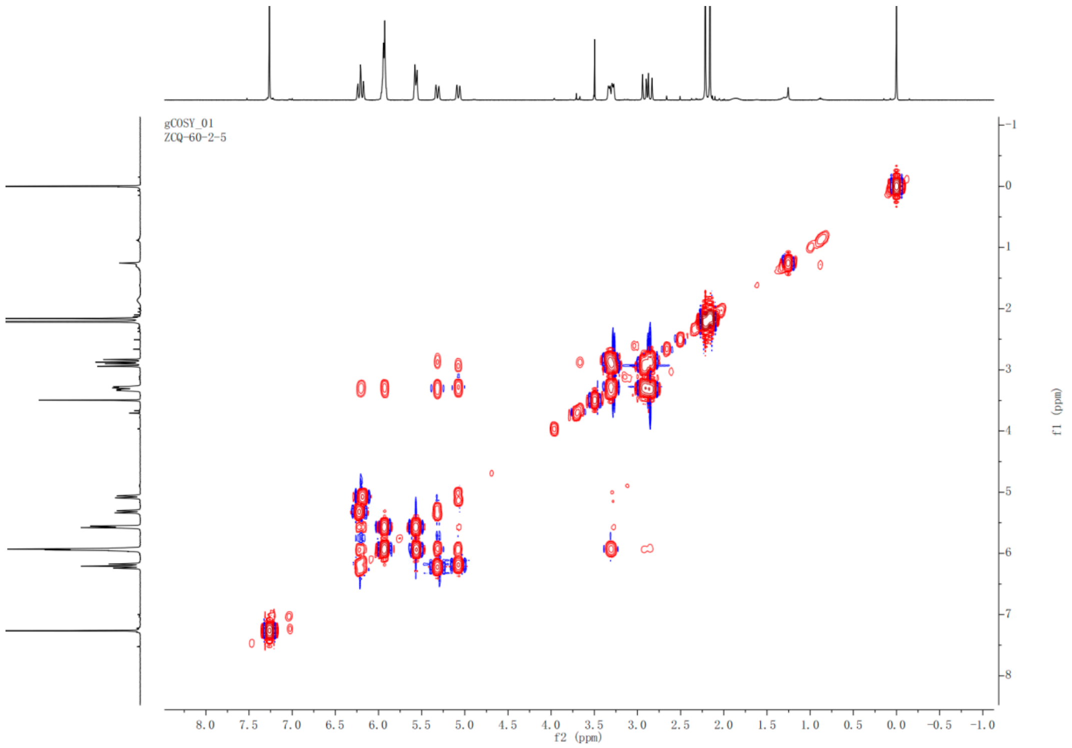

Figure S69. ^{1}H-^{1}H COSY spectrum of corallomycetellain H (**8**).

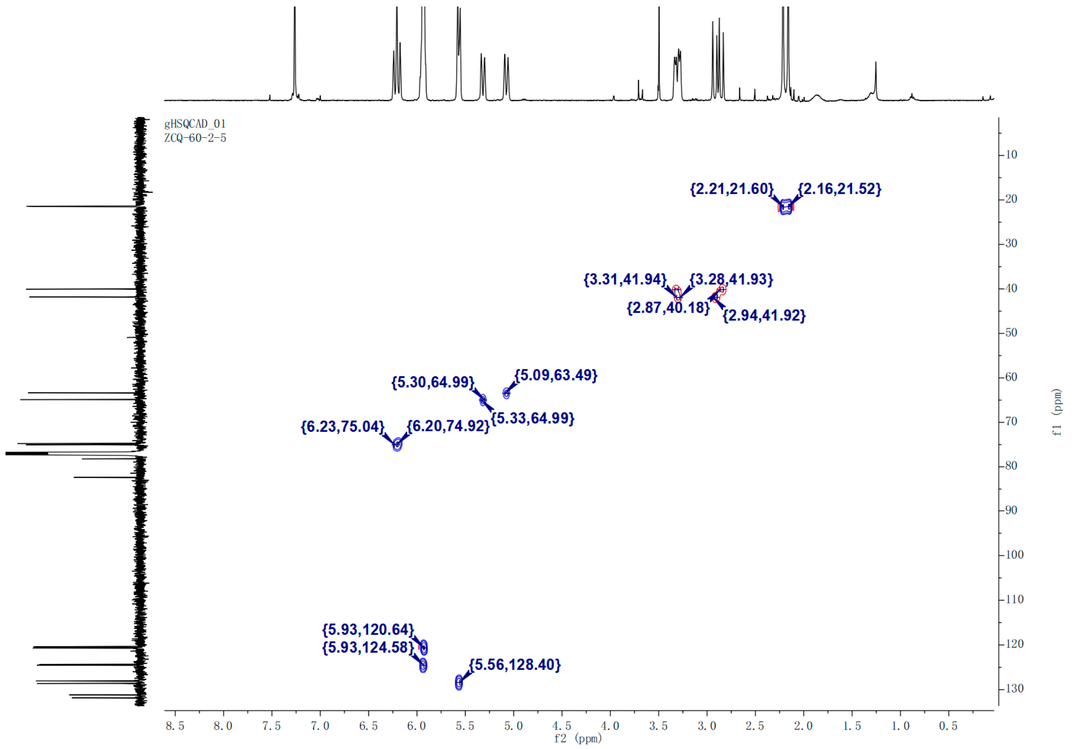

Figure S70. HSQC spectrum of corallomycetellain H (**8**).

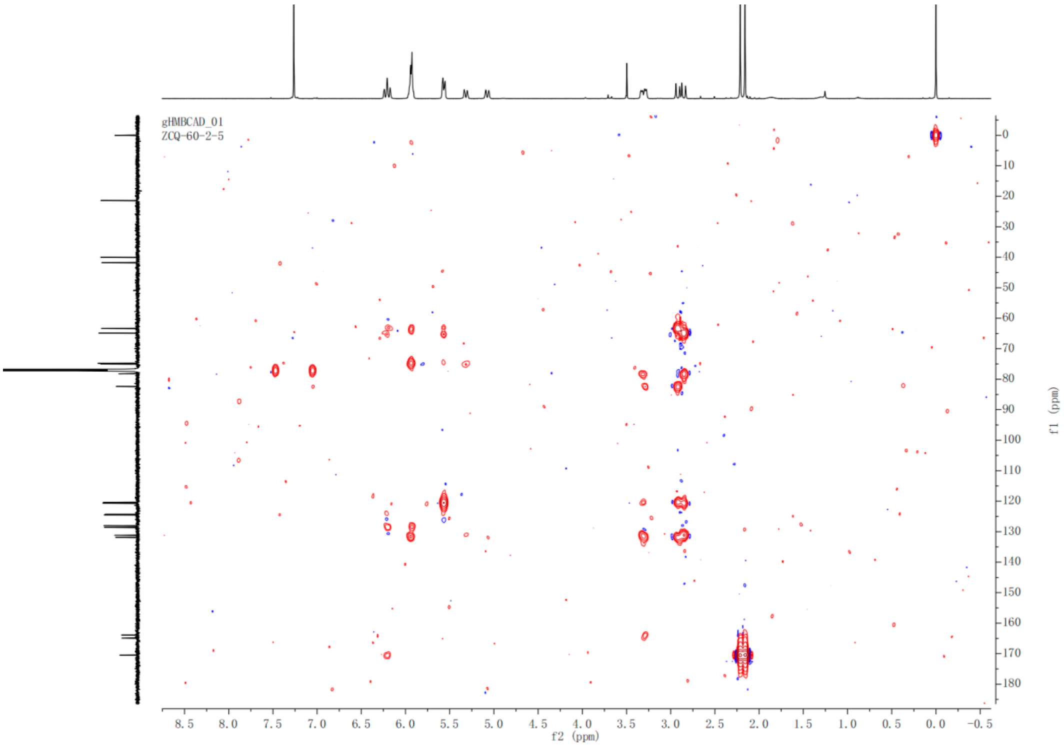

Figure S71. HMBC spectrum of corallomycetellain H (**8**).

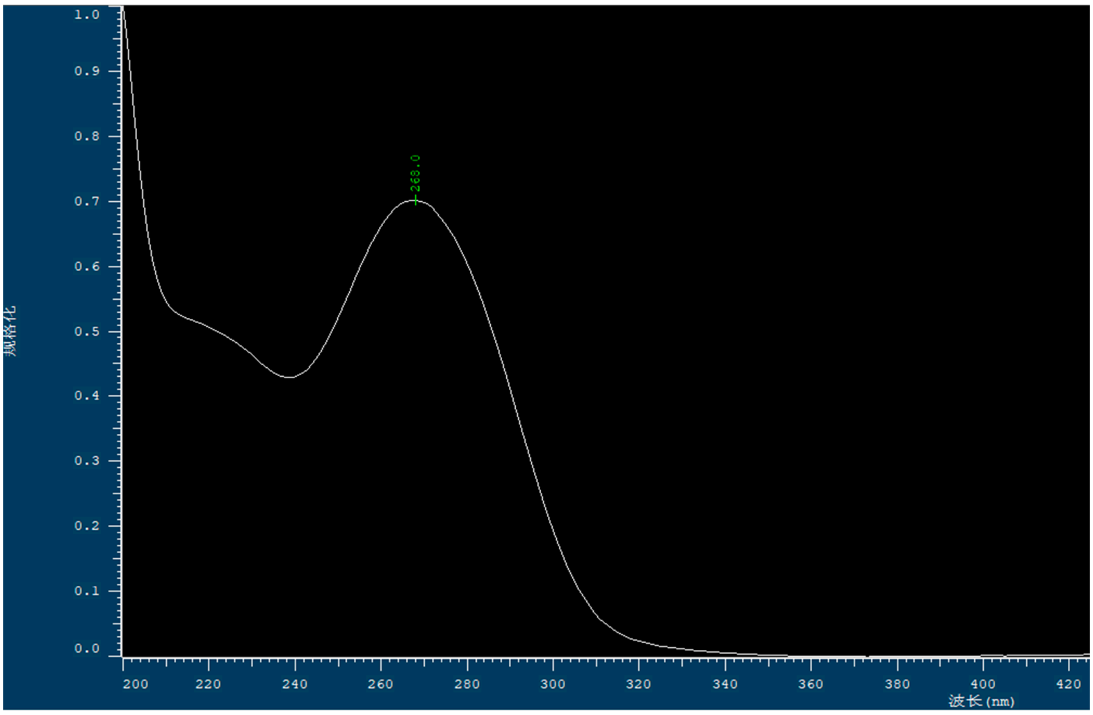

Figure S72. The UV spectrum of corallomycetellain H (**8**).

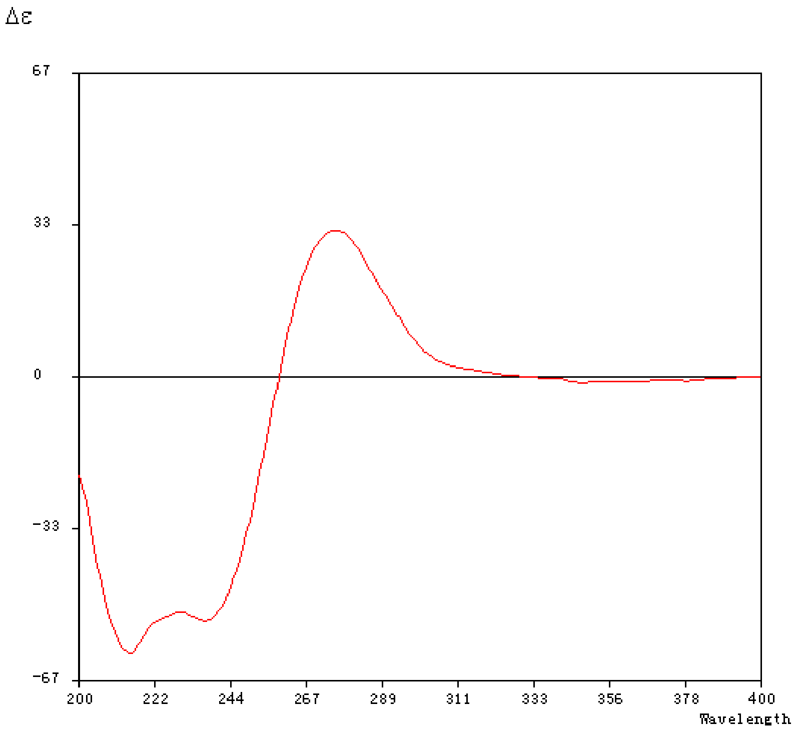

Figure S73. The CD spectrum of corallomycetellain H (**8**).

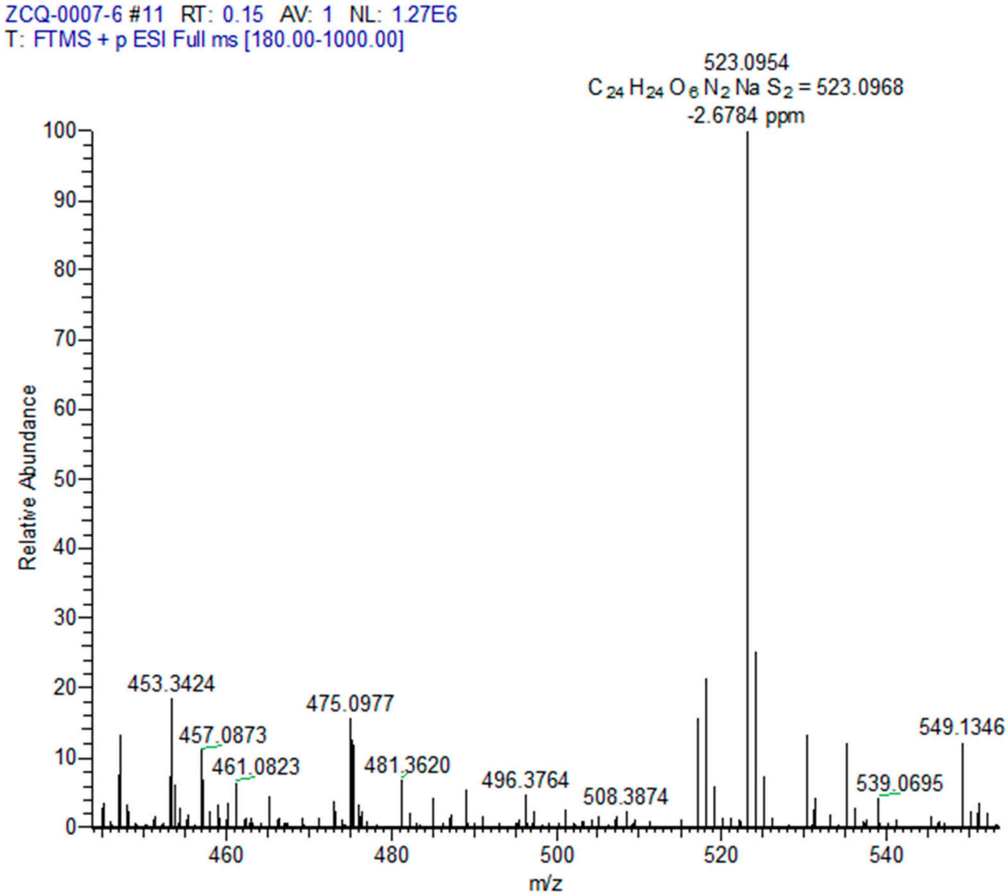

Figure S74. The HRESIMS spectrum of corallomycetellain I (**9**).

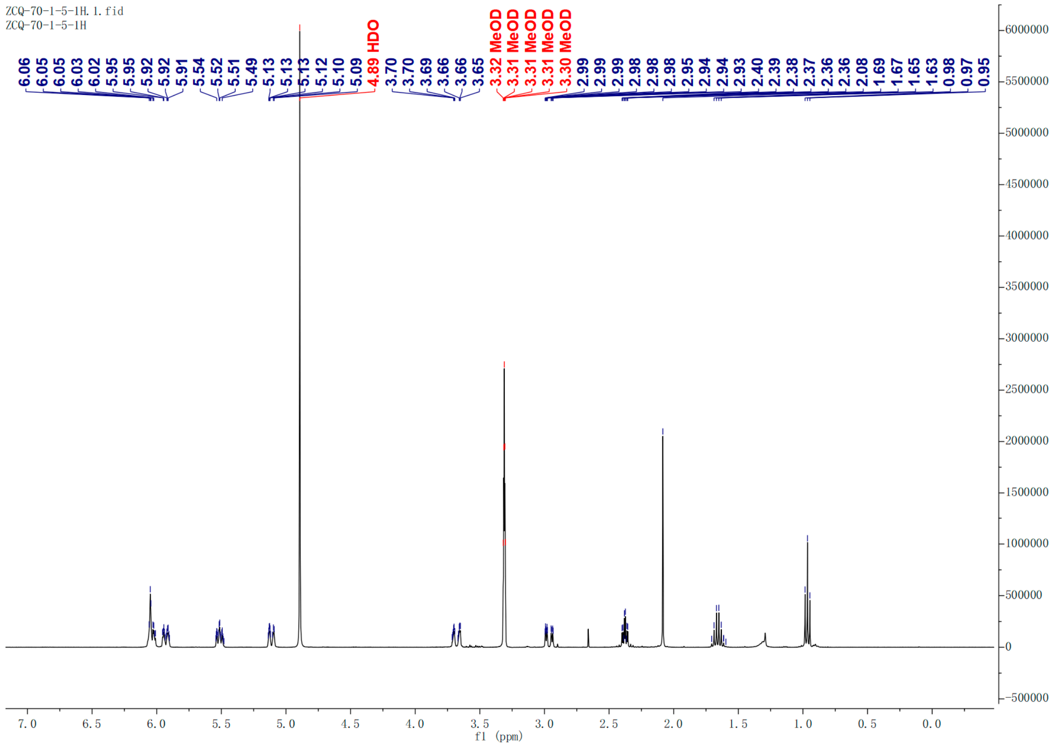

Figure S75. ^{1}H NMR (600 MHz, $CDCl_3$) spectrum of corallomycetellain I (**9**).

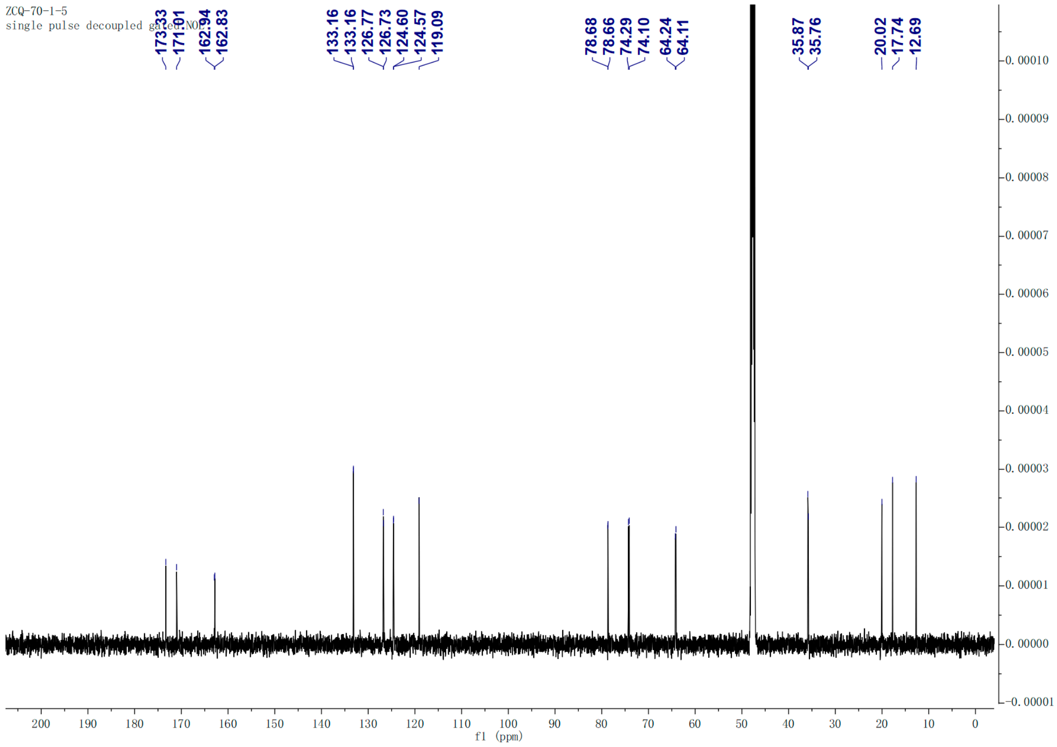

Figure S76. ^{13}C NMR (150 MHz, $CDCl_3$) spectrum of corallomycetellain I (**9**).

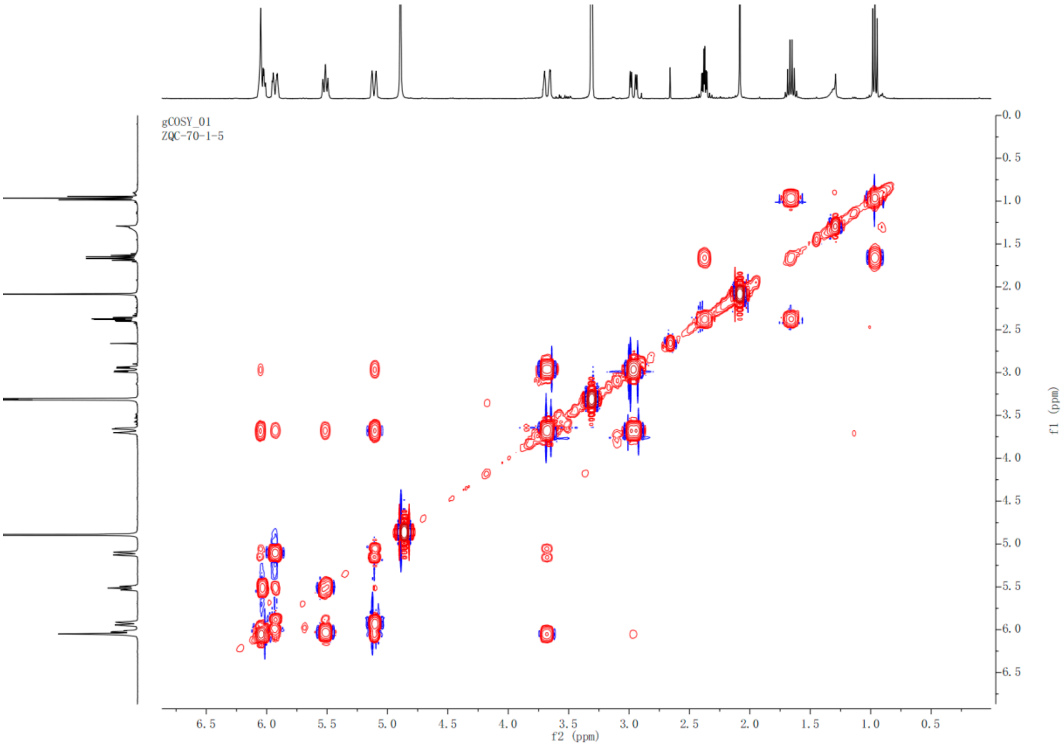

Figure S77. ^{1}H-^{1}H COSY spectrum of corallomycetellain I (**9**).

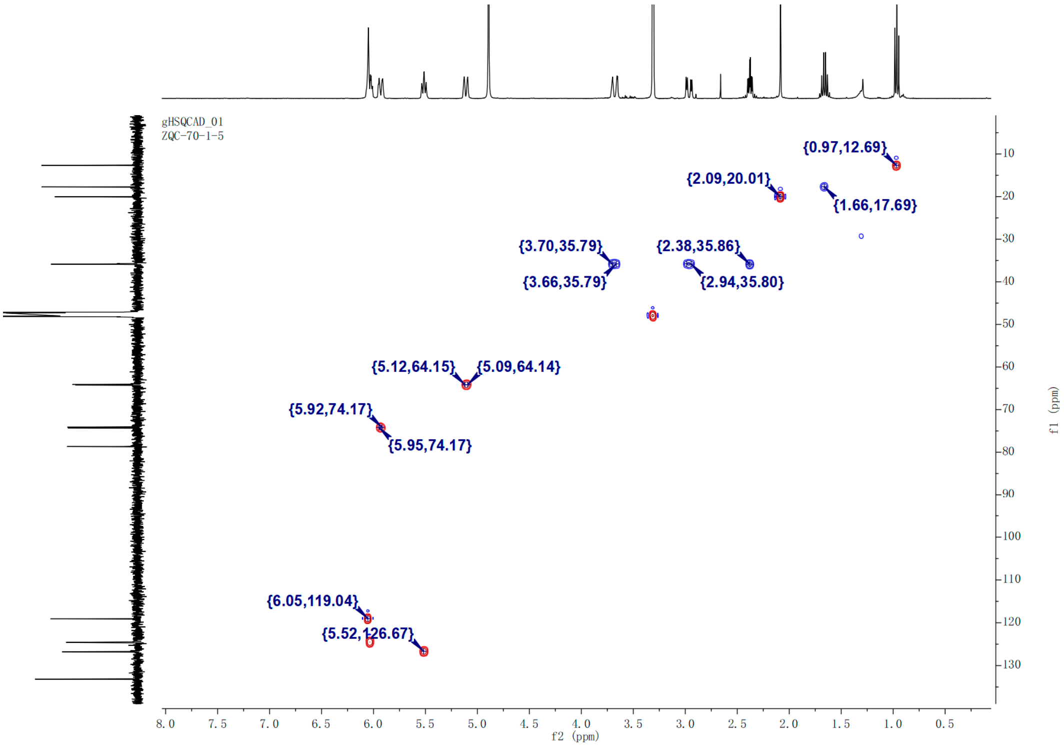

Figure S78. HSQC spectrum of corallomycetellain I (**9**).

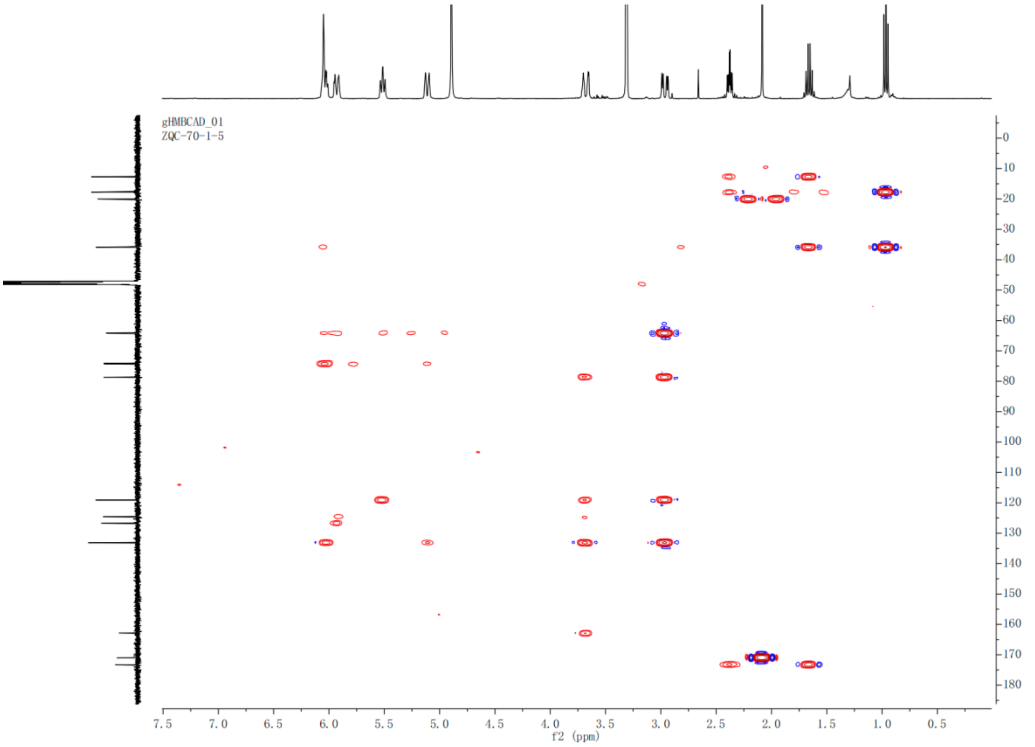

Figure S79. HMBC spectrum of corallomycetellain I (**9**).

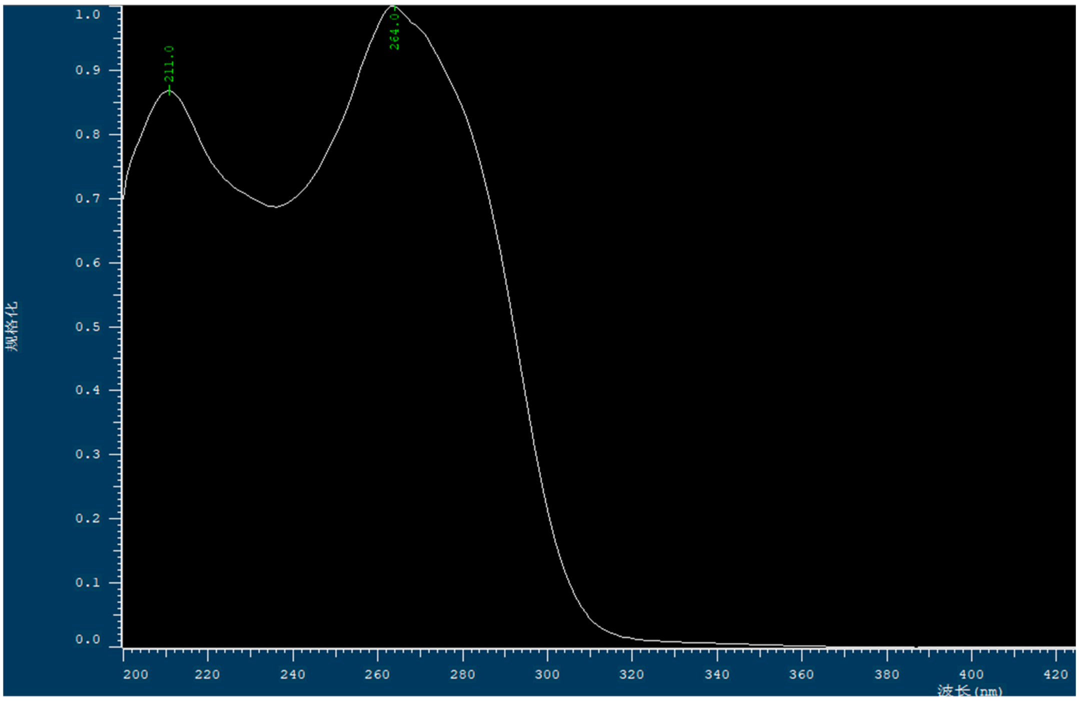

Figure S80. The UV spectrum of corallomycetellain I (**9**).

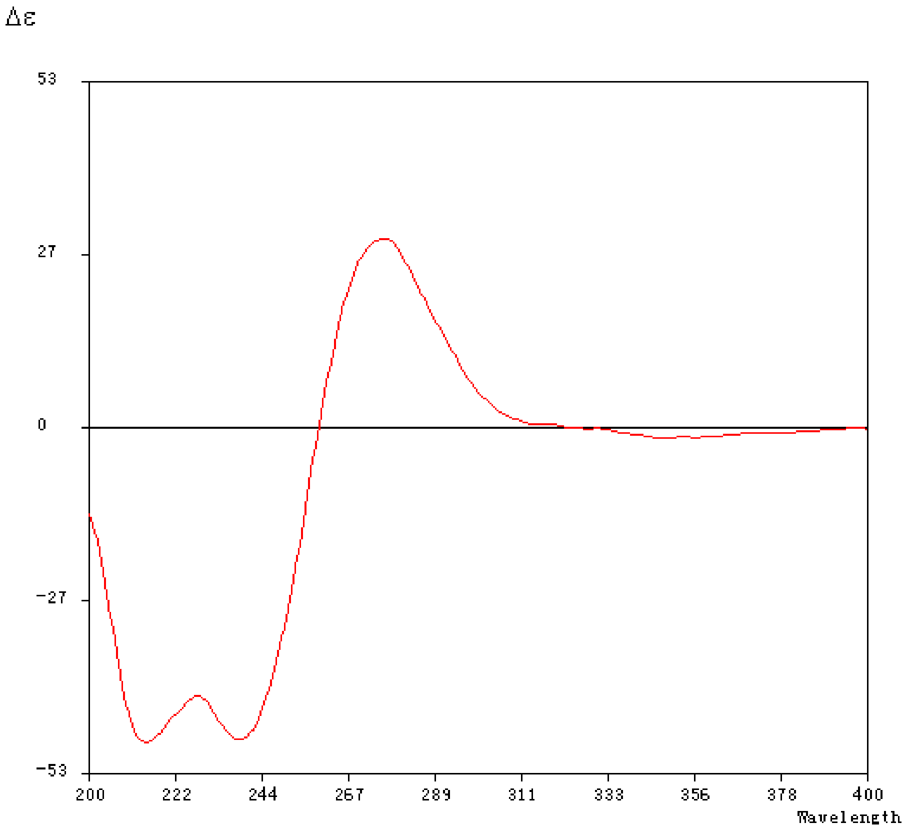

Figure S81. The CD spectrum of corallomycetellain I (**9**).

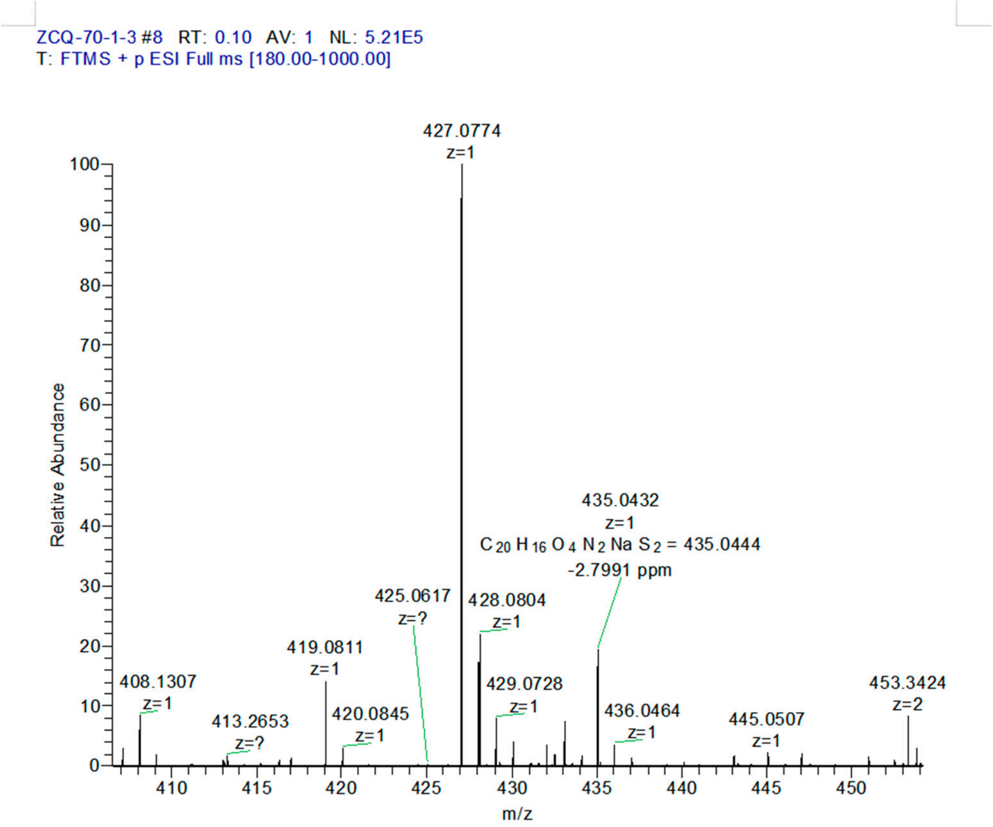

Figure S82. HRESIMS spectrum of corallomycetellain J (**10**).

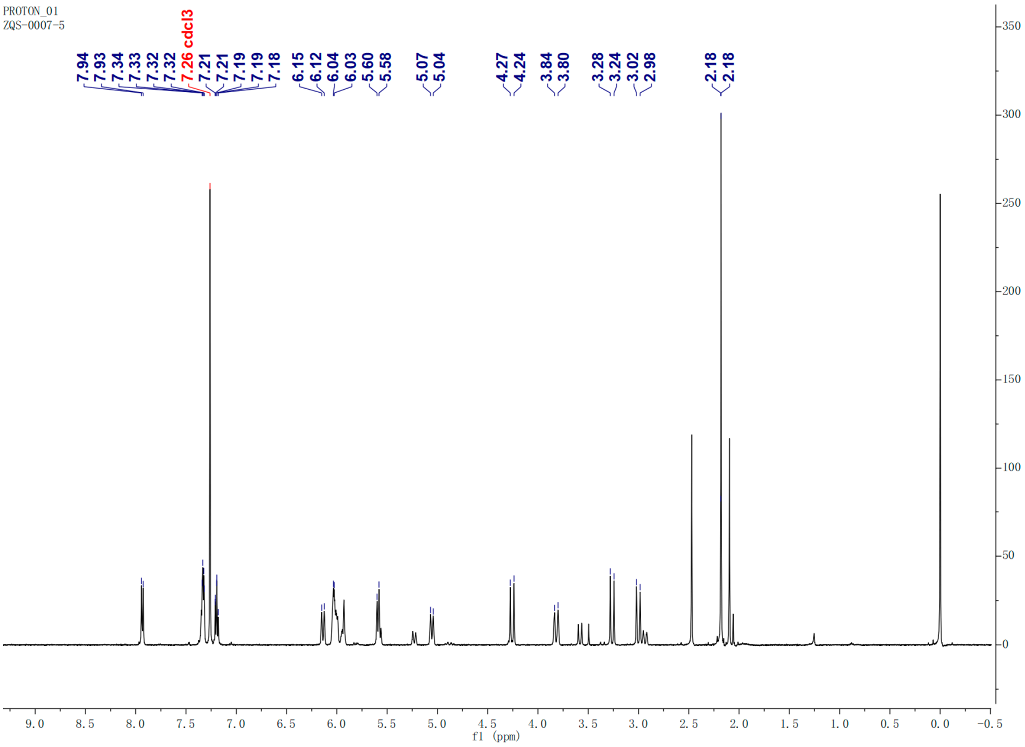

Figure S83. ^{1}H NMR (600 MHz, $CDCl_3$) spectrum of corallomycetellain J (**10**).

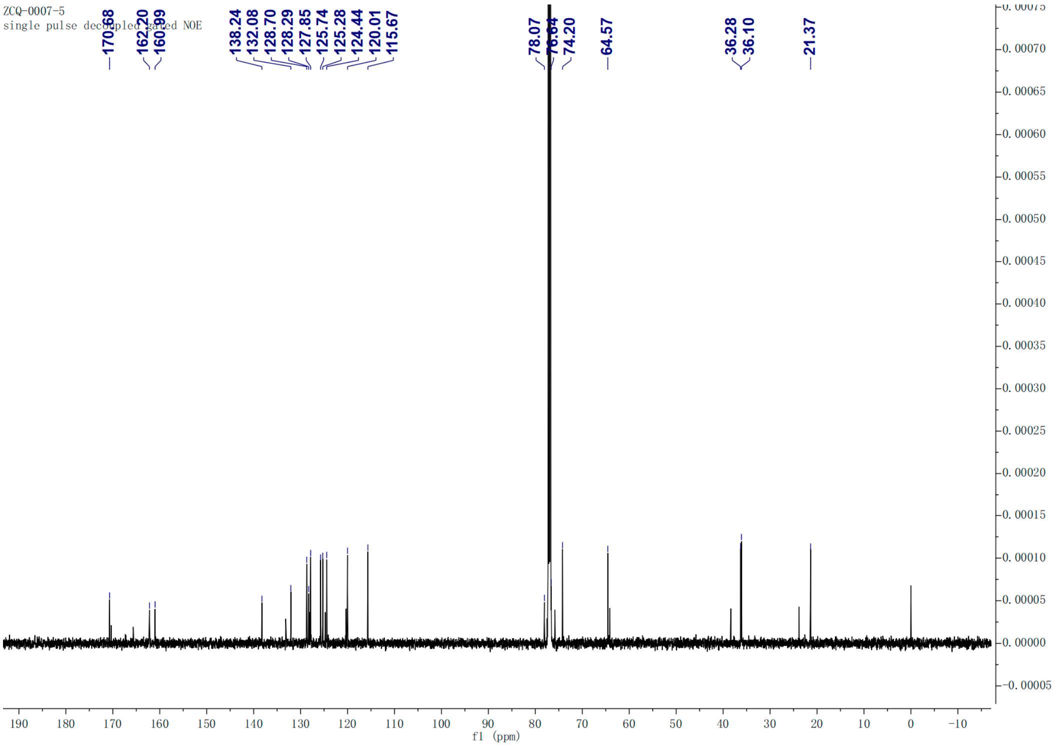

Figure S84. ^{13}C NMR (150 MHz, $CDCl_3$) spectrum of corallomycetellain J (**10**).

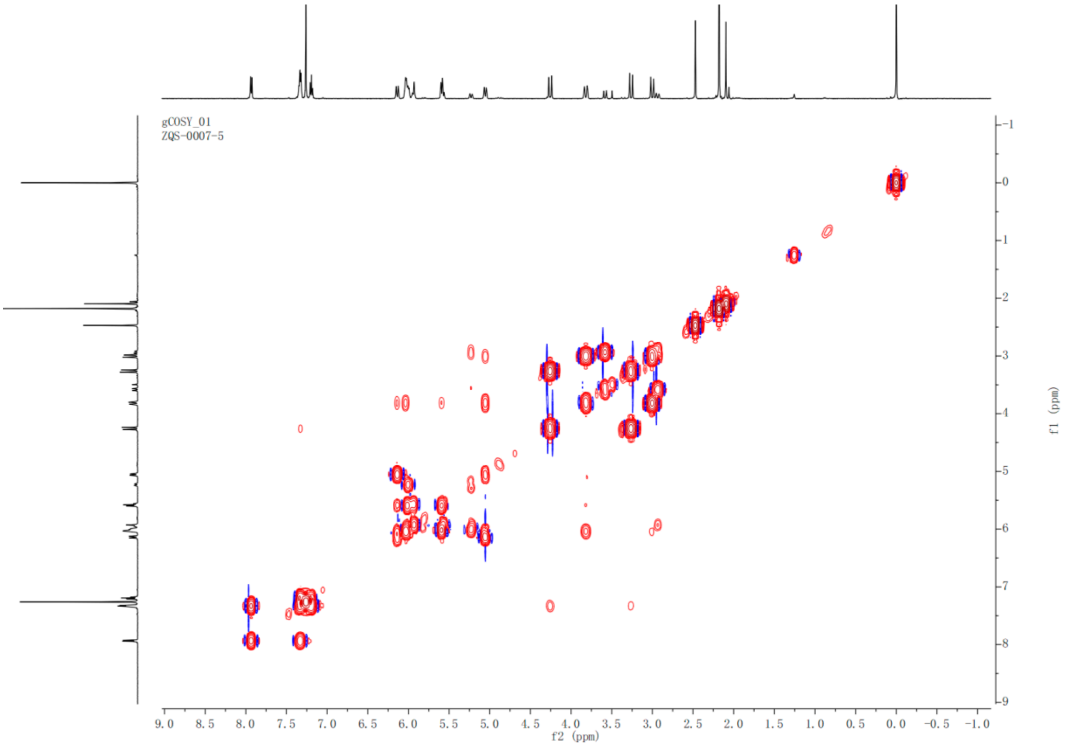

Figure S85. ^{1}H-^{1}H COSY spectrum of corallomycetellain J (**10**).

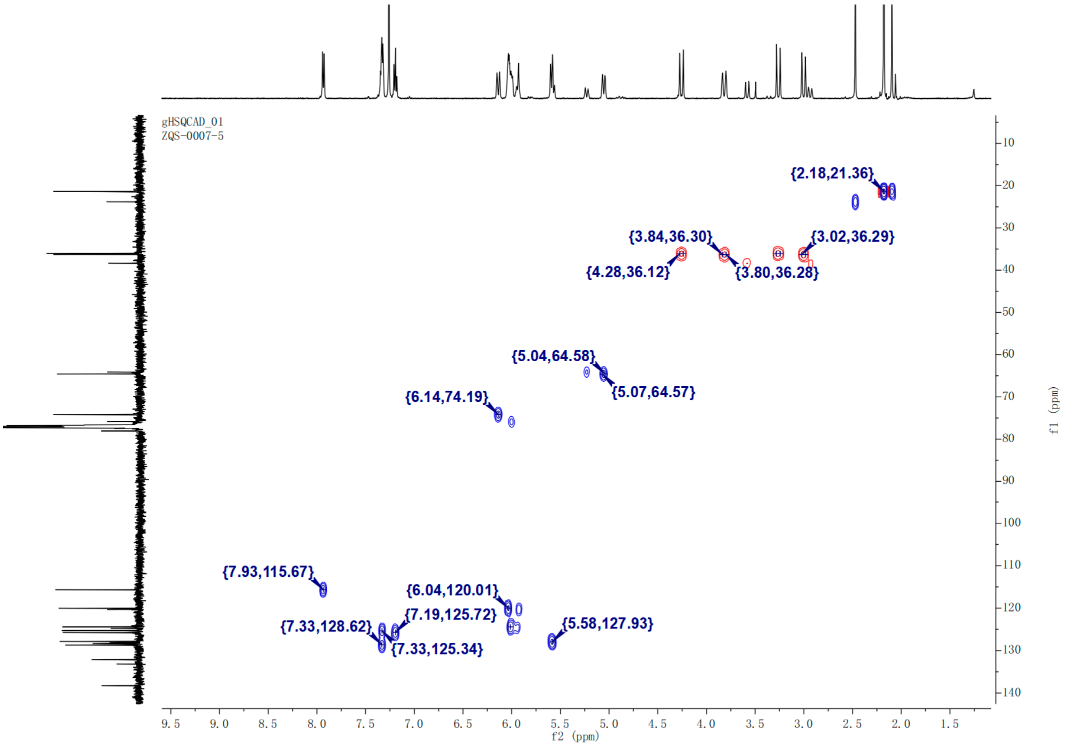

Figure S86. HSQC spectrum of corallomycetellain J (**10**).

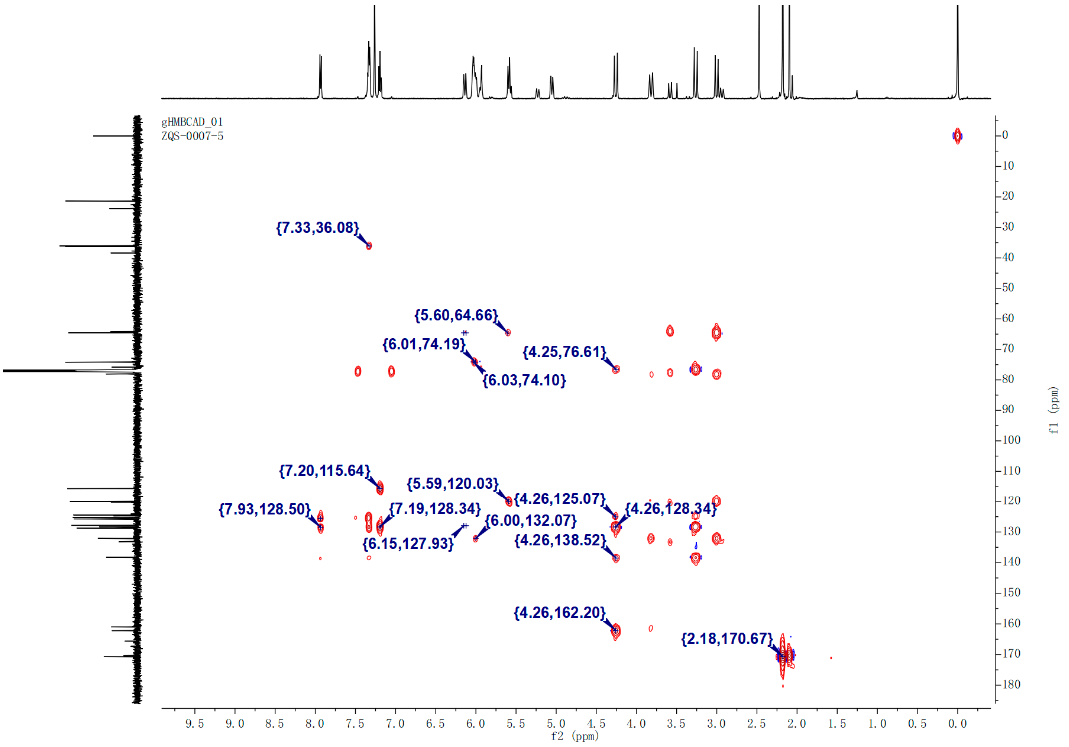

Figure S87. HMBC spectrum of corallomycetellain J (**10**).

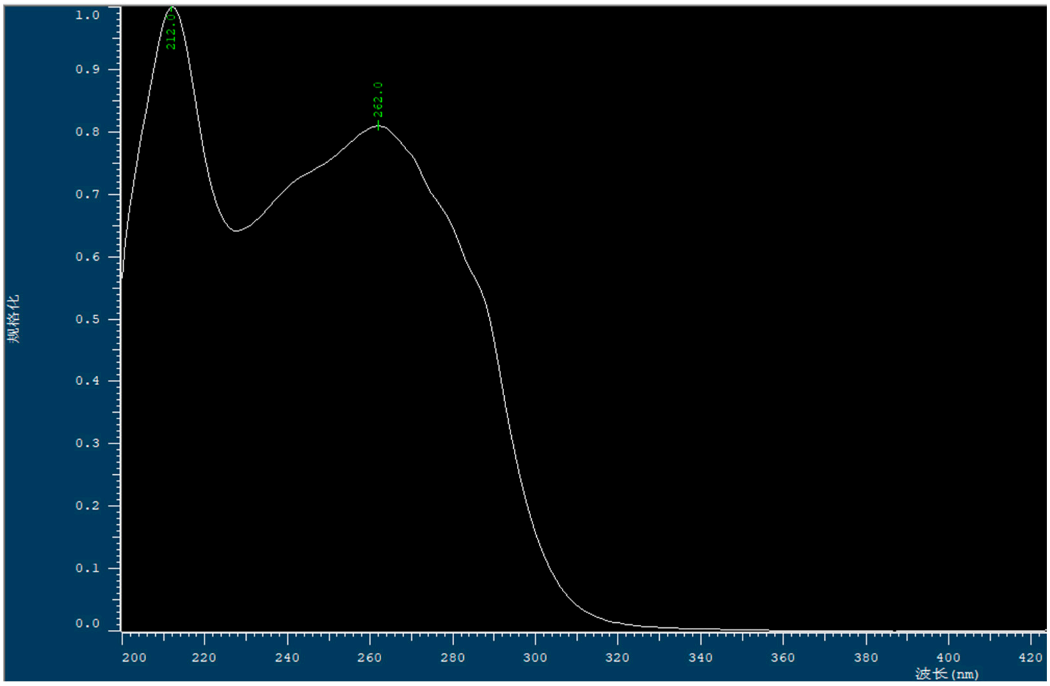

Figure S88. The UV spectrum of corallomycetellain J (**10**).

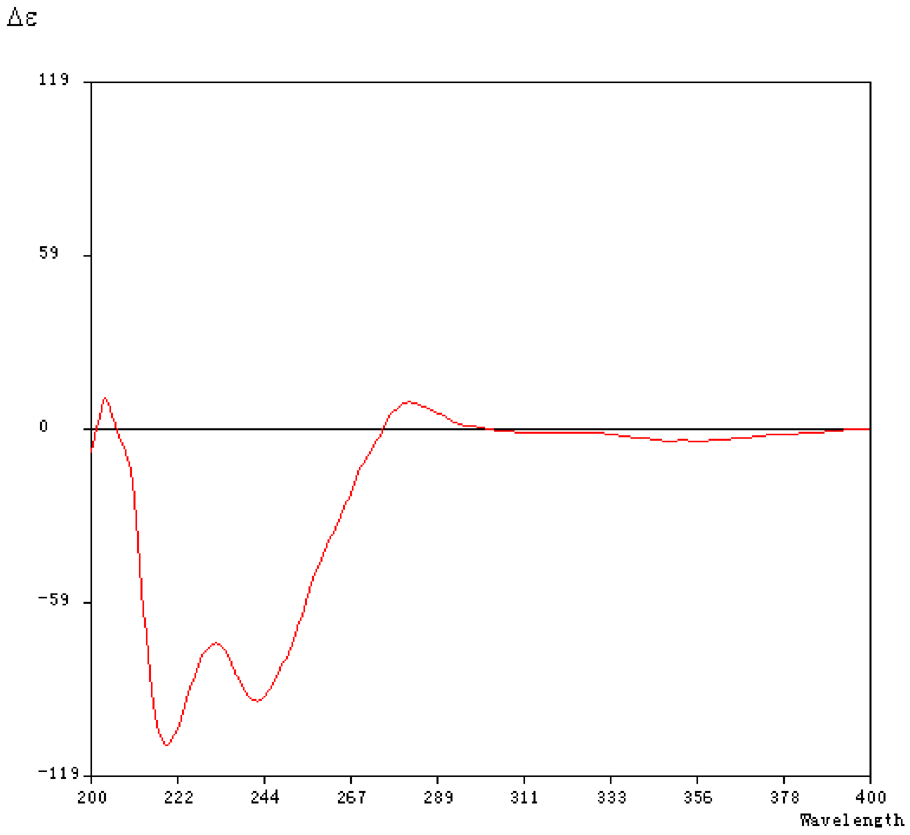

Figure S89. The CD spectrum of corallomycetellain J (**10**).

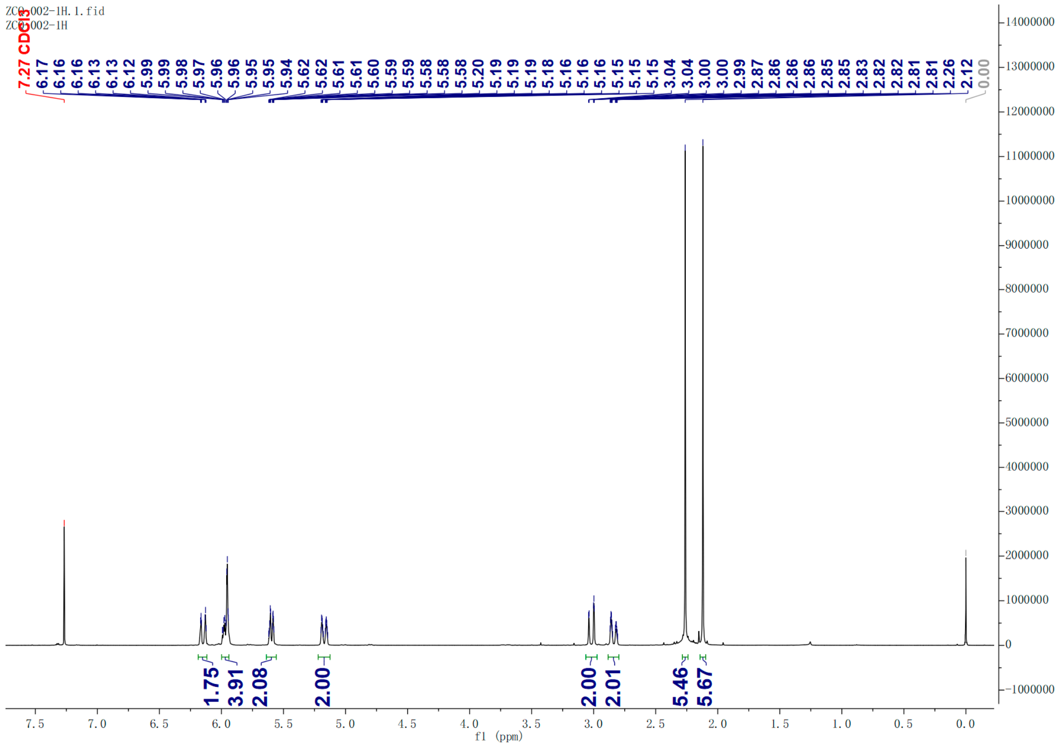

Figure S90. ^{1}H NMR (600 MHz, $CDCl_3$) spectrum of haematocin (**11**).

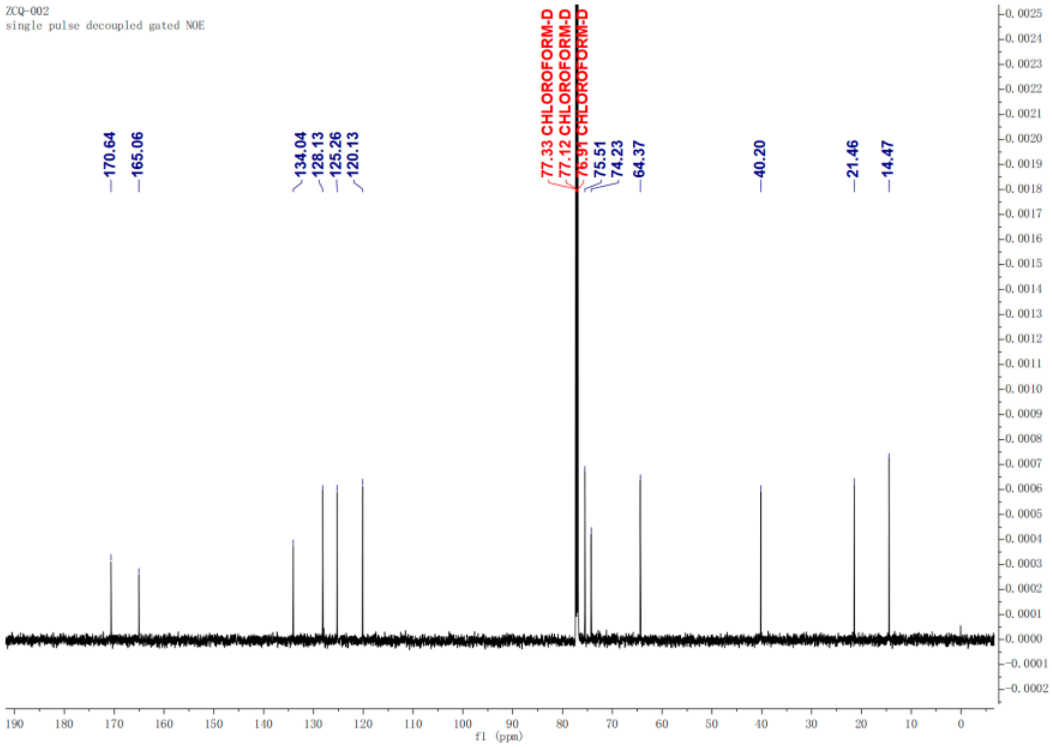

Figure S91. ^{13}C NMR (150 MHz, $CDCl_3$) spectrum of haematocin (**11**).

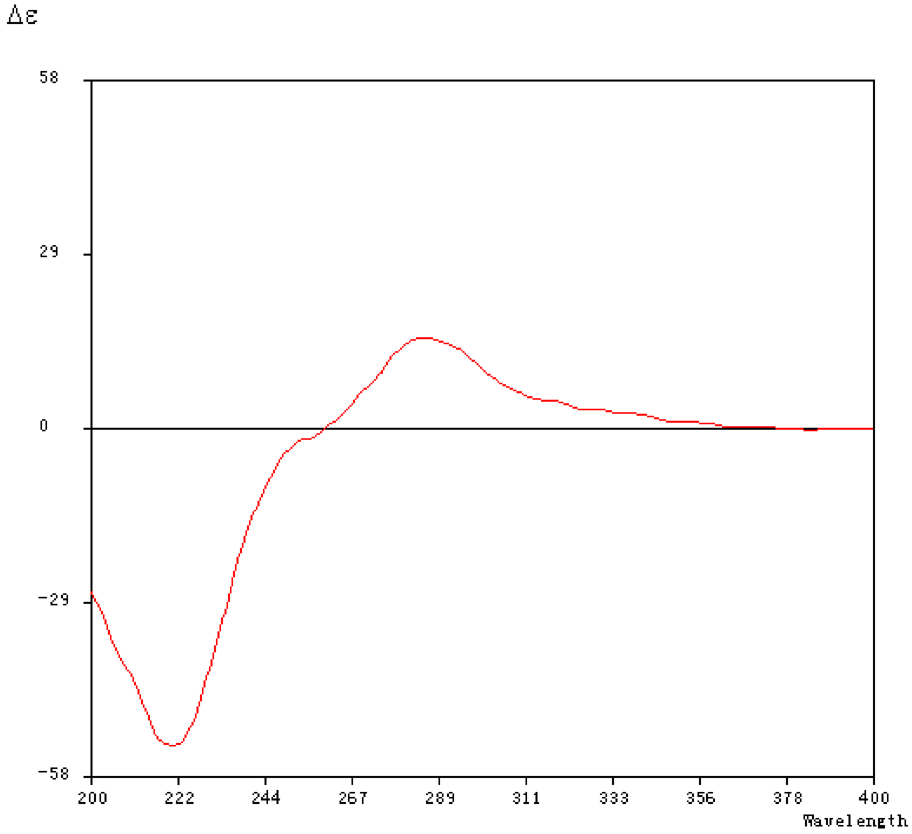

Figure S92. The CD spectrum of haematocin (**11**).

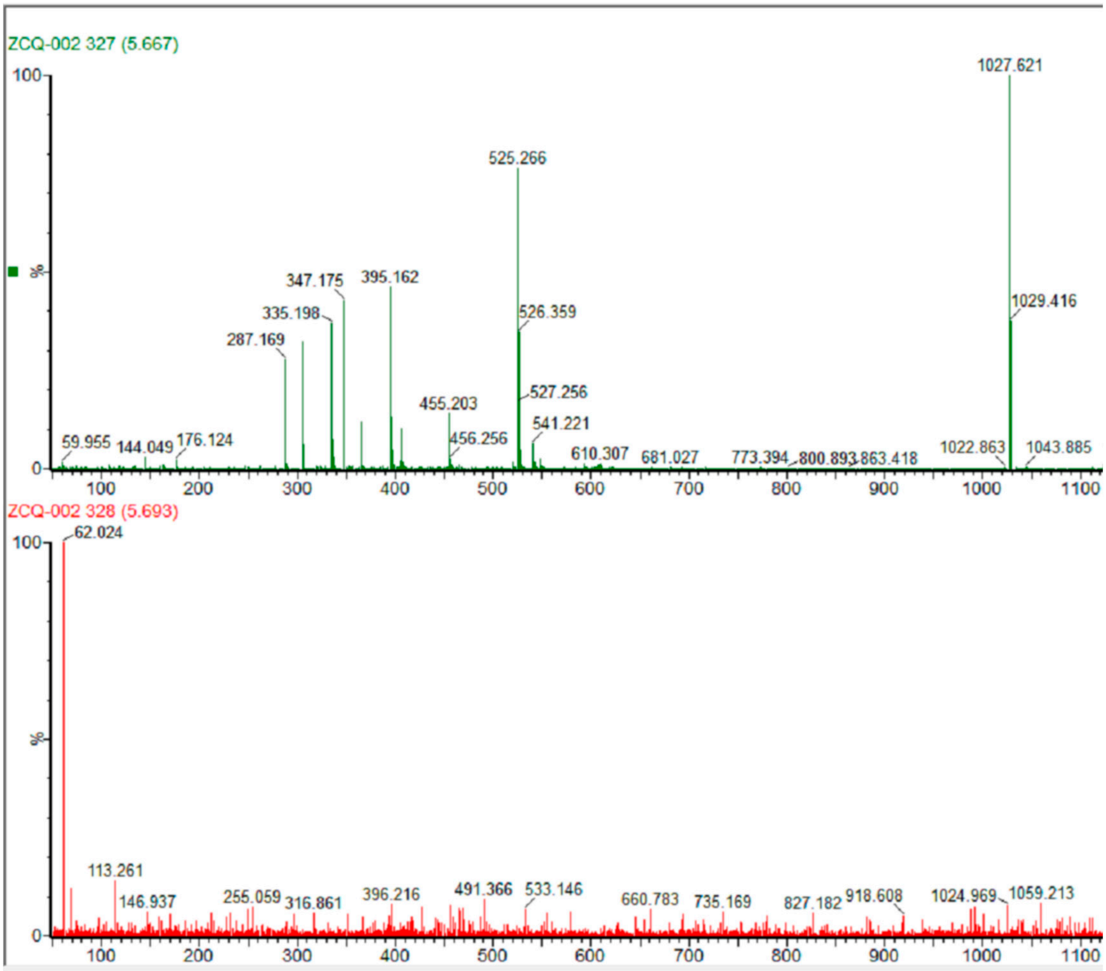

Figure S93. The UPLC-MS spectrum of haematocin (**11**).

Analytical HPLC was used to assess the purity of compounds **1-10**. The purity was determined to be > 95% by peak area normalization at 254 nm.

A gradient elution was applied using HPLC-grade methanol as mobile phase A and water containing 0.05% trifluoroacetic acid as mobile phase B over 45 minutes at a flow rate of 1.0 mL/min.

The gradient program was as follows:

0-5 min, 5% A;

5-35 min, 5% → 100% B;

35-40 min, 100% A;

40-45 min, 5% A.

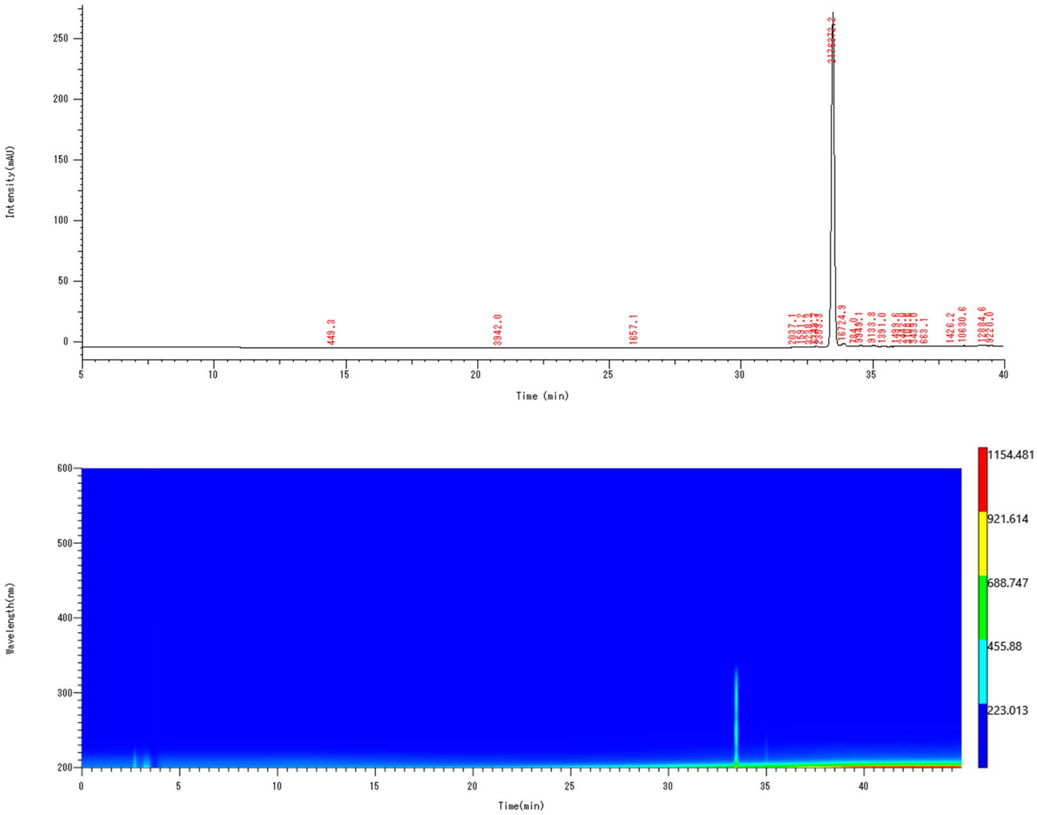

Figure S94. HPLC purity chromatogram of **1**.

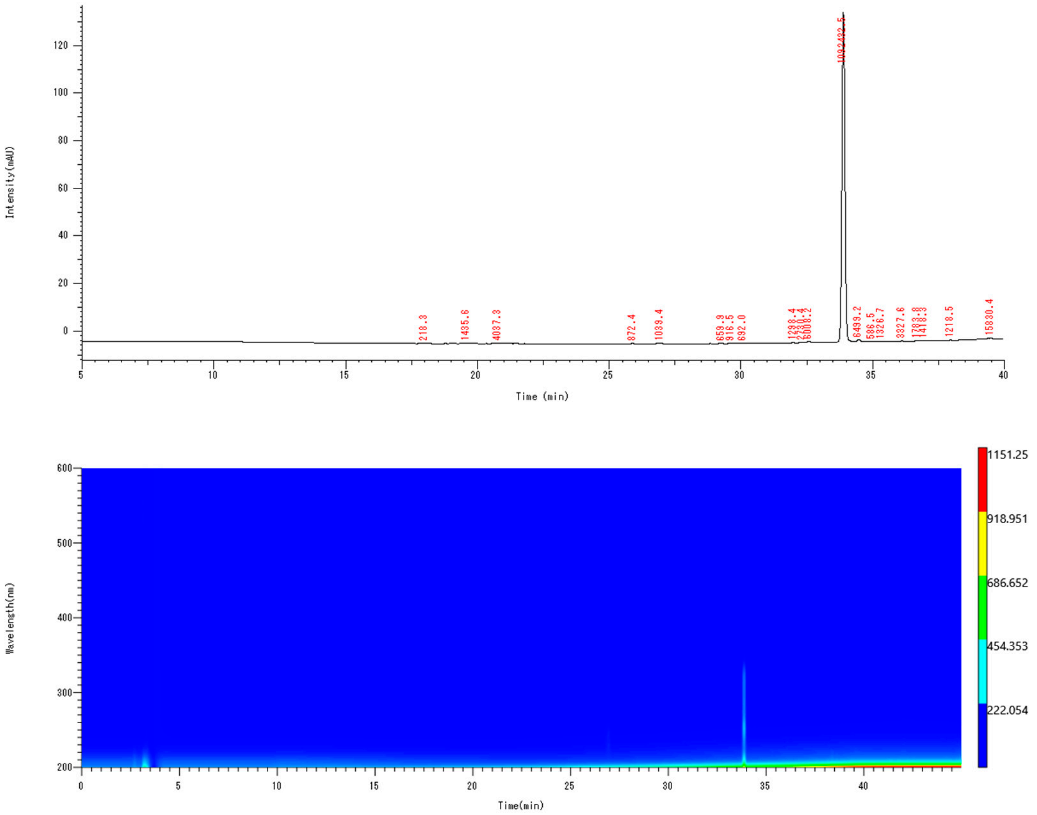

Figure S95. HPLC purity chromatogram of **2**.

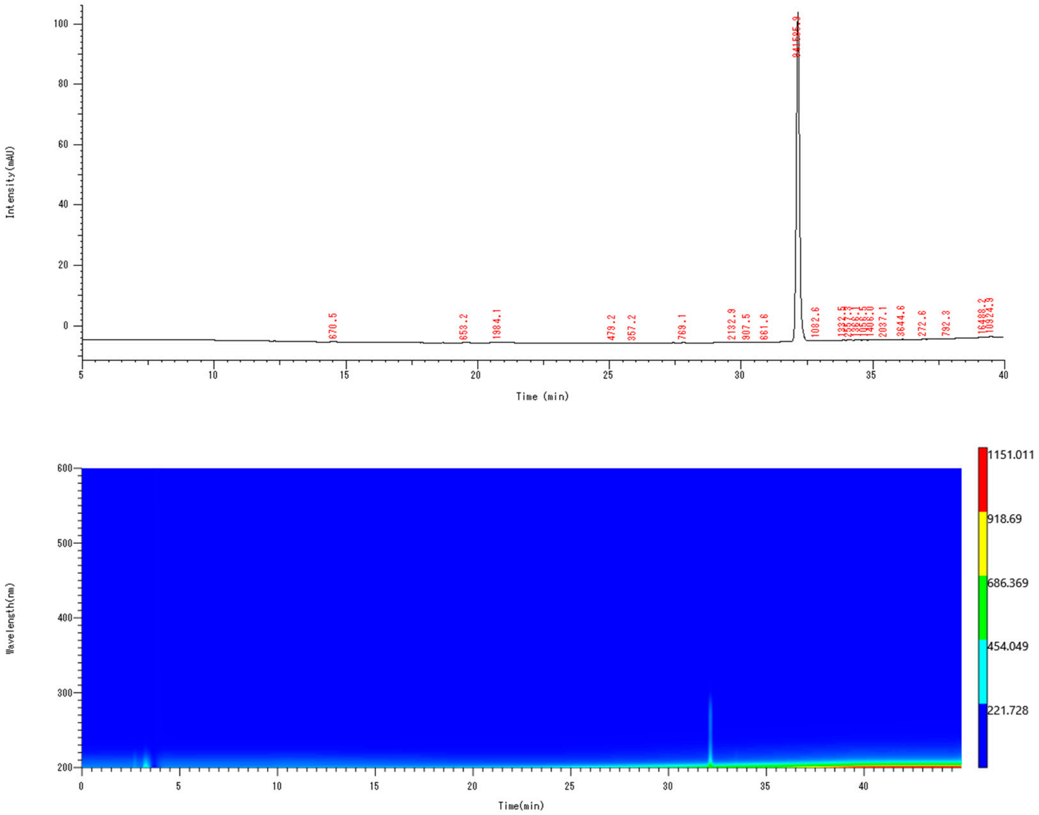

Figure S96. HPLC purity chromatogram of **3**.

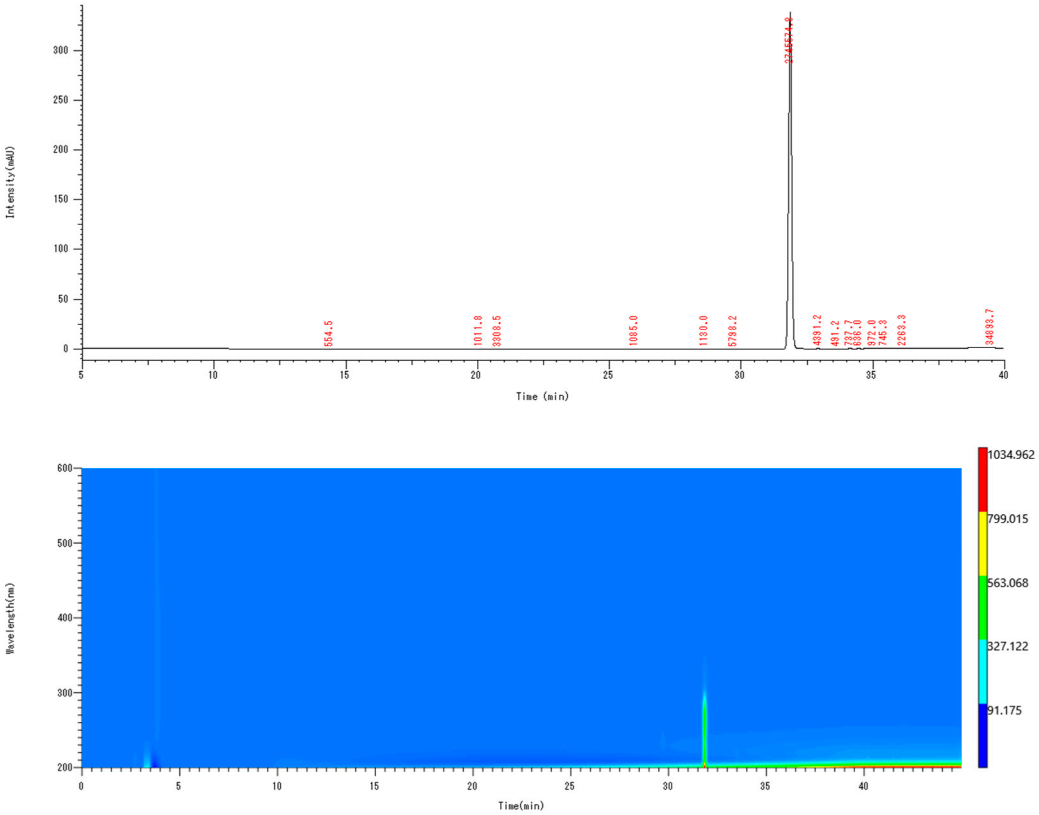

Figure S97. HPLC purity chromatogram of **4**.

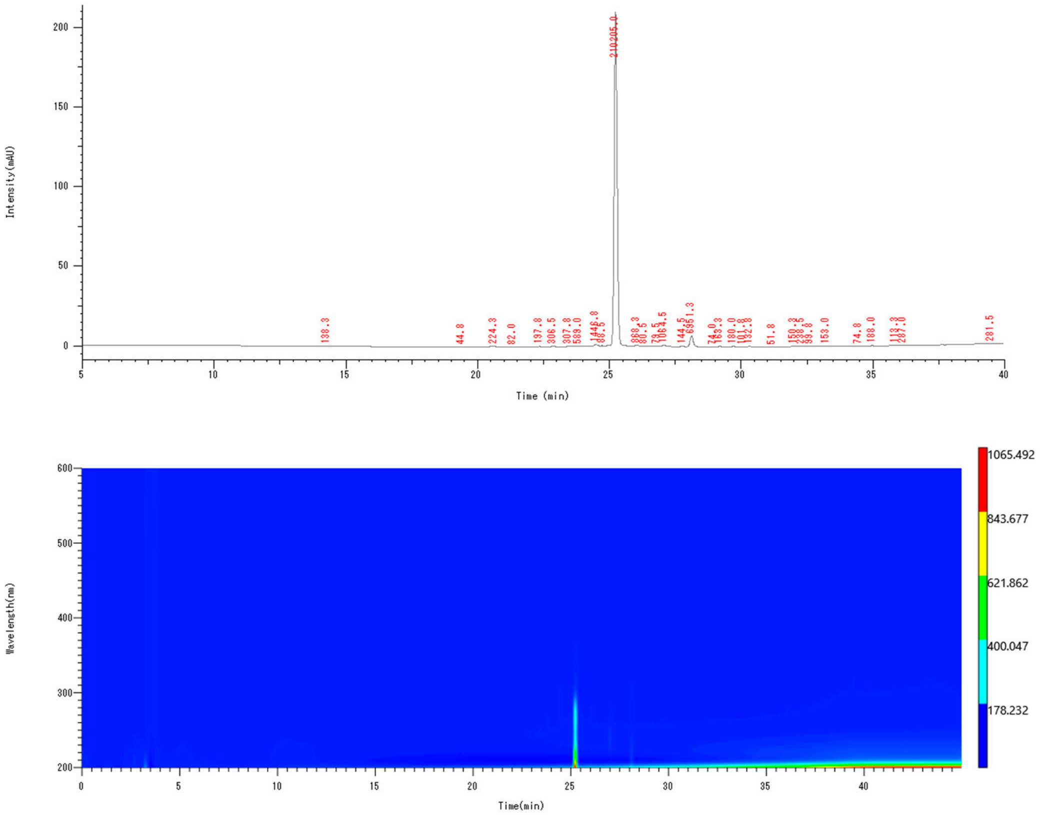

Figure S98. HPLC purity chromatogram of **5**.

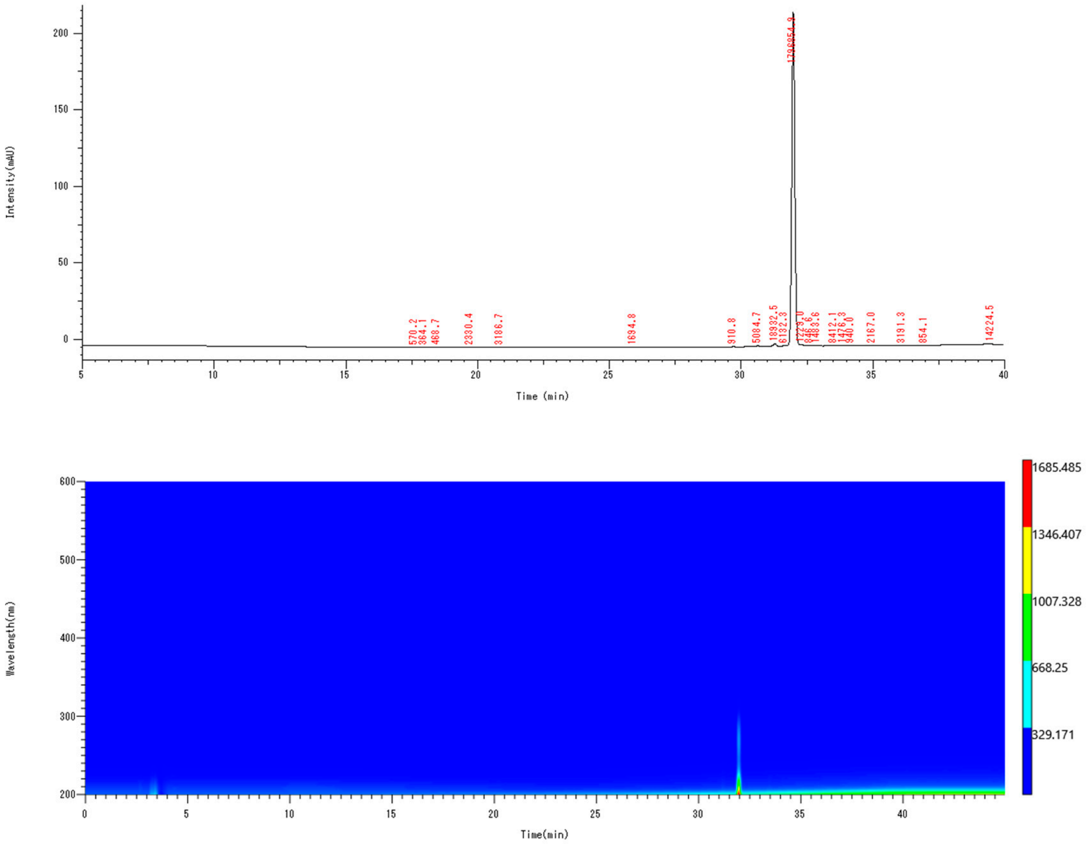

Figure S99. HPLC purity chromatogram of **6**.

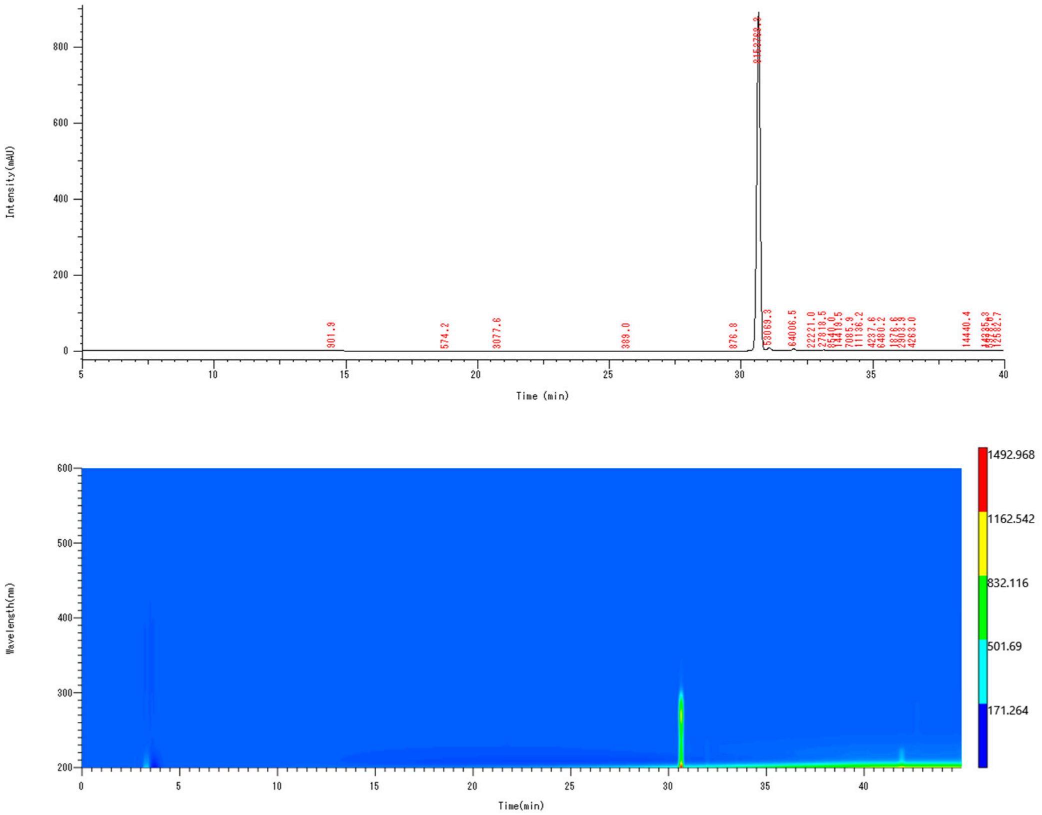

Figure S100. HPLC purity chromatogram of **7**.

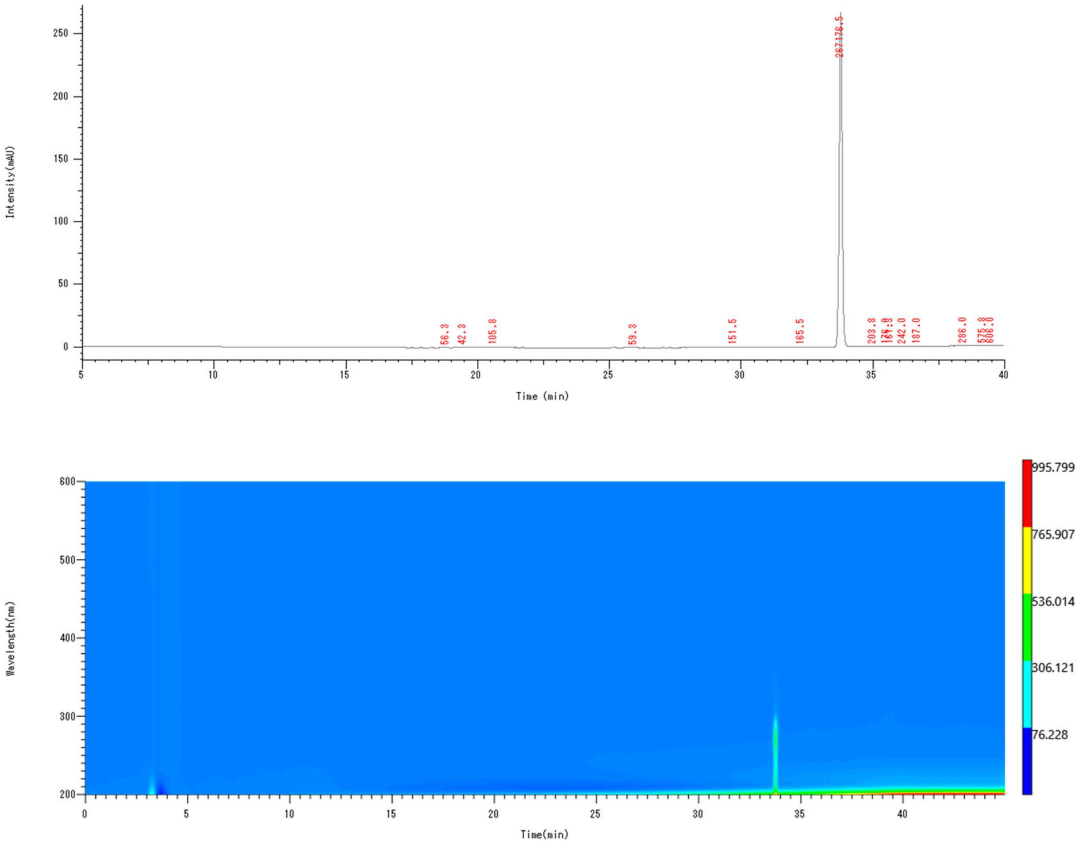

Figure S101. HPLC purity chromatogram of **8**.

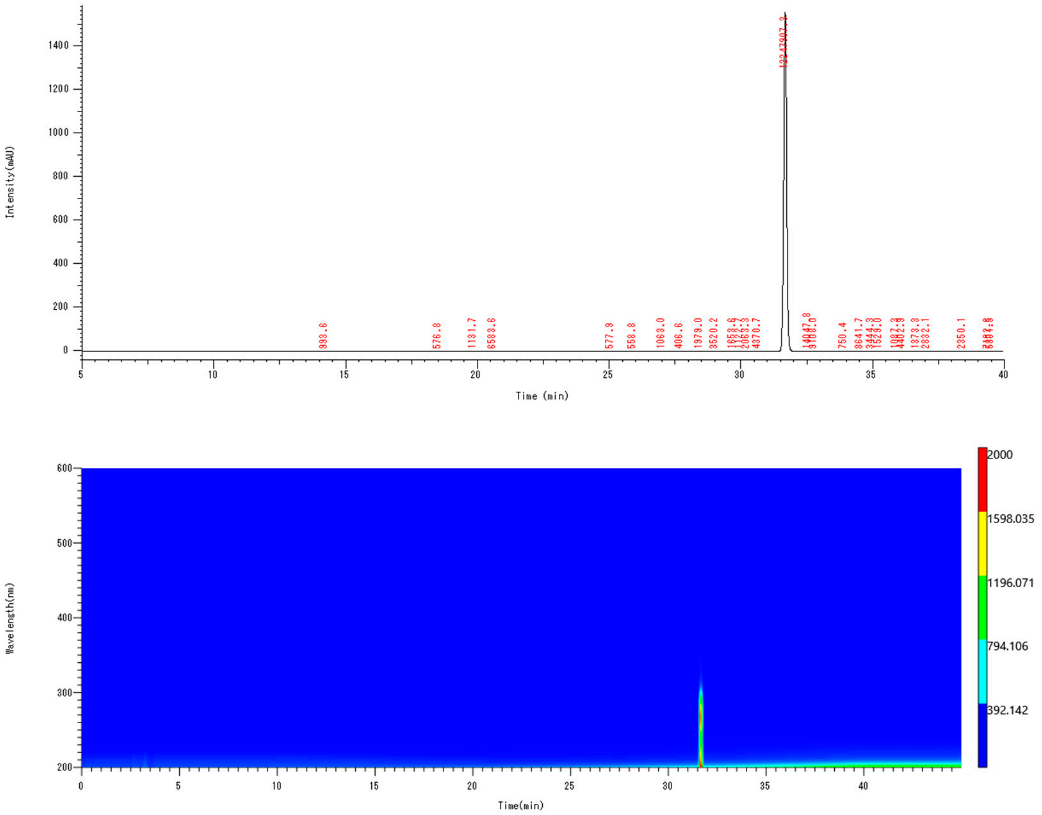

Figure S102. HPLC purity chromatogram of **9**.

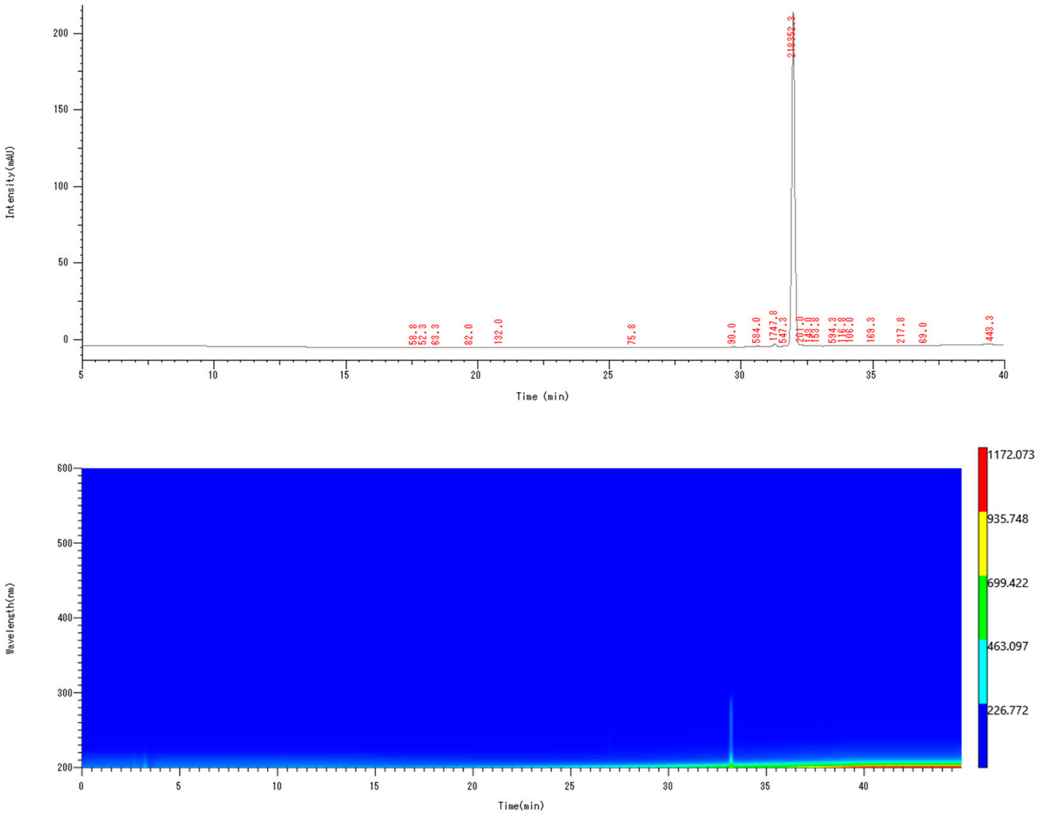

Figure S103. HPLC purity chromatogram of **10**.

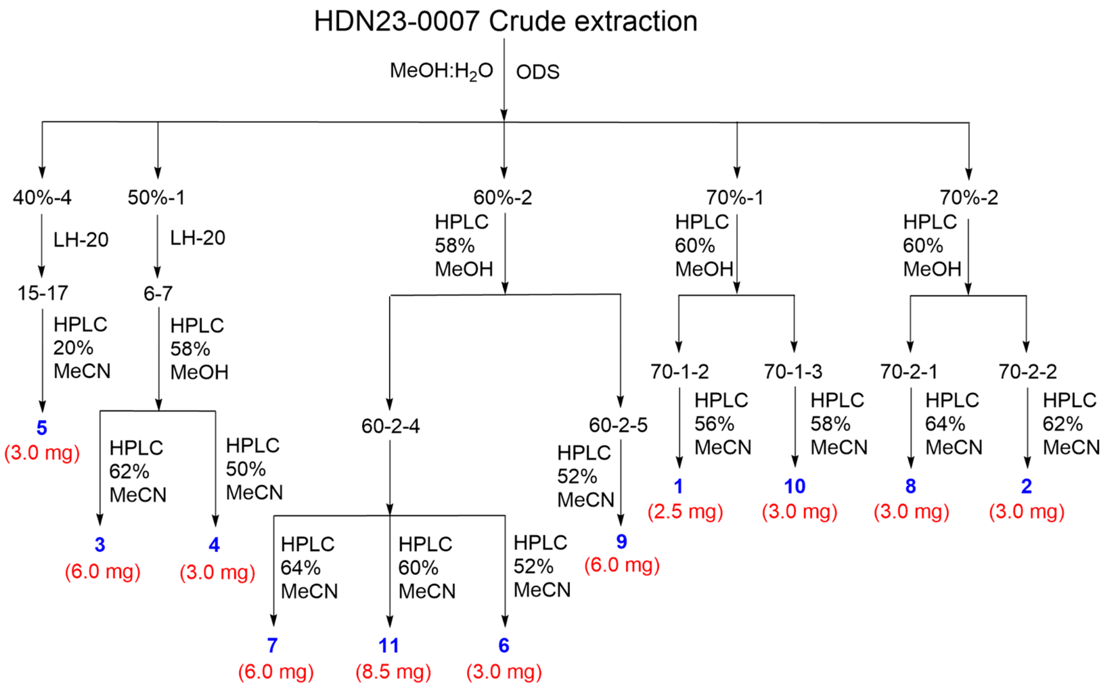

Figure S104. Isolation of compounds from the extract of fungus HDN23-0007.

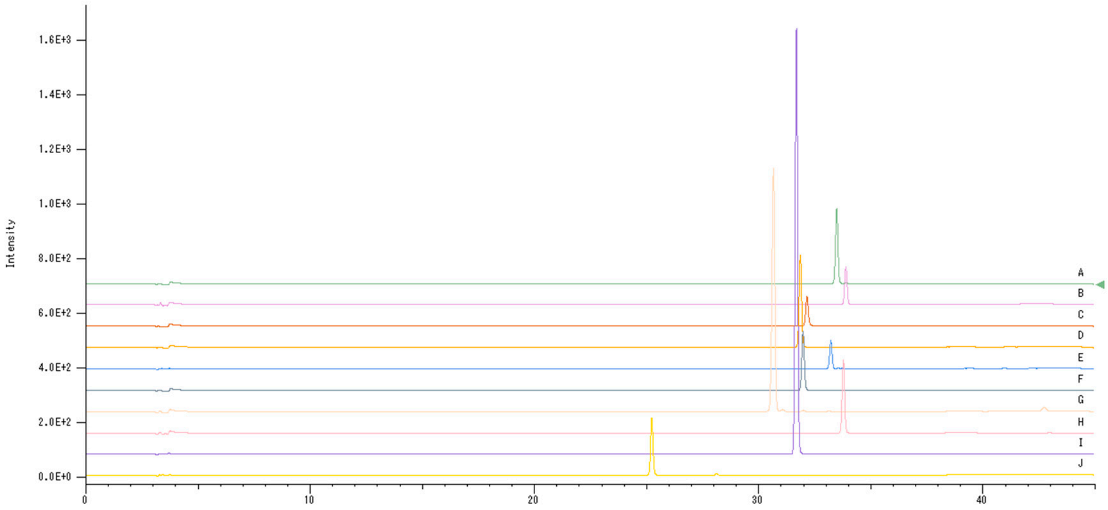

Figure S105. Comparative HPLC analysis of corallomycetellains A-J (**1**-**10**).

The ITS gene sequences data of *Corallomycetella repens* HDN23-0007.

TCTTCGTTGCCTCGGCGGGACTCGCCCCGGCGCCCCTCGGGCCCGGACCCAGGCG
CCCGCCGGAGGACCCAAACTCTTGTCTCTTGCGAGTATCTTCTGAGTGGCACAAG
CAAATAAAATCAAAACTTTCAACAACGGATCTCTTGGTTCTGGCATCGATGAAGA
ACGCAGCGAAATGCGATAAGTAATGTGAATTGCAGAATTCAGTGAATCATCGAAT
CTTTGAACGCACATTGCGCCCGCCAGTATTCTGGCGGGCATGCCTGTTCGAGCGTC
ATTTCAACCCCTCAGGCCCCTGGGCTTGGTGTTGGGGATCGGCCCGCCCCTGGCG
GCGGCGCCGGCCCCGAAATCGAGTGGCGGTCTCGCTGCAGCCTCCTCTGCGTAGT
AGCACACACCTCGCACCGGGACGCAGCGCGGCCACGCCGTGAAACCCCCAACT
CTCTCAAGGTTGACCTCGAATCAGGTAGGACTACCCGCTGAACTTAAGCAT
